# Electrochemical and Non-Electrochemical Pathways in the Electrocatalytic Oxidation of Monosaccharides and Related Sugar Alcohols into Valuable Products

**DOI:** 10.1021/acs.chemrev.4c00261

**Published:** 2024-10-31

**Authors:** Matthijs P. J. M. van der Ham, Jordi Creus, Johannes H. Bitter, Marc T. M. Koper, Paolo P. Pescarmona

**Affiliations:** † Biobased Chemistry and Technology, Wageningen Research, P.O. Box 17, 6700 AA Wageningen, The Netherlands; ‡ Leiden Institute of Chemistry, Leiden University, P.O. Box 9502, 2300 RA Leiden, The Netherlands; § Chemical Engineering Group, Engineering and Technology Institute Groningen (ENTEG), University of Groningen, Nijenborgh 4, 9747 AG Groningen, The Netherlands; ∥ TNO, Westerduinweg 3, 1755 LE Petten, The Netherlands

## Abstract

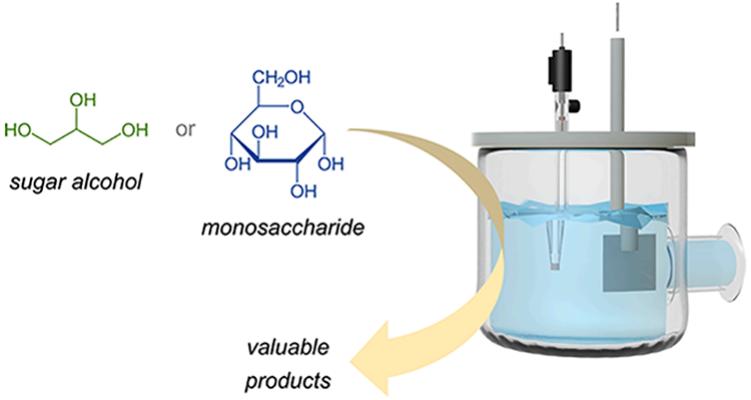

In this contribution, we review the electrochemical upgrading of saccharides (e.g., glucose) and sugar alcohols (e.g., glycerol) on metal and metal-oxide electrodes by drawing conclusions on common trends and differences between these two important classes of biobased compounds. For this purpose, we critically review the literature on the electrocatalytic oxidation of saccharides and sugar alcohols, seeking trends in the effect of reaction conditions and electrocatalyst design on the selectivity for the oxidation of specific functional groups toward value-added compounds. Importantly, we highlight and discuss the competition between electrochemical and non-electrochemical pathways. This is a crucial and yet often neglected aspect that should be taken into account and optimized for achieving the efficient electrocatalytic conversion of monosaccharides and related sugar alcohols into valuable products, which is a target of growing interest in the context of the electrification of the chemical industry combined with the utilization of renewable feedstock.

## 1 Introduction

Fossil-based resources account for 86% of the global energy demand and a staggering 96% of organic chemicals,^1^ accounting for 614 million tons of chemicals per year in 2013.^2−4^ These resources are however not sustainable and their use results in the emission of greenhouse, polluting gases (CO_2_, CH_4_, NO_X_, etc.), inflicting environmental and health damage. This requires the search for alternative energy sources and renewable carbon-based resources, which should be environmentally friendly, cheap, and readily available.^3,5^ In this regard, a switch from a fossil-based economy to an electrified and circular/biobased economy could contribute to a solution.^6^ In this context, electricity generated from renewable resources (e.g., wind, solar, hydropower) could serve as an energy source to drive catalytic reactions, while lignocellulosic biomass derived from non-edible agricultural waste streams could serve as a carbon source.^5,7−9^ This biomass is composed of carbon-based compounds with a wide range of functionalities (e.g., alcohol, ketone/aldehyde carboxyl groups),^10−12^ which offer a large range of possibilities to produce value-added (platform) chemicals.

Currently, most biomass-based feedstocks are processed on an industrial scale via fermentation, stoichiometric, or thermocatalytic routes. However, these processes tend to have a substantial impact on the environment.^11^ Fermentation processes are slow and require multiple expensive downstream steps,^13^ while stoichiometric processes generate substantial amounts of waste salts. Yet, these routes are still the main approaches utilized for the synthesis of biomass-based platform molecules like gluconic acid, lactic acid, and nitric acid.^13,14^ In this regard, the conversion of biomass-based feedstock via thermocatalytic routes seems an interesting alternative, considering that the catalyst, if heterogeneous, can be readily separated from the products and reused for several runs or for prolonged time on stream, and that no waste salts are generated as byproducts.^15,16^ However, thermocatalysis typically requires an energy input in the form of heat to surpass the activation barrier.^17,18^ Thermocatalytic redox processes may also require pressurized gaseous reactants such as O_2_ or H_2_. As an alternative, electrochemical and photochemical routes have been investigated as a sustainable and clean method to carry out the conversion of biobased compounds to useful products. Both routes can generate chemicals by separating oxidation and reduction reactions. Despite this similarity, electrochemical routes offer better perspectives toward practical application as the photochemical routes generally face challenges in converting light to chemical energy efficiently, maintaining photocatalyst stability, and developing scalable, cost-effective systems.^19^

For electrochemical reactions, a higher potential compared to what would be needed based on the thermodynamics of the reaction (i.e., an overpotential) typically needs to be applied to overcome the reaction activation barrier.^20−22^ Developing electrocatalysts is crucial to minimize this overpotential and thus to optimize the energy-efficiency of the electrochemical process. In general, electrochemical routes present several potential advantages over conventional chemical production routes: (1) the possibility of using ambient temperatures and pressures resulting in mild reaction conditions, (2) the use of H_2_O (OH^–^ or H^+^) as oxygen or hydrogen source for the oxidation/reduction allows circumventing the necessity of costly oxidizing/reducing agents, and (3) the ease of tuning the reaction conditions (e.g., by tuning the electrode potential) can enable easy control over the reaction rate and selectivity.^3,23^ Moreover, electrosynthesis could aid in leveraging the energy surplus generated by energy suppliers, which is subject to fluctuations depending on the availability of the sources. This is achieved by synthesizing compounds with chemical bonds containing a high amount of energy, which can then be used as feedstock in fuel cells.^17^ Electrosynthesis does not necessarily require an energy input, and if the redox reaction is thermodynamically favorable, it can be achieved in fuel cells that cogenerate electricity and chemicals.^24^ The latter is an additional asset compared to photochemical routes. Furthermore, the electrochemical oxidation of biobased compounds has been investigated as an alternative anodic reaction to replace the oxygen evolution reaction (OER) in electrolyzers used for green hydrogen production.^25^ This approach is referred to as hybrid water electrolysis (HWE) and can have a 2-fold benefit: decrease the energy input and generate a more valuable anodic product than O_2_.

**Figure 1:**
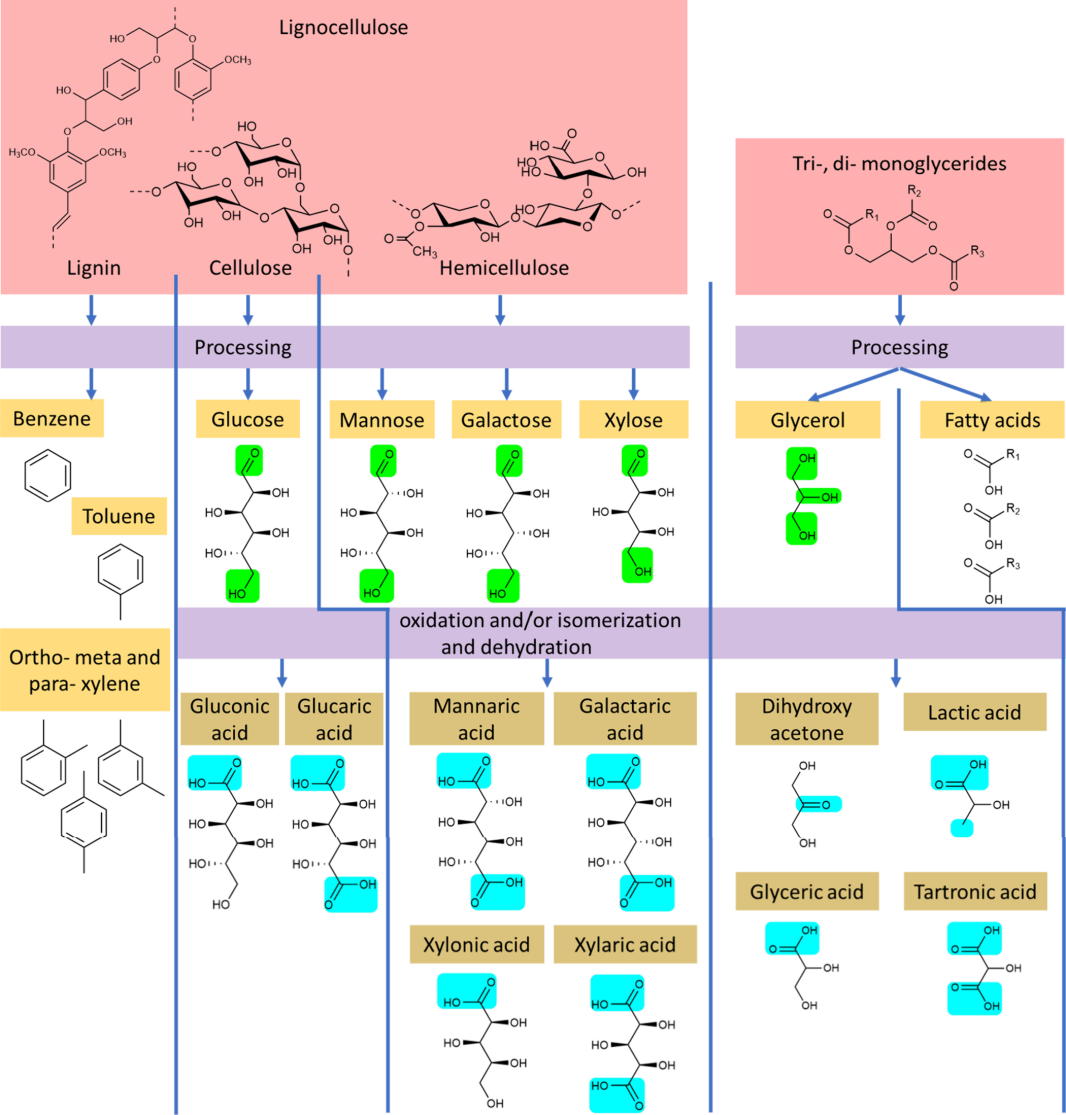
Overview of lignocellulose and fats/oils (red) that can be fractionated to various molecules (yellow). These molecules (e.g., saccharides and sugar alcohols) have common functional groups (green) that typically can undergo oxidation and/or isomerization and dehydration (blue) to obtain value-added platform molecules (gold).

The electrocatalytic valorization of biomass-based feedstock (e.g., saccharides and sugar alcohols) derived from fats, oils, and lignocellulose to obtain value-added platform molecules has been widely investigated (Figure 1).^26−30^ Fats and oils can be obtained from edible crops, waste cooking oils, or oleaginous microorganisms and commonly consist of glycerol linked through ester groups to one to three fatty acids, forming a triglyceride.^31^ These triglycerides can be subjected to hydrolysis (or transesterification) to produce free fatty acids (or esters) and glycerol. The obtained free fatty acids/esters can be used as biodiesel, while glycerol remains a byproduct,^31^ accounting for 4.2 million tons per year.^32^ Lignocellulosic biomass derived from agricultural waste has an even greater potential due to its abundance, accounting for 1470 million tons per year for rice straw, wheat straw, corn straw and bagasse.^33^ These lignocellulosic biomass waste streams consist of three main components: cellulose (32–47%), hemicellulose (19–35%), and lignin (5–30%), with the remainder being minor fractions of proteins and ash.^33,34^ Cellulose forms crystalline bundles of fibers that are intertwined by hemicellulose and lignin polymers, making it difficult to access and thus process. Hemicellulose is a branched polymer and has an amorphous structure, which makes it the easiest to hydrolyze (via an acid hydrolysis step) to yield monomeric constituents.^35^ The composition of hemicellulose is highly dependent on the source and may contain various saccharides (glucose, mannose, galactose, rhamnose, and xylose) and uronic acids. The fractionation of cellulose from lignocellulose is applied on an industrial scale via Organosolv and Kraft processes to obtain cellulose pulp.^36^ Cellulose pulp has several commercial applications in the paper and building industry but can also be subjected to an acid or enzymatic hydrolysis step to generate glucose monomers.^35,36^ Lignin is a polymer based on phenolic units and with an amorphous structure. Its recalcitrant and complex chemical structure hinders its depolymerization and fractionation into phenolic compounds, which could be then upgraded to BTX compounds (benzene, toluene, and xylene).^37^ Currently, biorefinery technology is more advanced for the conversion of triglycerides and of the cellulosic and hemicellulosic fractions of lignocellulose, and therefore, the most commonly obtained platform molecules derived from biomass-based feedstock are saccharides (e.g., glucose, xylose) and sugar alcohols (e.g., glycerol).

Figure 1 highlights several key molecules (e.g., gluconic acid, glucaric acid, xylonic acid, and lactic acid) that are recognized by the National Renewable Energy Laboratory as top value-added platform chemicals in various industrial applications.^38^ Gluconic acid finds extensive use in the food, pharmaceutical, and paper industries. Glucaric acid serves as a corrosion inhibitor and metal complexing agent, and it can also be utilized in pharmaceuticals or as a precursor for biodegradable polymers.^39^ Both glucaric acid and related compounds like mannaric acid and galactaric acid show promise as precursors for adipic acid, a key material in nylon production.^40^ Xylonic acid serves multiple functions, including as cement plasticizer, as additive for improving vitamin C absorption, and as a component in copolymerization with polyamides and polyesters. It can also serve as a precursor for valuable chemicals such as ethylene glycol and glycolic acid.^41^ Lastly, lactic acid is pivotal in the production of poly lactic acid, a biodegradable biopolymer widely used in the food and pharmaceutical industries.^42^ Overall, these platform chemicals are crucial not only for their specific uses but also as key building blocks in chemical and material sciences.

The electrochemical oxidation of sugar alcohols^12,30,43−49^ and saccharides^50,51^ typically employs metals as electrocatalytic active species. This field of research has recently gained special attention in the (electro)catalysis community, resulting in a growing number of scientific publications. However, current review papers on the electrocatalytic oxidation of sugar alcohols are limited to the selective conversion of glycerol, lacking comparison with other sugar alcohols such as erythritol,^52^ arabitol,^53^ or sorbitol.^52,54−58^ On the other hand, the selective electrocatalytic oxidation of glucose and other saccharides has been less extensively reviewed,^50,51^ with most reviews on electrochemical oxidation of saccharides being focused on electrocatalyst activity for fuel cell research.^24,59,60^ Sugar alcohols and saccharides both contain primary and secondary alcohol groups, while saccharides also have an aldehyde group (in the linear form) or the corresponding anomeric carbon group (in the cyclic form). This makes it interesting to evaluate whether the reaction conditions and/or the electrocatalyst properties affect the selective conversion of the two types of reactants in a similar way.

Here, we critically review the literature on the electrocatalytic oxidation of sugar alcohols and saccharides, seeking trends in the effect of reaction conditions and of the design of metal-based electrocatalysts on the activity and selectivity in the oxidation of specific functional groups toward value-added compounds. The trends have been divided according to (a) the effect of reaction conditions on the electrocatalytic conversion of monosaccharides and sugar alcohols (section 2); (b) the potential-dependent state of the metal (e.g., Au and Pt) surface (section 3); (c) the relation between the features of the electrocatalysts (type of metal, metal oxidation state, exposed facets, and mono- vs bimetallic nature) and their performance in the oxidation of sugar alcohols (under various reaction conditions); see section 4; and (d) the relation between the features of the electrocatalysts and their performance in the electrocatalytic oxidation saccharides (under various reaction conditions); see section 5.

The focus is on glycerol and glucose as reference molecules to study the reactivity of C3–C6 sugar alcohols and C3–C6 saccharides. Additionally, we look for trends between the two types of reactants, aiming at defining which set of reaction conditions and electrocatalyst properties can favor the selective conversion of each functional group to specific value-added products. This review also aims at providing a critical assessment of the current literature, as well as to propose alternatives for sugar alcohol oxidation based on saccharide oxidation and vice versa. By shedding light on the reaction pathways for the electrocatalytic oxidation of sugar alcohols and saccharides, we aim at clarifying how these pathways can be steered toward the desired products. This approach will enable future research to design catalysts and electrochemical cells that can enhance the activity and direct the selectivity toward desired products, thereby overcoming the main current challenges in the electrocatalytic oxidation of sugar alcohols and saccharides.

It is worth reporting that the electrochemical oxidation of sugar alcohols and saccharides can also be carried out in the presence of an organic mediator (e.g., TEMPO or other nitroxide-based compounds), which undergoes a redox reaction with the biobased compounds and then gets reoxidized on the surface of the metal electrocatalyst. However, this is a conceptually different field of research in which the selectivity can be determined by other factors than the nature of the metal electrocatalyst, which is the focus of this review. Additionally, the use of mediators increases the challenges in terms of upscaling, implementation, and downstream processing. The interested reader can find more information about mediated electrochemical oxidation in several recent reviews and papers.^61−66^

## 2 Effect of Reaction Conditions on the Electrocatalytic Conversion of Monosaccharides and Sugar Alcohols

In this section, the influence of various reaction conditions on the electrochemical oxidation of sugar alcohols and monosaccharides is discussed by comparing and rationalizing the behavior of different electrocatalysts that have been reported. The reaction parameters that have been found to influence the behavior of a certain electrocatalyst in the conversion of monosaccharides and sugar alcohols are (a) the reaction conditions, e.g., the pH and type of ions of the electrolyte (section 2.1), the reaction temperature (section 2.2), and reactant concentration (section 2.3); (b) the potential-dependent state of the metal (e.g., Au and Pt) surface (section 3); and (c and d) the intrinsic properties of the electrocatalyst, e.g., type of metal, metal oxidation state, exposed facets, and mono- vs bimetallic nature, under various reaction conditions (section 4 for the oxidation of sugar alcohols; section 5 for the oxidation of saccharides). First, varying these parameters can lead to a change in the configuration of the reactant, for instance by opening a pyranose ring or by forming an anionic form of a sugar alcohol. Second, the reactant or products can undergo non-electrochemical conversion in the bulk of the solution, through isomerization, dehydration, and/or retro-aldol reactions. Third, the reaction conditions can also affect the oxidation state and surface chemistry of the electrocatalyst employed, and thus its performance. Altogether, these three effects influence the interaction between the reactant and the electrocatalyst, thus contributing to its activity, selectivity, and stability. Hence, it is crucial to understand the nature of such influences in order to gain control on, and thus optimize, the performance of an electrocatalytic system. The observed trends in selectivity were mainly obtained from electrocatalysts based on Au and Pt. It is worth mentioning that the cell design also plays a crucial role in defining the performance of an electrocatalytic system. However, this parameter is not systematically addressed in the literature and will not be discussed further in this review.

### 2.1 Effect of pH and Electrolyte Ions on the State of Reactant and Products

#### 2.1.1 Effect of pH and Role of Non-Electrochemical Reactions

The pH can influence the structure and, if applicable, the neutral vs ionic form of the reactant, which in turn affects the electrocatalyst performance. The pH is also related to the concentration of protons and hydroxide ions in solution, which can act as homogeneous catalysts and thereby promote non-electrochemical conversion of the reactant and products in the solution. In this section, the influence of pH on the solution-phase reactions of the reactants and products is discussed, whereas its effect on the electrocatalyst selectivity will be discussed in sections 4 and 5. It is important to discuss this effect separately and at the outset of the review, as we believe that the influence of (pH-dependent) solution-phase reactions on activity and especially selectivity has been underestimated in many literature reports.

The electrocatalytic oxidation of sugar alcohols and monosaccharides has been studied under acidic conditions (typically at pH = 1), neutral conditions, or alkaline conditions (pH > 7), with the last option being by far the most common.^54,56−58,67−69^ It has been proposed that alkaline conditions are more suitable for these oxidations because a basic medium promotes the formation of electroactive species like alkoxides or enediols via non-electrochemical reactions in the electrolyte solution, which then more easily adsorb and react on the electrocatalyst surface, thereby promoting the catalytic activity.^28,69−72^

**Scheme 1:**
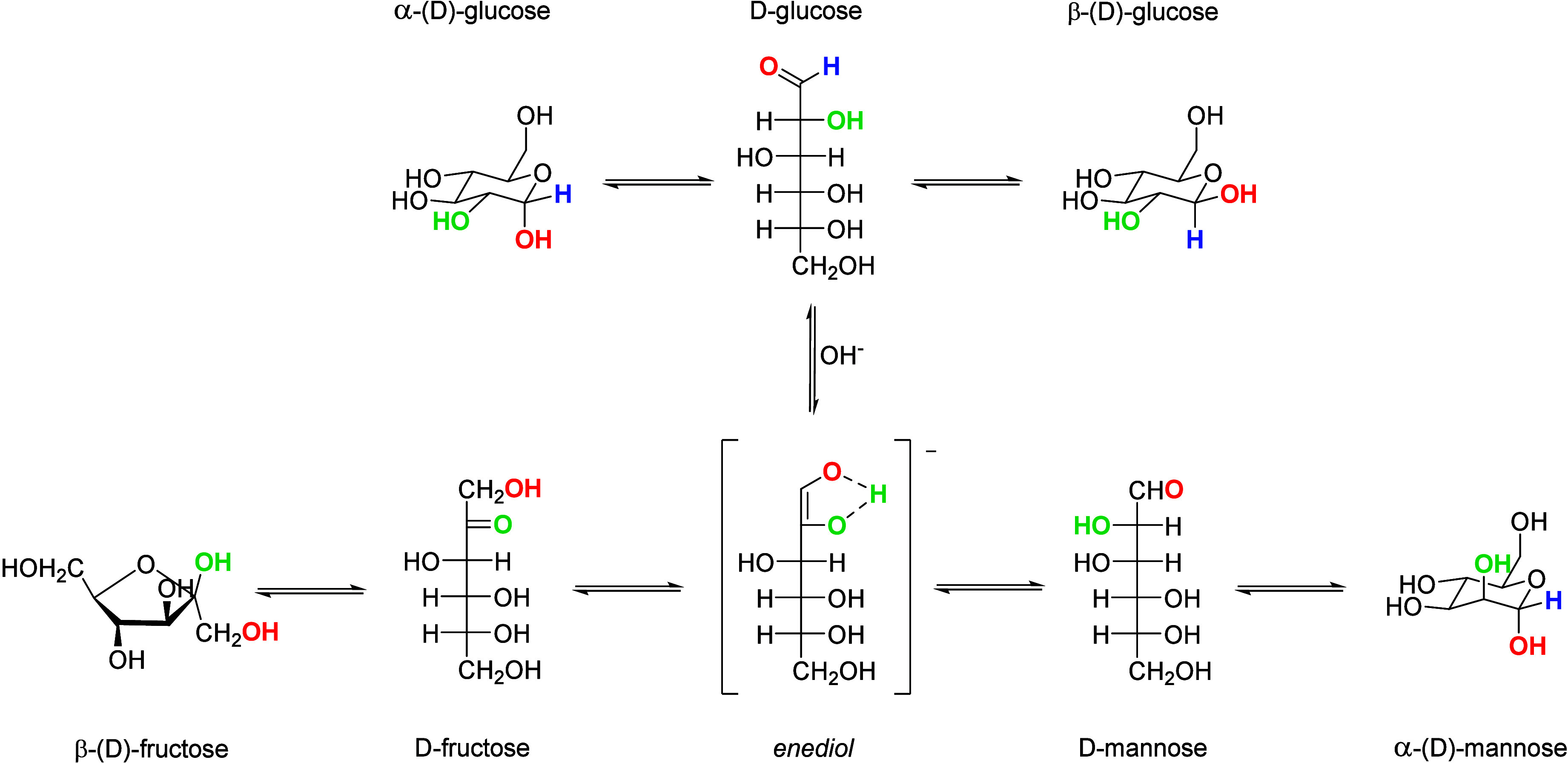
Reaction Scheme Displaying the Non-Electrochemical Isomerization Reactions Taking Place in the Electrolyte in the Presence of a Base

At neutral pH, the β-anomeric form of glucose is the major equilibrium species (63–67%), followed by the α-anomeric form (33–37%) and a minor fraction of the linear form (<0.03%).^70,73,74^ The β-anomeric form is the dominant species as it has its alcohol group in the equatorial position (Scheme 1), which decreases the steric hindrance,^75^ and it has an increased number of hydrogen bonds,^73^ making it thermodynamically the most stable form. Importantly, under acidic conditions, the β-anomeric form was found to be the most reactive species for electrocatalytic oxidation reactions over Pt electrodes.^70^ β-d-Glucose has its anomeric C–H bond at the axial position, whereas α-d-glucose has its anomeric C–H bond at the equatorial position (Scheme 1). With the anomeric C–H bond at the axial position, all the OH groups remain in equatorial position, which favors a planar approach of the β-d-glucose molecule to the electrocatalyst surface due to a lower steric hindrance, thus promoting the adsorption of the reactive species and enhancing the electrocatalytic activity.^70^ More recently, Holade et al. showed that under neutral conditions the oxidation of the α-d-glucose on Au proceeds faster than that of β-d-glucose.^76^ In contrast to Largeaud et al., Holade et al. argue that the catalytic activity toward α-d-glucose is higher since the α-anomeric form with its C–OH bond in the axial position more easily adsorbs on the Au surface, which was based on DFT calculations.^76^ The ratio between the α-anomeric form and β-anomeric form is dependent on the pH of the solution.^74^ Under acidic and alkaline conditions the α-anomer:β-anomer ratio is 45:55 and 10:90, respectively.^74^ Moreover, the mutarotation rate from the linear d-glucose to the β-anomer is higher under alkaline conditions, followed by acidic conditions (pH < 2) and then neutral conditions.^70,77^ The fast mutarotation of glucose under alkaline conditions made it impossible to distinguish the reactivity of the different anomeric structures.^74^ Hence, the time between initiation of the experiment and the addition of d-glucose might affect the catalyst activity that is measured.

In alkaline electrolytes (11 < pH < 12), a sharp increase in electrocatalytic activity was observed for the oxidation of saccharides over Cu^78^ and sugar alcohols over Au.^79,80^ This effect was attributed to the formation of the anionic species upon deprotonation of glucose, yielding an enediol species (Scheme 1), or upon deprotonation of sugar alcohols^79^ yielding an alkoxide. These anionic species are more reactive compared to their protonated counterparts. The pH at which these anionic species are formed is dependent on the p*K*_a_ of the reactant.^78^ When the pH is increased above 12, a further increase in electrocatalytic activity (current density) for the oxidation of sugar alcohols and saccharides is typically observed (e.g., for electrocatalysts based on Ni, Pd, PtAu, and NiPd).^81−85^ At highly alkaline conditions (3 M NaOH, pH ≥ 14.5), a stagnation or a decrease in activity toward glycerol and glucose oxidation has been reported.^81,83,85^ It has been suggested that the adsorption of hydroxide ions under these conditions prevents the adsorption of the reactants on the surface of the catalyst.^83,85^ However, it is important to note that if hydroxide binds to the surface as neutral hydroxyl OH_ads_, which is usually assumed, there cannot be a higher pH-dependent OH_ads_ coverage at the same potential on the RHE scale.

Depending on the pH, protons or hydroxide ions can act as homogeneous catalysts and thereby induce non-electrochemical conversion of the reactants by isomerization, retro-aldol reaction, aldol condensation, dehydration, Cannizzaro rearrangement, oxidative degradation, and aerobic oxidation reactions. These homogeneous reactions are also affected by the presence of oxygen in solution, temperature, initial reactant concentration, and type of electrolyte.^54,86,87^ Most electrochemical reactions are performed below 60 °C and under anaerobic reaction conditions. At these temperatures, under acidic conditions, most homogeneous reactions do not occur at a significant rate.^88,89^ Therefore, only the homogeneous reactions that prevail under alkaline conditions are discussed here. Moreover, the effect of oxygen present in the solution, which can be formed at the anode surface or can be present in the case of incomplete deaeration before electrocatalytic experiments, must be considered as it strongly influences the products that are formed in homogeneous reactions.^54,87^

Considering isomerization reactions first, glucose tends to isomerize into the thermodynamically more stable fructose or mannose, as illustrated in Scheme 1.^90^ The isomerization rate of glucose is higher at more alkaline conditions (becoming significant at pH ≥ 10.0) and elevated temperatures^28,91,92^ but is also highly dependent on the type of electrolyte, as a combination of bromide and lithium ions can catalyze isomerization reactions in solution.^93^ In line with these observations, the saccharides obtained through the electrocatalytic oxidation of sugar alcohols (e.g., glucose from sorbitol and glyceraldehyde from glycerol) can undergo these non-electrochemical isomerization reactions in alkaline media. Hence, the electrocatalytically formed glyceraldehyde (GALD) can isomerize non-electrochemically to the thermodynamically more stable dihydroxyacetone (DHA) under inert conditions,^94^ while this reaction appears to be limited in oxygen-rich solutions.^54,87^ Therefore, the observation of a high DHA selectivity from glycerol oxidation in alkaline media is not necessarily the result of a selective catalyst; the influence of the solution-phase isomerization must be considered carefully.

**Scheme 2:**
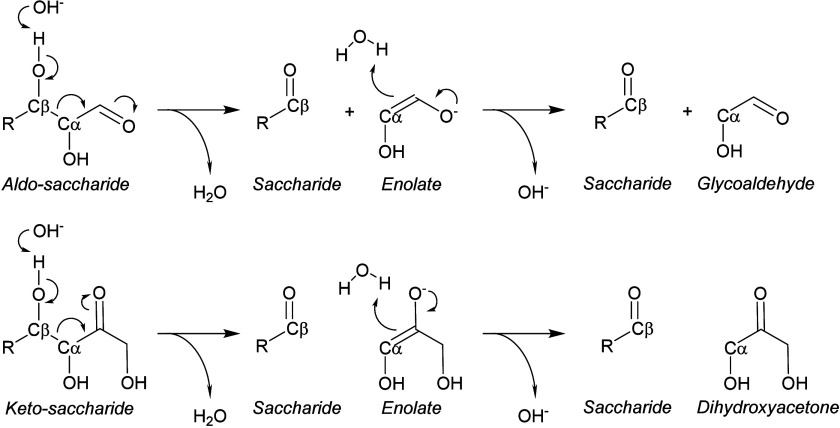
Reaction Scheme Displaying the Retro-Aldolization of Aldo-Saccharides (e.g., Glucose) and Keto-Saccharides (e.g., Fructose) Taking Place in the Electrolyte in the Presence of a Base

An important second class of non-electrochemical homogeneous reactions are retro-aldol reactions, which result in the cleavage of the C_α_–C_β_ bonds adjacent to a carbonyl of keto-saccharides or an aldehyde of aldo-saccharides in alkaline solutions already at room temperature, as illustrated in Scheme 2.^86,87,95^ More specifically, a hydroxide ion abstracts a hydrogen from the C_β_-OH group. As a result, the electrons rearrange and the C_α_–C_β_ bond breaks, resulting in the formation of two smaller molecules. Thus, the retro-aldolization of glucose (i.e., an aldo-saccharide) cleaves the C2–C3 (e.g., C_α_–C_β_) bond resulting in the formation of glycol aldehyde and erythrose, while the retro-aldolization of fructose (i.e., a keto-saccharide) cleaves the C3–C4 bond resulting in the formation of glyceraldehyde and dihydroxyacetone.^86,87^ The successive retro-aldolization of erythrose results in the formation of two glycoaldehydes.^86,95^ Interestingly, the retro-aldolization of glyceraldehyde was not observed under anaerobic conditions,^54^ indicative that this reaction only proceeds for ≥ C4 molecules. Importantly, the retro-aldol reaction is more dominant under anaerobic conditions, as oxygen-rich conditions tend to enhance oxidative C–C cleavage reactions and the aerobic oxidation of aldehydes.^87^ The retro-aldol reaction is in equilibrium with the aldol condensation reaction. For example, in the case of GALD, a proton is abstracted from a hydroxide ion at C_α_, resulting in an enediol which can react with GALD or DHA (isomers) forming glucose and fructose, respectively.

**Scheme 3:**
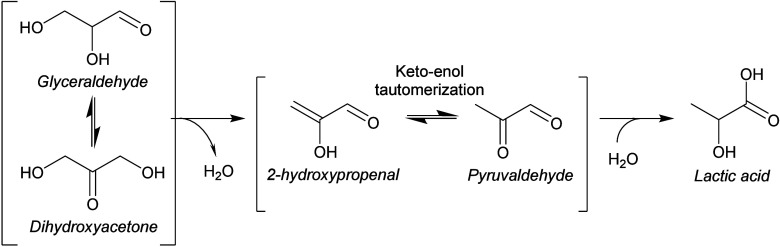
Reaction Scheme Displaying the Different Steps Involved in the Rearrangement of Glyceraldehyde and Dihydroxyacetone to Form Lactic Acid

A third class of relevant non-electrochemical homogeneous reactions is the dehydration of saccharides and their corresponding isomers. Under alkaline conditions, the dehydration of glyceraldehyde or dihydroxyacetone is often reported as it is a key step in the synthesis of lactic acid,^87^ while the dehydration of glucose or fructose has been reported only under more acidic conditions at elevated temperatures,^87,96,97^ since alkaline conditions promote retro-aldol reactions.^87,98^ In the case of GALD and DHA, both molecules can undergo a dehydration to form 2-hydroxypropenal, which can undergo keto–enolic tautomerization to pyruvaldehyde (Scheme 3).^42,87,99^ Successively, pyruvaldehyde can undergo an intramolecular Cannizzaro rearrangement to form lactic acid (LA).^87,99^ These reactions compete with aldol condensation reactions (especially at high glyceraldehyde concentrations^54^) and can be promoted under optimized reaction conditions, resulting in relatively high LA selectivities.^87^ For example, the presence of divalent cations (e.g., Ba^2+^, Cu^2+^, Ca^2+^, Zn^2+^, and Pb^2+^) can redirect the pyruvaldehyde reaction toward a 1,2-hydride shift, resulting in a stabilized lactate salt, which can be acidified to produce LA.^87^ Li et al. showed that the dehydration of DHA to pyruvaldehyde followed by a 1,2-hydride shift is the main reaction pathway for LA formation, while the pathway from GALD to LA only plays a minor role since the LA yield was higher when starting from DHA than from GALD.^87^ Moreover, under oxygen-rich conditions the formation of LA was significantly lower,^54,87^ which was attributed to the promotion of oxidative C–C cleavage reactions and the aerobic oxidation of aldehydes under these conditions.^87^

**Scheme 4:**
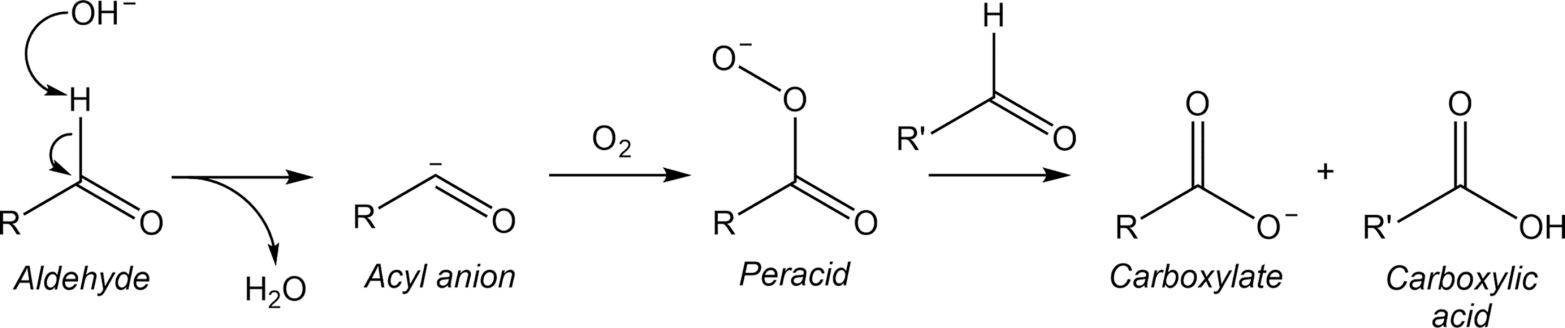
Reaction Scheme Displaying the Oxidation of the Carbonyl Group of an Aldehyde in the Presence of Molecular Oxygen under Alkaline Conditions

**Scheme 5:**
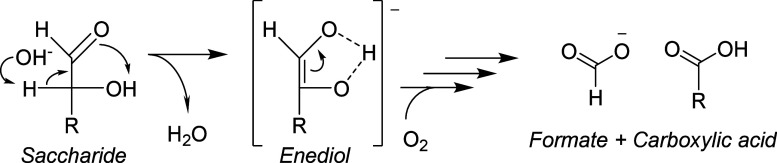
Reaction Scheme Displaying the Oxidative C–C Cleavage of a Saccharide in the Presence of Molecular Oxygen under Alkaline Conditions

A fourth and fifth class of non-electrochemical homogeneous reactions are the oxidation of an aldehydic group to a carboxylic group (Scheme 4) and the oxidative C–C cleavage reactions of saccharides (Scheme 5), respectively. These reactions become dominant in alkaline solutions when oxygen is present in the electrolyte.^54,87^ For example, in the presence of oxygen and at pH = 13, Kwon et al. showed that glyceraldehyde is predominantly converted to glycerate, glycolate, and formate.^54^ In this case, the formation of glycerate is promoted by the oxidation of aldehydes mediated by molecular oxygen. The exact mechanism is not fully established and may vary between the nucleophile–electrophile interaction presented in Scheme 4 and a radical-driven pathway. In parallel, the formation of formate and glycolate is mediated by the oxidative C–C cleavage reactions in solution, as illustrated in Scheme 5. In this reaction, glyceraldehyde first forms an enediol. Successively, the enediol can be attacked by the nucleophilic oxygen molecule at the C1 or C2 group to produce a hydroperoxide intermediate.^100^ The resulting hydroperoxide intermediate undergoes a series of rearrangements, resulting in the formation of formic acid and glycolic acid. The mechanism through which this proceeds remains a topic of debate. In the case of glucose, this reaction proceeds in a similar manner and results in the formation of formic acid and arabinonic acid.^100^

#### 2.1.2 Effect of Electrolyte Ions

**Figure 2:**
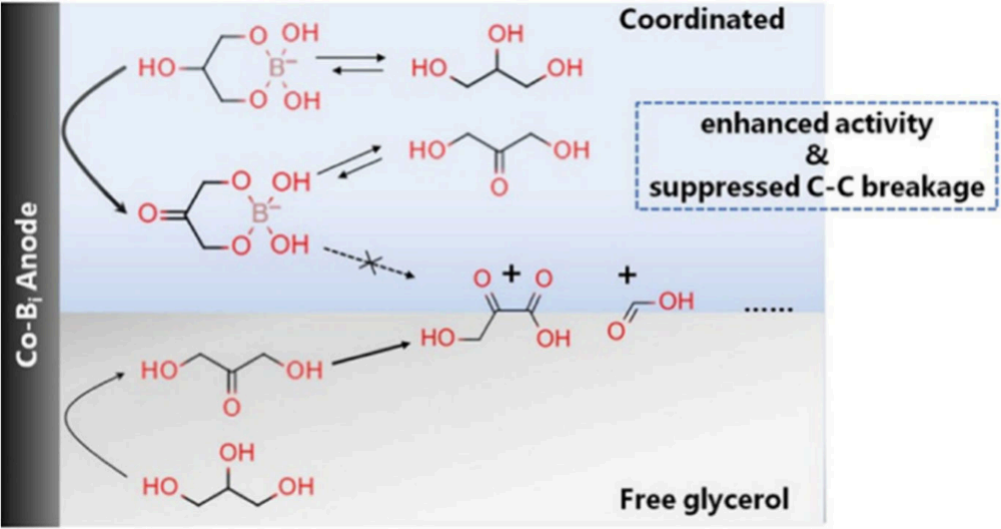
Coordination of glycerol induced by borate ions in solution. By bonding to both primary alcohol groups, the secondary alcohol becomes more susceptible for the electrocatalytic oxidation promoting the formation of dihydroxyacetone and stabilizing dihydroxyacetone through the formation of a dihydroxyacetoneborate complex, hampering further oxidation reactions. Reprinted from ref (102). Copyright 2021 American Chemical Society.

A less commonly studied topic is the effect of electrolyte ions on the catalyst selectivity.^101^ Both the cations that are present in basic electrolyte solutions (e.g., Na^+^, K^+^) and the anions present in acidic electrolyte solutions (e.g., SO_4_^2–^, HPO_4_^2–^, ClO_4_^–^) can influence the electrocatalytic oxidation reactions. In a recent study on the electrocatalytic oxidation of glycerol in basic medium over NiOOH, the effect of electrolyte cations on the electrocatalyst selectivity was researched experimentally and computationally.^101^ It was found that the aldehyde intermediates of the electrocatalytic oxidation of glycerol (glyceraldehyde and glycolaldehyde) were stabilized more effectively in the presence of smaller cations (Li^+^) than in the presence of larger cations (K^+^). As a result, Li^+^ inhibits the successive oxidation of the aldehydes, as was shown by chronoamperometric (CA) measurements. It was argued that the delayed oxidation of these aldehydes gives hydroxide anions more time to induce nucleophilic attacks, thereby promoting C–C cleavage reactions. This effect was substantiated by using crown ethers to coordinate the ions, thus preventing them from interacting with the aldehyde intermediates during the electrocatalytic oxidation of glycerol, which ultimately resulted in a decrease in the rate of C–C cleavage reactions. A related study was conducted with a cobalt borate electrode to electrocatalytically oxidize glycerol with dissolved borax (Na_2_B_4_O_7_) as a supporting electrolyte.^102^ The borax ions hydrolyze to form B(OH)_4_^–^, which were found to interact with the primary alcohol groups of glycerol (as revealed by NMR), consequently promoting the coordination of the secondary alcohol group with the surface of the electrode (Figure 2). This was suggested to enhance the electrocatalytic oxidation of the secondary alcohol group of glycerol, resulting in a high selectivity toward dihydroxyacetone, which could be further promoted from 50 to 60% by increasing the electrolyte concentration from 0.05 to 0.2 M borax.^102^ Likewise, an increase in pH promoted the formation of glycerol-borate complexes, thereby further improving the selectivity toward DHA.^102^ Moreover, for the electrocatalytic oxidation of glycerol in 0.2 M Na_2_B_4_O_7_, a ∼250 mV lower potential resulted in the same current density (e.g., catalytic activity) when compared to 0.2 M Na_2_SO_4_.

The inorganic anions that are present in neutral or acidic electrolytes are also known to influence the electrocatalytic oxidation reactions.^103^ These anions can adsorb on the active sites of the electrocatalyst, blocking them and thus negatively affecting the activity. Different anions typically display different strength of adsorption on the electrocatalyst surface, thus influencing the activity and the selectivity to a different extent.^68^ For example, Melle et al. showed that the activity of Pt for the electrocatalytic oxidation of glycerol decreased in the order HClO_4_ < H_2_SO_4_ < H_3_PO_4_ and attributed the differences in electrocatalytic activity and selectivity to competitive adsorption phenomena.^104^

In conclusion, alkaline conditions promote electrocatalytic reactions by the formation of more electroactive species but can also induce numerous non-electrochemical reactions. These non-electrochemical homogeneous reactions can be coupled with electrocatalytic reactions to steer the selectivity of the system toward a desired product. Since most electrochemical studies on the electrocatalytic oxidation of sugar alcohols^54,57,67,105−108^ and saccharides^28,60,70,78^ are performed under alkaline conditions, we argue that more control experiments on the reactants and the (intermediate) products should be performed to evaluate the effect of non-electrochemical reactions on the obtained product distribution, including the effect of oxygen in solution. This will give more insight on the relation between the electrocatalyst properties and the obtained selectivity and will aid in gaining insights into how the reaction pathways can be controlled. Moreover, the effect of ions present in the electrolyte should not be overlooked as they can form complexes with the reactants or (intermediate) products or adsorb on the electrocatalyst surface and thereby affect the activity and selectivity. This will depend on the intrinsic properties of the ions, such as size, charge, and polarizability.

### 2.2 Effect of Reaction Temperature

The number of publications on the effect of temperature on the electrocatalytic oxidation of sugar alcohols and saccharides is limited. Yet, the temperature becomes an increasingly important factor for large scale electrocatalytic systems, since these systems suffer more significantly from heat generated by resistances (e.g., ohmic drop). Therefore, research on the electrocatalytic oxidation of sugar alcohols and saccharides should dedicate more attention to the effect of temperature. The role of temperature on the performance of Au and Pt electrocatalysts toward the oxidation of sugar alcohols and saccharides was found to be independent of the functional groups of the reactant itself (C–OH vs C=O). It is worth highlighting that almost all the publications in which the influence of temperature was studied were performed under alkaline conditions^28,109−117^ with, to the best of our knowledge, only one paper studying temperature effects under acidic conditions.^118^

**Figure 3:**
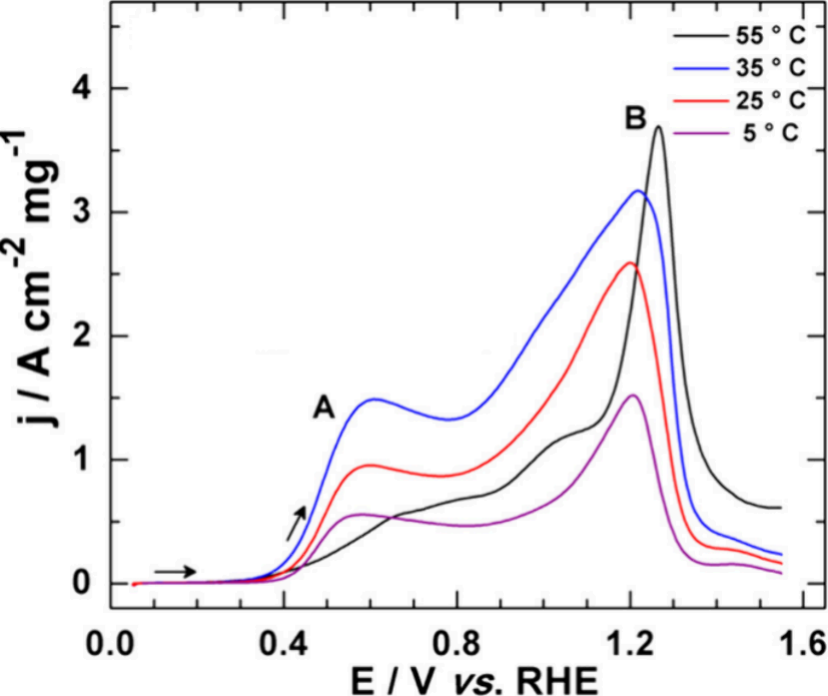
LSV of an Au-based electrode in 0.1 M NaOH at 20 mV s^–1^ scan rate in the presence of 10 mmol L^–1^ glucose at different temperatures in the 5–55 °C range. Adapted from ref (115). Copyright 2015 American Chemical Society.

Most studies that report an effect of temperature on the electrocatalytic oxidation of sugar alcohols and saccharides show that an increase in temperature results in an expected increase in current density, as measured by LSV (linear-sweep voltammetry) or cyclic voltammetry (CV).^109,110,112−117^ In fact, several researchers have shown that there is an approximate linear relationship between the natural logarithm of the peak current density and the inverse of the temperature for the oxidation of both sugar alcohols (glycerol and sorbitol)^110,116^ and saccharides (glucose),^115,117^ which is in line with the Arrhenius equation. Likewise, for Pt/C at pH = 8, with increasing temperatures from 20 to 50 °C, the cell potential decreased and the glucose oxidation reaction rate increased.^119^ By contrast, for Au-based electrocatalysts (at *E* = 0.4–1.3 V) in the presence of glucose and under alkaline conditions (pH = 13), when increasing the temperature from 35 to 55 °C the catalyst activity dropped (Figure 3).^115^ This effect was attributed to the formation of poisoning species, as indicated by the coloration of the electrolyte solution.

The reaction temperature can have a negative effect on the selectivity, as was shown for the electrochemical oxidation of glycerol over Pt/C and PtRu/C electrocatalysts in terms of the production of tartronic acid (TA).^112,113^ In this system, the selectivity toward C3 oxidation products was reduced from 96 to 91% and down to no more than 7% when the temperature was increased respectively from 30 to 60 to 90 °C, as C–C cleavage reactions were boosted at high temperature. Similarly, for the electrocatalytic oxidation of glucose to gluconic acid (GA) over Au-based electrodes, high temperatures resulted in higher concentrations of fructose (isomerization) and C–C cleavage products (degradation).^28^ The C–C cleavage products prevail at high temperatures, as higher activation energy barriers are more easily surpassed. Under alkaline conditions (pH > 10), an increase in temperature can induce two effects in the conversion of glucose. First, the reactant concentration can decrease due to non-electrochemical reactions such as the isomerization of glucose (mainly to fructose) and retro-aldol reactions forming C1–C3 products. Second, an increase in the concentration of these products can induce a lower catalytic activity as they are less reactive toward oxidation, e.g., in the case of fructose when compared to glucose.^109^

Some studies also report a positive temperature effect on the electrocatalyst selectivity, as was shown for the production of gluconic acid and glucaric acid from glucose and dihydroxyacetone or lactic acid (LA) from glycerol.^27,111,118,120^ For instance, under acidic conditions (0.5 M H_2_SO_4_), a selectivity of 63% dihydroxyacetone was achieved at 60 °C with PtSb/C,^118^ being the highest selectivity in the temperature range of 25–70 °C. Lam et al. achieved 34% LA selectivity with a Co-based electrocatalyst at 60 °C,^111^ while the selectivity toward LA was only 16% if the process was carried out at 40 °C. The enhanced selectivity toward LA at higher temperature can be attributed to non-electrochemical reactions (section 2.1.1, see discussion on dehydrogenation and Cannizzaro rearrangement). Other studies show that high temperatures decrease the selectivity for the electrocatalytic oxidation of sugar alcohols and saccharides.^28,112,113^ For the oxidation of glucose under neutral conditions, it was shown that glucaric acid (selectivity = 65%) is best achieved at 15 °C, while at 30 °C the highest selectivity was obtained toward gluconic acid, whereas even higher temperatures resulted in a loss in selectivity toward glucaric acid and gluconic acid.^27^ A possible explanation could be the use of acidic conditions in this study, which limit isomerization and C–C cleavage reactions.^111^

In summary, there is only limited research on the effect of temperature in electrosynthesis studies for sugar alcohols and saccharides oxidation. Yet, large scale electrocatalytic systems suffer not only from overpotentials but also ohmic drop, leading to heating effects, thereby affecting the temperature of the system. Hence, it is crucial to gain more insight on the effect of temperature on electrocatalytic systems. The studies that have been devoted to the effect of temperature are often performed under alkaline conditions, which are likely to be severely affected by (temperature-dependent) non-electrochemical reactions (see section 2.1). This issue should be addressed more carefully in future studies.

### 2.3 Effect of Reactant Concentration

There have been several studies on the electrocatalytic oxidation of monosaccharides and sugar alcohols in which the influence of the reactant concentration was assessed.^28,56,71,82,110,121−127^ Some trends can be identified by comparing the results of various systems, irrespective of the type of reactant.

**Figure 4:**
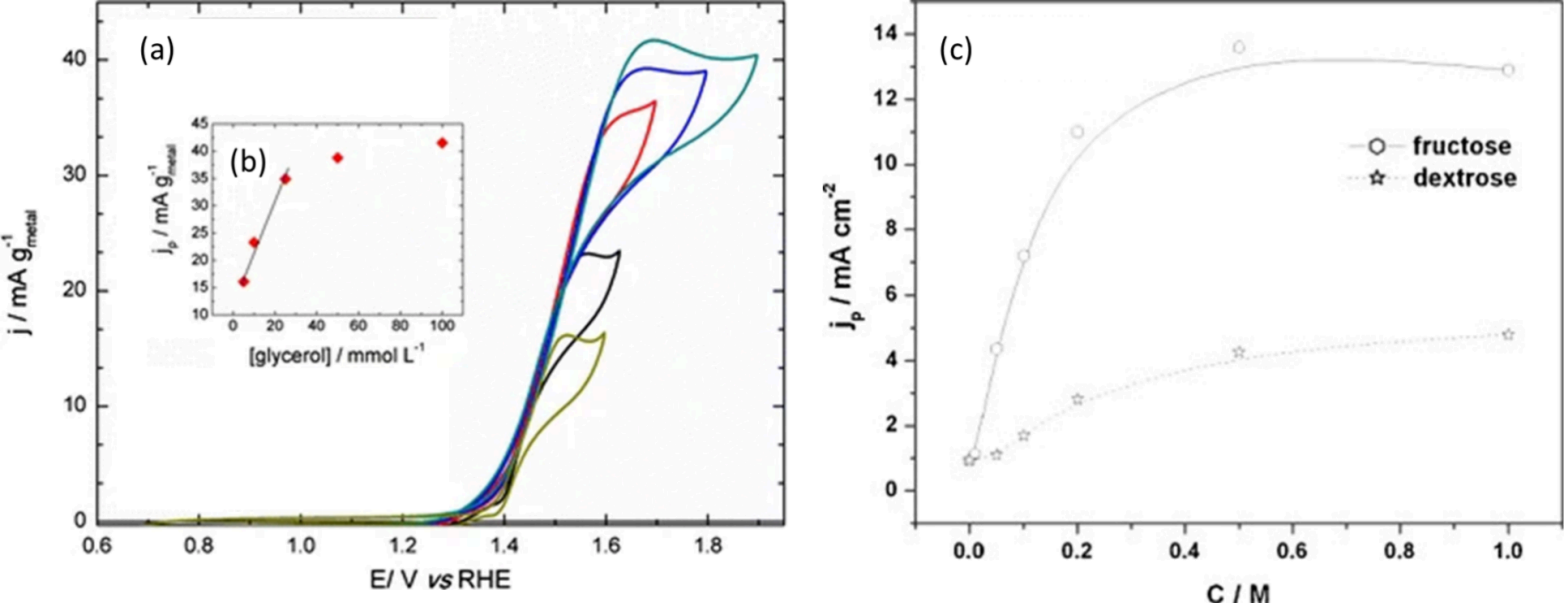
(a) CV of a Ni/C electrode in 0.1 M NaOH at different glycerol concentrations and (b) the influence of glycerol concentration on the anodic peak current density, as derived from plot (a). Adapted with permission from ref (82). Copyright 2015 Springer Nature. (c) Peak current density for fructose and glucose (dextrose) oxidation on a MnO_2_/Pt electrode in 0.5 M Na_2_SO_4_, as a function of reactant concentration. Adapted with permission from ref (122). Copyright 2008 Elsevier.

If we consider the example of the oxidation of glycerol in alkaline environment (Figure 4a), in which the reaction is carried out with a large excess of base compared to glycerol (as it generally is the case), an approximately first-order reaction kinetics in reactant concentration is observed up to a certain concentration, above which the reaction order decreases. Such a behavior has been reported for the electrocatalytic oxidation of both monosaccharides and sugar alcohols, showing an initially linear increase in current density in cyclic voltammetry (CV) experiments with increasing reactant concentration (Figure 4b shows an example for fructose and glucose, from a neutral solution).^71,82,110,121−124^ At lower concentrations, the diffusion rate of the reactant from the bulk to the electrode (which depends on the reactant concentration) is lower than the conversion rate of the reactant at the electrode, implying that all the reactant that reaches the electrocatalyst surface is immediately converted, and first-order kinetics are observed. At higher concentrations, the conversion rate of the reactant at the electrode becomes the limiting factor, implying that the active sites of the catalyst are saturated with the adsorbed reactant, and zeroth-order kinetics are observed in the CV tests (Figure 4b, c).^82,122,123,125,126^ It should be noted that very high concentration of saccharides and sugar alcohols can be detrimental as it can lead to an increased viscosity of the solution, which can decrease the conductivity of the electrolyte and thus result in higher ohmic losses. Moreover, the high reactant concentrations can result in an excess of (oxidized) compounds on the electrocatalyst surface, hindering the adsorption of hydroxide ions and thus decreasing the oxidation rate of the adsorbed reactants.^119^

At similar reaction times in batch cells^28,56,127^ and at similar hydraulic retention times in flow cells^128^ but different initial reactant concentrations, the selectivity can significantly differ as different reactions may have different reaction orders. For example, low reactant concentrations tend to lead to high conversions in chronoamperometry tests (i.e., longer tests than those by CV), resulting in the formation of primary oxidation products (e.g., glyceraldehyde from glycerol and gluconic acid from glucose) together with further oxidation products (e.g., glyceric acid in the case of glycerol and glucaric acid in the case of glucose).^28,56,127,128^ For higher reactant concentrations, the conversion decreases, together with a rise in the selectivity toward primary oxidation products: the reactant tends to occupy the electrocatalyst surface, thereby decreasing the formation of sequential oxidation products.^28,56,127^ For the electrochemical oxidation of glycerol in 0.1 M KOH on AuPt bimetallic catalysts during 12 h,^81^ the overall conversion decreased from 58 to 15% when the glycerol concentration was increased from 0.1 to 1 M, but the selectivity toward lactic acid remained unaltered. A possible explanation is that the initial oxidation product of glycerol, glyceraldehyde, is converted non-electrochemically in the basic solution into lactic acid (see section 2.1.1).

To conclude, the initial reactant concentration can strongly influence the reaction rate and the reaction selectivity. Studies showed that an increase in reactant concentration changes the kinetics from quasi-first order, dominated by diffusion rates of the reactant to the surface of the catalyst, to zeroth order, dominated by intrinsic kinetic rates of the catalyst. Moreover, considering similar reaction times, low initial reactant concentrations result in multiple oxidation products, while high initial reactant concentrations result in fewer oxidation products, resulting in less complex reaction mixtures and thus easier downstream processing procedures. Hence, to compare the performance of catalysts between different studies it is recommended to use similar reactions conditions, such as initial reactant concentrations, pH, temperature, reaction times, and number of catalytic active sites.

## 3 Potential-Dependent State of the Surface of Metal-Based Electrodes

In electrocatalytic oxidation processes, the kinetics and selectivity of the reaction can be controlled by means of the applied potential. Yet, an increase in applied potential can have two effects: (1) it lowers the activation energy for electron transfer reactions and thereby increases the rate of electrocatalytic oxidation, and (2) it can change the oxidation state of the electrocatalyst surface from metallic to oxidic (i.e., metal hydroxide, oxyhydroxide, or oxide), which in turn can affect the activity and selectivity of the electrocatalyst. Therefore, in this section we distinguish between these two effects by considering the surface oxidation state of the electrocatalyst as a function of potential, with particular focus on Au and Pt electrodes.

**Figure 5:**
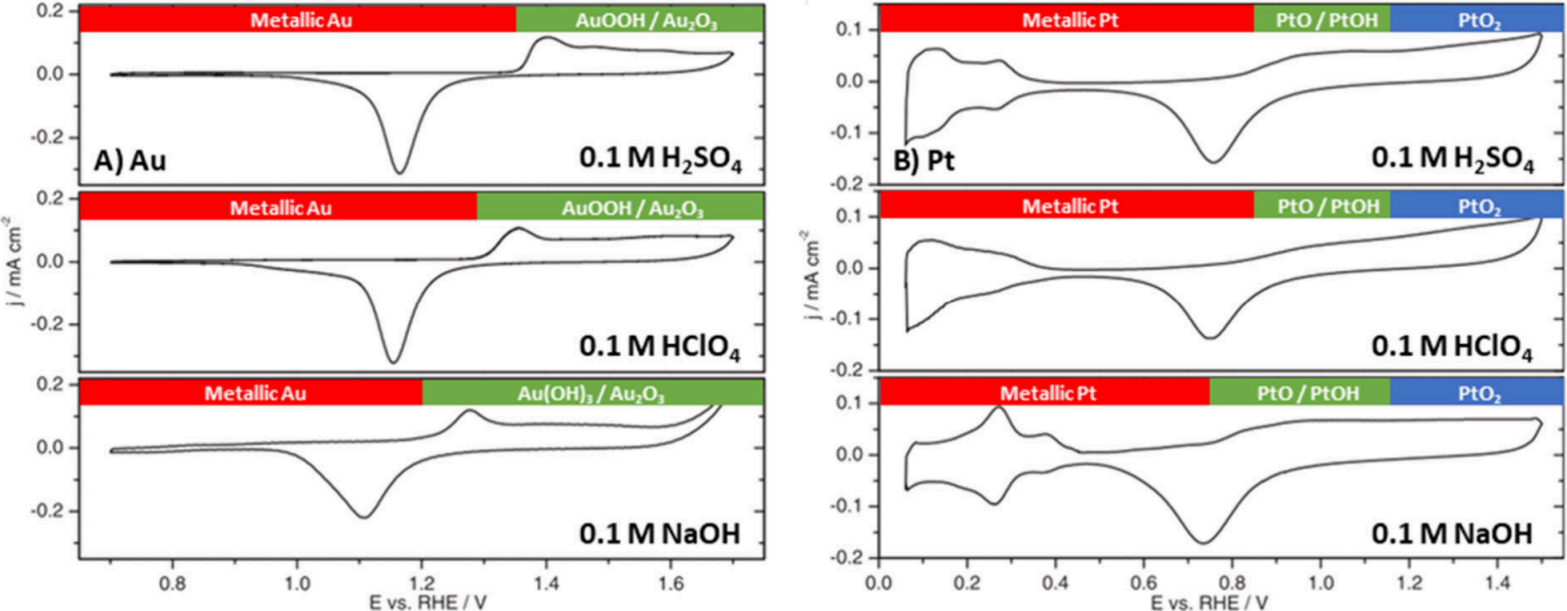
Cyclic voltammograms of (A) polycrystalline Au and (B) polycrystalline Pt, obtained under acidic conditions (0.1 M H_2_SO_4_ and 0.1 M HClO_4_) and alkaline conditions (0.05 M NaOH), measured in a flow cell at 50 mV s^–1^. Adapted with permission from ref (129). Copyright 2014 The Electrochemical Society.

A common approach to evaluate the oxidation state of electrodes is by deriving it from the Pourbaix diagrams. However, these diagrams do not only consider the surface oxidation state but also the bulk oxidation state. Therefore, to illustrate the effect of the potential on the state of the electrode surface, we present in Figure 5 the so-called blank cyclic voltammograms of polycrystalline Au and Pt in three representative electrolytes.

For Au in 0.1 M H_2_SO_4_, an increase in current density can be observed at 1.35 V vs RHE, which corresponds to the formation of gold oxyhydroxide/gold oxide (AuOOH/Au_2_O_3_), as was shown by in situ surface-enhanced Raman spectroscopy (SERS).^130^ In 0.1 M HClO_4_, the onset potential for the formation of AuOOH/Au_2_O_3_ is ∼1.28 V, based on DFT calculations^131^ and in situ SERS,^132^ which is 0.07 V lower than in 0.1 M H_2_SO_4_ (as also deduced from the different onset potential for surface oxidation in Figure 5A). By contrast, at pH = 12.7 (0.05 M NaOH) an increase in current density already takes place at 1.2 V vs RHE and results in the formation of a gold hydroxide/gold oxide (Au(OH)_3_/Au_2_O_3_) species.^130^ This shows that metallic Au has a larger potential window under acidic conditions than under alkaline conditions^129,130^ and that in acidic media anions strongly influence the surface oxidation potential.^129,130,132^ The presence of Au_2_O_3_ at potential above 1.70 V under acidic conditions (0.1 M HClO_4_) and above 1.57 V under alkaline conditions (0.05 M NaOH) was shown by *in situ* electrochemical surface-enhanced Raman spectroscopy.^132^ The surface gold oxides reduce again with decreasing potential conditions at 1.18 V in acidic conditions and at 1.1 V in alkaline conditions.^129,130^ The difference in reduction peak potential under acidic and alkaline conditions is an indication of the formation of different surface oxide species under these conditions.^129,130^

The typical CVs of Pt at different pH conditions are illustrated in Figure 5B. For Pt at pH = 1 and pH = 13, the positive current from 0.1 to 0.35 V vs RHE corresponds primarily to the desorption of adsorbed hydrogen.^133−135^ At step edges and corner sites, the desorbed hydrogen is replaced by adsorbed *OH species under both acidic and alkaline conditions in this potential region.^133−136^ At pH = 1, for systems containing H_2_SO_4_ or HClO_4_, an onset potential for surface oxidation can be observed at 0.85 V vs RHE, which is related to the chemisorption of hydroxide and/or oxygen on the Pt surface (PtO/PtOH_surf_),^129,137−139^ while at pH = 12.7 the onset potential for PtO/PtOH_surf_ formation can be observed at 0.75 V vs RHE.^129,134^ This shows that the potential window for metallic Pt is larger under acidic conditions than under alkaline conditions. Independent of the pH, above 1.15 V the place exchange of oxygen and platinum atoms occurs, and oxygen penetrates the Pt lattice up to a few monolayers to form PtO.^133,134,140,141^ At 1.2–1.3 V vs RHE, PtO is converted into PtO_2_, and when the potential is decreased, PtO_*x*_ reduces back to metallic Pt at ∼0.75 V under both acidic and alkaline conditions.^133,134,137,142,143^ We note that the exact surface composition of the surface oxide is mixed and very dependent on the structure of the underlying metallic Pt. Our usage of the terms PtO/PtOH_surf_, PtO, and PtO_2_ is therefore indicative of the expected dominant surface oxide but should not be taken as an accurate indication of a single Pt surface oxidation state.

The above discussion shows that the surface oxidation state of Au and Pt depends on the applied potential and pH of the electrolyte. For Au, the following surface oxidation states can be distinguished: (1) metallic Au from 0.1 to 1.28 V vs RHE under acidic conditions,^131,132^ from 0.1 to 1.20 V vs RHE under alkaline conditions^130^ and (2) AuOOH/Au_2_O_3_ above 1.28 V vs RHE under acidic conditions,^131,132^ and (3) Au(OH)_3_/Au_2_O_3_ above 1.20 V vs RHE under alkaline conditions.^130,132^ For Pt, the following surface oxidation states can be distinguished: (1) metallic Pt from 0.1 to 0.85 V vs RHE under acidic conditions^129,137,138^ and from 0.1 to 0.75 V vs RHE under alkaline conditions,^129,134^ (2) PtO with possible surface hydroxide groups exists from 0.85 to 1.3 V vs RHE under acidic conditions^129,137,138^ and from 0.75 to 1.3 V vs RHE under alkaline conditions,^129,133,134,140,141^ and (3) PtO_2_ at > 1.3 V vs RHE.^133,134,137,142,143^ As mentioned, in reality mixed oxidic phases are expected to exist on the surface of the electrode.

When the electrode is in the metallic state, the oxidation of biobased reactants occurs through a direct pathway, i.e., a mechanism in which the oxidation of the reactant is driven by the applied potential, and the larger the potential, the higher the reaction rate (potential-dependent mechanism). On oxidized metal surfaces, besides the direct pathway, the oxidation may also proceed through an indirect pathway. In the indirect mechanism, the reactant is chemically oxidized by metal species that are in a high oxidation state: the metal gets thus chemically reduced in the redox process, after which it is electrochemically reoxidized to its original high oxidation state. Examples of the two pathways are given in sections 4 and 5.

In this review, all the reference potentials found in the literature have been converted to RHE to avoid potential shifts caused by the pH of the electrolyte.^144^ Therefore, the potentials reported in this paper (which are referred to RHE) may deviate from potentials in the cited articles (which are typically referred to Ag/AgCl or Hg/HgO). These calculated RHE potentials can then be used to estimate the oxidation state of the Au and Pt catalyst under acidic and alkaline conditions, based on the cyclic voltammetry shown in Figure 5. This approach enables us to group studies that have been conducted under similar reaction conditions and define trends for the electrocatalytic oxidation of sugar alcohols and saccharides. Yet, it must be noted that the actual surface oxidation state of Pt and Au is often not precisely known and that this approach only gives a first approximation. Other factors that might affect the surface oxidation state of Au and Pt are the type of electrolyte, the pH of the electrolyte, the adsorption of reactant/products, and (if present) the support of the metal species in the electrocatalyst. For example, a different electrolyte, such as sulfate or perchlorate, slightly changes the onset of oxidation of Au and Pt.^145^ In addition, the pH affects the type of surface oxide formed,^130^ while the support can either withdraw or donate electrons to the supported metal,^146,147^ thereby affecting the oxidation state of the electrocatalyst. Therefore, we will consider these factors when there is evidence that they had a significant impact on catalyst performance.

## 4 Effect of Electrocatalyst Properties under Various Reaction Conditions on the Oxidation of Sugar Alcohols

This section presents and discusses the trends reported for the electrocatalyst activity and selectivity in the electrochemical oxidation of sugar alcohols. In the majority of the studies that address this topic, Au and Pt (sections 4.1.1 and 4.1.2, respectively) were employed as electrocatalysts, for which it has been possible to define trends with respect to the electrocatalyst properties such as the type of metal used, the oxidation state of the metal, and the type of bimetallic catalyst. Au is discussed first as it is generally found to be a less active but more selective electrocatalyst for oxidation reactions when compared to Pt. Studies with other metals are scarcer, but some of them allow achieving high selectivity toward specific value-added products (see section 4.1.3–4.1.5). Before discussing in detail the trends observed in the literature, the main mechanistic pathway for the electrocatalytic oxidation of glycerol is summarized.

**Scheme 6:**
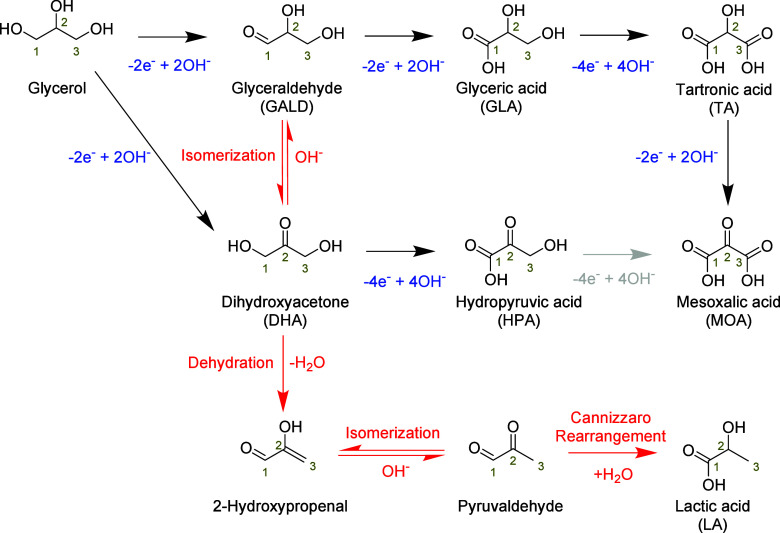
Main Reaction Pathways for the Electrocatalytic Oxidation (Black Arrows with Number of Electrons/Hydroxides in Blue), Potential but Non-Observed Electrocatalytic Oxidation Pathway (Grey Arrow), and Non-Electrochemical Conversion (Red Arrows) of Glycerol and Derivatives Observed in the Literature

The most commonly studied sugar alcohol in electrocatalytic oxidation reactions is glycerol, which we use in this review as a basis to define trends found in the literature. The trends observed with glycerol were compared to the electrocatalytic oxidation of other sugar alcohols. Scheme 6 gives an overview of the different reaction pathways published.^54,81^ The scheme shows that glycerol can either be electrocatalytically oxidized at the primary or secondary alcohol group, forming respectively glyceraldehyde (GALD) or dihydroxyacetone (DHA). GALD and DHA interisomerize in the electrolyte under alkaline conditions, with DHA being the more stable isomer (see section 2.1.1). Under oxidative potentials, the aldehyde group of GALD can be electrochemically oxidized to form glyceric acid (GLA), which in turn can be further oxidized at the remaining primary alcohol group to form tartronic acid (TA). TA can also be oxidized electrochemically at the secondary alcohol to form mesoxalic acid (MOA). DHA can be oxidized electrochemically at the primary alcohol to form hydropyruvic acid (HPA). HPA could potentially be oxidized electrochemically to MOA,^148^ although this pathway has not yet been observed. Alternatively, under alkaline conditions GALD and DHA can be dehydrated non-electrochemically in the electrolyte to form 2-hydroxypropenal, which can undergo keto–enolic tautomerization to pyruvaldehyde (see section 2.1.1). Successively, pyruvaldehyde can undergo an intramolecular Cannizzaro rearrangement (see section 2.1.1) to form lactic acid (LA).

### 4.1 Monometallic Electrocatalysts for the Oxidation of Sugar Alcohols

This section describes the trends for the selective electrocatalytic oxidation of sugar alcohols over the most studied electrocatalysts, being those based on Au (section 4.1.1) and Pt (section 4.1.2), and compares the trends with electrocatalysts based on Pd, Ir, and Ru (section 4.1.3), Ni and Co (section 4.1.4), and Cu and Mn (section 4.1.5). The trends for these electrocatalysts have been categorized based on increasing pH and potential.

#### 4.1.1 Au-Based Electrocatalysts for the Oxidation of Sugar Alcohols

This section discusses the electrocatalytic oxidation of glycerol on Au at different pH and potentials. To our knowledge, only Valter et al. studied the differences in activity for glycerol oxidation on different Au facets.^68^ Moreover, only a few studies have reported the electrocatalytic oxidation of sugar alcohols over Au electrodes under acidic^67,68^ and neutral conditions.^54^ On the other hand, alkaline conditions have been widely studied, as these conditions give higher activity due to the higher reactivity of the alkoxides (see section 2.1.1).^54,57,67,105−108^ Acidic conditions are considered first, to distinguish between electrochemical and non-electrochemical reactions, even though the activity of Au is poor under acidic conditions.

Only three studies report that Au has some activity for the electrocatalytic oxidation of sugar alcohols under acidic conditions,^67,68,149^ while other studies argue that Au has no appreciable activity^79^ or is inactive under these conditions (based on LSV).^54,150^ CV experiments on an Au electrode in 0.1 M HClO_4_ showed that the currents are higher in the presence of glycerol than in its absence, in a broad potential range (at *E* = 0.55–1.65 V). However, current densities are (very) low (< 0.1 mA cm^–2^), with somewhat higher current densities (0.4 mA cm^–2^) obtained at *E* ≥ 1.30 V.^68^ On the basis of the range of potentials in which activity toward glycerol oxidation was observed and considering that Au is expected to be in an oxidized state at *E* ≥ 1.30 V (see section 3), it can be inferred that both metallic and oxidized Au species at the electrode surface are mildly active for glycerol oxidation. By contrast, in 0.1 M H_2_SO_4_, CV experiments showed that in the presence of glycerol a significant current density was achieved only at *E* ≥ 1.30 V,^68^ with an ∼0.1 mA cm^–2^ lower activity than in HClO_4_. This was attributed to the stronger adsorption of the sulfate anions compared to perchlorate anions, thereby blocking active sites.^68^ DFT calculations predicted that the catalytic oxidation of glycerol on Au(111) at the primary and secondary alcohol group to form DHA and 2,3-dihydroxy-2-propenal would occur at 0.39 V,^68,149^ the cleavage of C–C bonds of glycerol to form CO at 0.5 V,^68^ and catalytic oxidation of the primary alcohol group of glycerol to form GALD at 0.6 V,^68,149^ although experimental analysis was not performed to detect these products.^68,149^ On the basis of FTIR experiments on Au-based electrodes in 0.1 M H_2_SO_4_, it was argued that at *E* ≥ 1.20 V Au catalyzes primary alcohol oxidation to produce TA and induces C–C cleavage reactions to form formic acid (FA) and carbon dioxide (CO_2_).^67^

Kwon et al. reported the selectivity of the electrocatalytic oxidation of glycerol over Au electrodes under neutral conditions (0.1 M Na_2_SO_4_).^54^ LSV was combined with online HPLC, showing that between 0.8 and 1.2 V the Au electrode performs the electrocatalytic oxidation of glycerol at the primary alcohol group with >99% selectivity, forming GALD,^54^ similarly to the results suggested by DFT calculations on Au(111).^68^ At *E* > 1.2 V, the formation of CO_2_ was observed by gas chromatography (not quantitatively determined), indicative of C–C cleavage reactions.^54^

**Scheme 7:**
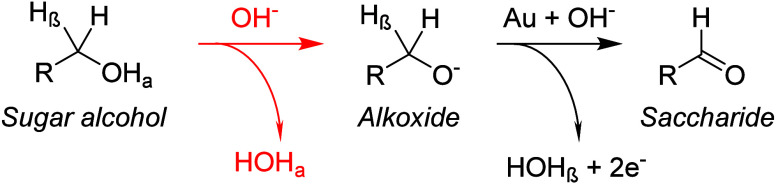
Mechanistic Pathway Described by Kwon et al. for the Electrocatalytic Oxidation of Sugar Alcohols at the Primary Alcohol Group to the Terminal Aldehyde on Au and in Alkaline Conditions, Where the First Step Is Non-Electrochemical (Red Arrows) and the Successive Step Is Electrochemical (Black Arrows) Adapted from ref (79). Copyright 2011 American Chemical Society.

Most studies on the electrocatalytic oxidation of sugar alcohols with Au electrodes were conducted under alkaline conditions.^54,57,67,105−108^ The reason for this is that for Au catalysts, the first deprotonation step of the H_α_ proton of the primary alcohol of a sugar alcohol (H_β_R–OH_α_) is thermodynamically favorable only in alkaline media (base-promoted), while the step involving the abstraction of H_β_ is fast and Au-catalyzed.^79^ The alkaline conditions thereby promote the formation of the alkoxide in solution (see section 2.1.1), which reacts at the Au catalyst surface (Scheme 7). The Au-based electrocatalyst abstracts the H_β_ from the alkoxide and acts as electron acceptor promoting the formation of the aldehyde (saccharide).^79,80^ The high activities achieved under alkaline conditions with Au are therefore attributed to base-promotion and not to the catalyst-hydroxide interaction.^79^ By contrast, DFT calculations do suggest that higher activities are achieved at potentials close to the onset potential (∼0.8 V vs RHE) for hydroxide adsorption on metallic Au.^149^ The DFT calculations indicated that adsorbed OH could lower the barrier for β-elimination,^151^ suggesting that some interaction with (hydroxylated) Au surface is required.^152^ However, it is likely that this adsorbed OH in the DFT calculations plays a similar role to the hydroxide in alkaline solution.

The selectivity for the electrocatalytic oxidation of glycerol on Au-based electrocatalysts was evaluated by LSV combined with online HPLC under alkaline conditions (pH = 13).^54,106^ Under these conditions, significantly higher current densities (up to 20 mA cm^–2^) were achieved compared to the results under acidic and neutral conditions.^54,79,106^ At high potential (*E* = 1.4–1.6 V), the activity of the Au electrode dropped dramatically,^54,79^ due to the formation of Au surface-oxide species (Au_2_O_3_, see section 3) that passivate the surface.^106^ With an Au electrode in basic medium, 20% GLA was formed with a high content of C–C cleavage products, namely 80% glycolic acid and FA. GALD was not detected, which indicates that this compound is quickly oxidized to GLA and successively cleaved at the C–C bond to glycolic acid and FA as a result of the high potentials.^54,106^ Long-term electrolysis of glycerol over Au electrodes at pH = 13 were run at 1.1 and 1.3 V.^108^ After 20 h at *E* = 1.1 V, the conversion of glycerol was 10%, and the main products were FA and glycolic acid (selectivity = 82%) and to a lesser extent GLA (8%) and TA (10%).^108^ At *E* = 1.3 V, the conversion of glycerol was slightly higher (14%) with higher selectivity toward FA and glycolic acid (91%) and 9% GLA. The increase of C–C cleavage products seems to correlate with an increase in applied potential and the alkalinity of the electrolyte. Results obtained by LSV measurements at pH = 13 combined with FTIR were interpreted differently.^67^ This study suggested that the oxidation of glycerol on Au electrode at 0.7 V *< E* < 1.2 V) could also produce DHA, while at higher potentials (*E* > 1.2 V) higher oxidation products were obtained, such as TA, MOA, glycolic acid, and CO_2_.^67^ It is worth noting that the formation of DHA and MOA has not been reported in the other studies conducted under similar conditions.^54,106,108^ Additionally, similar studies conducted with HPLC did not detect the formation of TA.^54,106,108^ This discrepancy could potentially be attributed to the analytical method employed, where TA is an intermediate product that does not desorb from the surface and could therefore not be detected by HPLC but would be identifiable by FTIR. Moreover, the assignment of bands in FTIR spectra to specific species is not unambiguous, creating uncertainty in the products that are being reported.^153,154^ Finally, controversies exist for peak identification caused by convolution of peaks which may mask weak bands or shift the center of peaks.^155^ In general, we consider product assignment based on HPLC more reliable. Therefore, this review will focus primarily on papers that use HPLC for product analysis, even though FTIR has been a main analytical technique applied to the electrocatalytic oxidation of glycerol.^155^

Under extreme alkaline conditions (pH = 14.3–14.9) and high temperatures (*T* = 50–60 °C), 3 nm Au particles supported on carbon (Au/C) promote the electrocatalytic oxidation of the two primary alcohol groups of glycerol producing TA at *E* < 0.9 V, and also promote the electrocatalytic oxidation of the secondary alcohol group of TA to form MOA.^105,107^ This has been observed employing both a batch-electrolysis cell and an anion-exchange-membrane-based direct glycerol cell (AEM-DG cell), where the latter was operated by controlling the cell potential. In an electrolysis cell, at pH = 14.3 and 50 °C, 10% of glycerol was oxidized over an Au/C electrocatalyst (5.0 mg_metal_ cm^–2^) at *E* = 0.5 V to MOA (selectivity = 47%), TA (25%), GLA (14%), and oxalate (14%).^107^ At higher potentials (*E* = 0.9–1.2 V), the formation of C–C cleavage products (glycolate, glyoxylate, and oxalate) was promoted.^107^ At the same pH and in an anion-exchange membrane cell, with an Au/C electrocatalyst (1.0 mg_metal_ cm^–2^) at *E* = 0.3 V cell potential, 10% glycerol was converted to MOA with ∼50% selectivity. In a follow-up study, using the same electrochemical cell, under harsher conditions (pH = 14.9 and 60 °C) and with continuous flow of fresh reactant (1.0 mL min^–1^), the Au/C electrocatalyst (0.3–0.5 V vs RHE) was able to promote the oxidation of 90% glycerol to TA (selectivity = 70%), GLA (15%, primary alcohol oxidation), LA (15%, primary or secondary alcohol oxidation), and traces of MOA (primary and secondary alcohol oxidation) and of oxalate.^105^ The lower selectivity toward MOA and higher selectivity toward TA in this system was attributed to the optimized reaction conditions (flow rate through the electrochemical reactor, temperature, pH, catalyst loading) to prevent the successive oxidation of TA to MOA.^105^ In summary, these studies show that an increase in temperature and pH at lower potentials allows achieving a high degree of oxidation while avoiding C–C cleavage reactions.

In conclusion, Au is barely active under acidic and neutral conditions but can selectively form GALD under neutral conditions from glycerol. By contrast, under alkaline conditions (pH = 13), at room temperature, oxidation of glycerol on Au mainly produces C–C cleavage reactions. In general, the importance of the alkaline medium must be considered for the C–C cleavage reactions, and more clear-cut data are needed to determine to what extent the medium or the electrocatalyst promote these reactions. The overall trend shows that Au is likely only able to catalyze the oxidation of primary groups of sugar alcohols. However, if the alkalinity is increased to very extreme conditions (pH ≥ 14.3) and the temperature is increased to 50–60 °C, while the potential is kept low (*E* ≤ 0.9 V), Au can catalyze with relative high selectivity the formation of TA or MOA, depending on the tuning of the reaction conditions.

#### 4.1.2 Pt-Based Electrocatalysts for the Oxidation of Sugar Alcohols

This section discusses the electrocatalytic oxidation of glycerol on Pt at different pH and potentials. In contrast to gold, platinum is well-known for its good electrocatalytic performance over a broad range of reaction conditions, e.g. from acidic to alkaline pH.^54,67,106,156^ Therefore, Pt is one of the most widely studied metals for the electrocatalytic oxidation of sugar alcohols. In this section, the most relevant trends regarding the performance of Pt toward the electrocatalytic oxidation of glycerol (and other sugar alcohols) are summarized.

**Figure 6:**
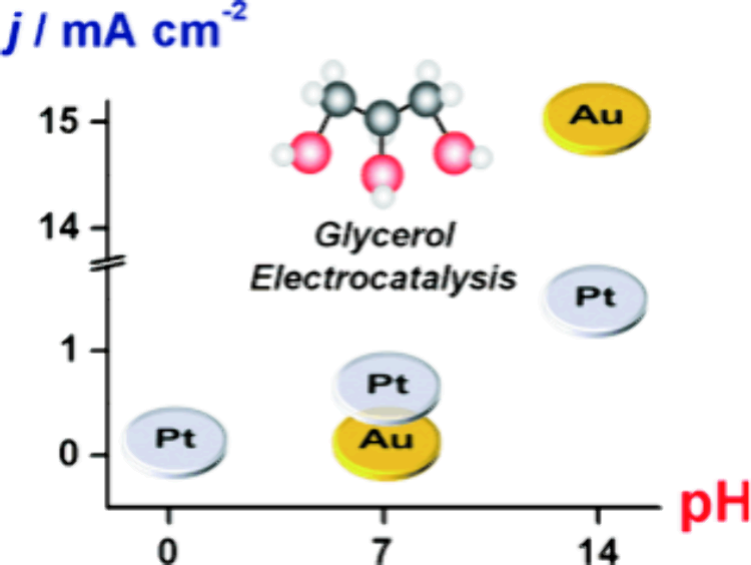
Electrocatalytic activity of Pt and Au toward the oxidation of glycerol as a function of the pH of the electrolyte. Reprinted with permission from ref (54). Copyright 2011 Wiley-VCH.

Figure 6 shows the relation between the activity of Pt and Au for the electrocatalytic oxidation of glycerol and the pH of the electrolyte (acidic, neutral, or alkaline).^54^ Pt is known to outperform Au with respect to activity for the electrocatalytic oxidation of sugar alcohols under acidic and neutral conditions, while under alkaline conditions Au can surpass the activity of Pt.^54,67^ The latter observation has been attributed to the late (high-potential) surface oxidation of Au compared to Pt (see section 3), meaning that the surface of Au changes its features and thus its activity only at higher potentials.

Under acidic conditions (pH = 1), Pt shows four distinct regions of activity for the catalytic oxidation of glycerol based on CV^57^ and LSV combined with product analysis by online HPLC.^54,55^ In the first region, where the Pt surface is metallic (at *E* = 0.37–0.8 V), the highest current densities and product concentrations were measured, specifically at *E* = 0.8 V. In the second region, where surface PtO/PtOH_surf_ is expected to form (at *E* = 0.9–1.1 V), a substantial drop in current density accompanied by a drop in product yields was observed at *E* = 1.0 V,^54,55,57^ indicative that the oxidized Pt surface is less active for catalyzing glycerol oxidation. In the third region (at *E* = ∼1.2 V), the current density and the quantity of oxidation products increase again,^54,55,57^ indicative that this oxidized Pt surface species can show activity for glycerol oxidation at sufficiently high potential. Finally, at even higher potentials (*E* > 1.2 V), the current density increases more steeply accompanied by an increase in the formation of more oxidized products (specifically glycolic acid and FA),^54,55^ indicative that the surface under these conditions is more active for catalyzing C–C cleavage reactions.

**Scheme 8:**
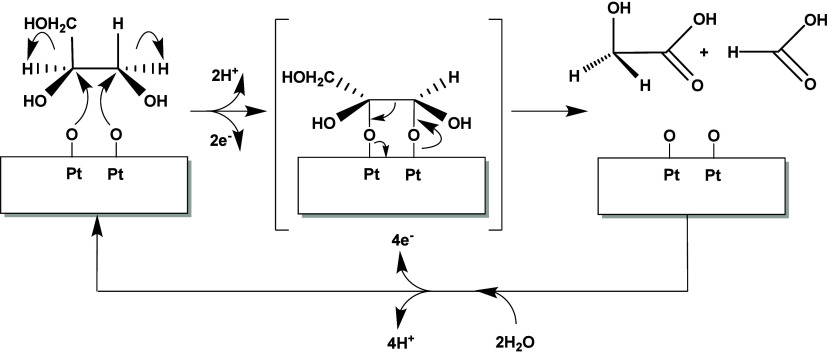
Proposed Adsorption Mechanism of Glycerol on Surface Pt Oxide (*E* > 1.1 V), Where Both the C1-OH and the C2-OH Groups Are Involved in the Adsorption, Resulting in C–C Cleavage to Glycolic Acid (Glycolate) and Formic Acid (Formate) Adapted with permission from ref (57). Copyright 1994 Elsevier.

Pt catalyzes the oxidation of glycerol under acidic conditions (pH = 1) between 0.37 and 0.6 V, mainly at the primary alcohol group, resulting in a selectivity of > 99% GALD (Scheme 6).^54,55^ With an increase in potential to 0.6–1.1 V, the main product remains GALD, but the selectivity decreases due to the parallel oxidation of the secondary alcohol group of glycerol to form DHA (∼5%) and an increase in selectivity toward GLA (i.e., the successive oxidation product of GALD) as well as the formation of CO_2_.^54,55^ In a chronoamperometric study at lower potentials and short reaction times (at *E* = 0.75 V and 20% glycerol conversion), GALD was produced with 90% selectivity,^56^ while an increase in potential (at *E* = 0.9 and 1.1 V) and longer reaction times (with a corresponding higher conversion of glycerol) resulted in a decrease in selectivity toward GALD and an increase in selectivity toward GLA (up to 87%).^56,58,118^ These studies performed with chronoamperometry also show that over Pt-based electrocatalysts at *E* ≤ 1.1 V, only minor fractions of C–C cleavage products and higher oxidation products, such as TA and HPA, are generated.^56−58^ The detection of 4% HPA after converting 69% glycerol at 0.9 V, suggests that DHA is quickly oxidized to higher oxidation products during long-term electrocatalytic experiments. LSV combined with online HPLC showed that a successive increase in potential from 1.1 to 1.5 V (at which PtO_*x*_ species are expected to be present at the electrocatalyst surface) results in a gradual increase in C–C cleavage products as was shown by an increase in selectivity toward glycolic acid, FA and CO_2_,^54,55^ which is in agreement with chronoamperometry studies where the content of C–C cleavage products increases when the potential is increased from 1.1 to 1.3 V (e.g., 20% at 97% glycerol conversion).^57,58^ Under acidic conditions, it has been suggested that the formation of the surface platinum oxide at *E* > 1.1 V changes the adsorption of glycerol and its oxidation mechanism.^57^Scheme 8 illustrates that when the O/Pt ratio increases, the interaction of two Pt–O with two C atoms from the glycerol molecule may be favored, which changes the oxidation mechanism and results in C–C bond breaking reactions.^57^ The cleavage of C–C bonds is likely catalyzed by surface Pt oxide rather than taking place via non-electrochemical reactions, since non-electrochemical reactions that induce C–C cleavage reactions, such as retro-aldol reactions only occur under alkaline conditions (see section 2.1.1). The detection of minor fractions of DHA or HPA indicate that Pt does not only catalyze the oxidation of the primary alcohol group but is also able to promote the electrocatalytic oxidation of the secondary alcohol group,^54−56,58,118^ in contrast to what has been observed for Au.^54,106,108^ It is assumed that the electrocatalytic oxidation of the secondary alcohol group is made possible by simultaneously adsorbing the glycerol molecule through both the primary carbon and secondary carbon,^22^ which is strongly dependent on the surface structure that is exposed.

**Scheme 9:**
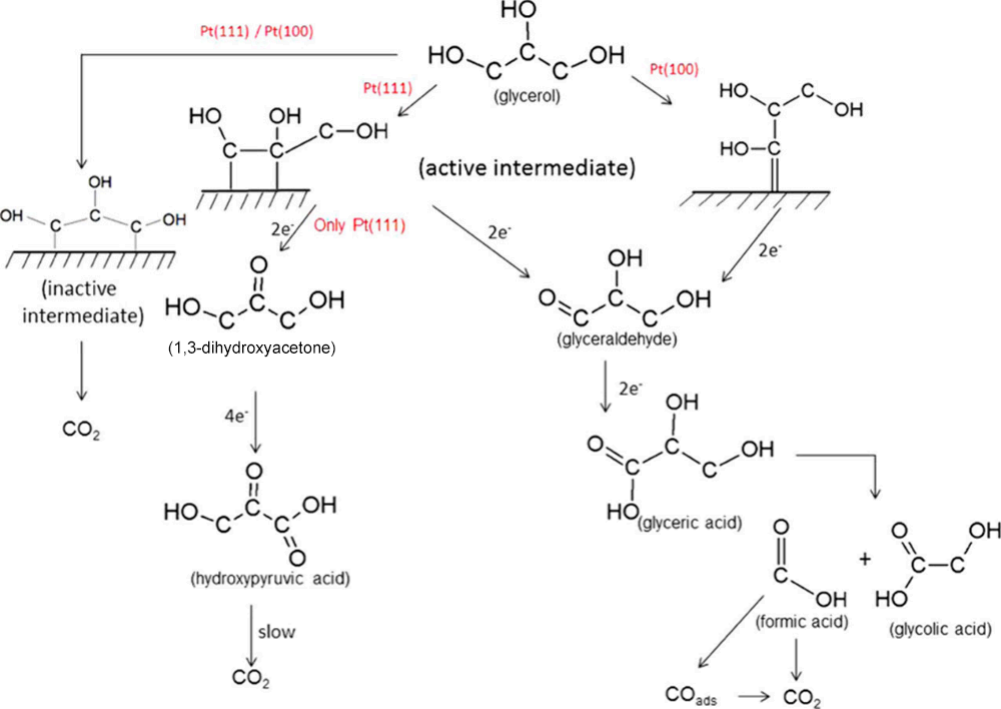
Proposed Reaction Pathways and the Corresponding Intermediates for the Electrochemical Oxidation of Glycerol on Pt (111) and Pt (100) Electrodes Reprinted from ref (22). Copyright 2016 American Chemical Society.

Metal-based electrocatalysts can be synthesized with different surface structures containing low-index facets (LIFs) or high-index facets (HIFs), which influence their performance. The HIFs have a higher density of edges, corners and kinks, which have a low coordination number and thus fewer bonds, generally making them catalytically more active.^157^ The selectivity can be tuned by modifying the electrode so that it presents a specific crystal structure, as was shown for the selective electrocatalytic oxidation of glycerol.^22,90^ The electrocatalytic oxidation of glycerol on Pt(111) and Pt(100) was investigated by combining LSV with online HPLC.^22^Scheme 9 illustrates the reaction pathways that were inferred for the electrocatalytic oxidation of glycerol on the different Pt facets and the resulting products. On Pt(111), several products were found, including GALD, GLA, and DHA, indicative of the electrocatalytic oxidation of the primary and secondary alcohol of glycerol. On the other hand, on the Pt(100) surface, only GALD was detected, thus presenting a higher selectivity toward the oxidation of the primary alcohol group. The distinct electrochemical response on the two Pt surface structures indicates that there might be different reaction intermediates on the surface, which was supported by DFT modeling:^22^ on Pt(100) the dehydrogenated glycerol intermediate binds to the surface through a Pt=C bond, while on Pt(111) the intermediate is formed through the simultaneous binding of the carbon of the primary and secondary alcohol group on the surface (Pt–C bond), therefore yielding different products on the two surfaces.^22^

Polycrystalline Pt-based electrocatalysts have also been found to be selective toward the electrocatalytic oxidation of sorbitol at the primary alcohol group in acidic medium (0.1 M HClO_4_).^158^ At *E* = 0.55 V, the (metallic) Pt-based electrocatalyst promotes the formation of glucose and, to a lesser extent, of glucuronic acid (GLU) with traces of gluconic acid (GA) and C–C cleavage products. At slightly higher potentials (0.65 V), glucose remains the major product with a slight increase in selectivity toward the successive oxidation products, GLU and GA, and traces of C–C cleavage products. This increase in potential does not induce more C–C cleavage reactions,^158^ which is in agreement with the electrocatalytic oxidation of glycerol.^54,55^ At *E* = 1.35 V, the (oxidized) Pt-based electrocatalyst leads to the formation of glucose as the major product (selectivity ∼90%), but it also promotes C–C cleavage products (8% glyoxylic acid and FA) and a very small amount of GA,^158^ confirming the results for the electrocatalytic oxidation of glycerol.^54,55,57,58^ The formation of ketoses (secondary alcohol oxidation) was not reported, in contrast to a more recent study that proved the formation of ketoses for the electrocatalytic oxidation of glycerol, erythritol, and sorbitol.^52,53^ Since both studies use similar reaction conditions, this difference is tentatively explained by the difference in analytical technique (e.g., type of HPLC column and detector), as will be discussed later (*vide infra*, section 5). A combination of LSV with online HPLC showed that the electrocatalytic oxidation of sorbitol in 0.5 M H_2_SO_4_ on Pt/C resulted in a mixture of C1-OH/C6-OH (primary) oxidation products (glucose and gulose) and C2-OH/C5-OH (secondary) oxidation products (fructose and sorbose).^53^ At *E* ≤ 0.9 V, primary alcohol oxidation products were observed (∼90% glucose and gulose) and to a lesser extent secondary alcohol oxidation products (∼5%), while at higher potentials (*E* ≥ 0.9 V, where the Pt surface is expected to be oxidized) the selectivity increases toward secondary alcohol oxidation products, resulting in ∼25% fructose and sorbose at *E* = 1.2 V.^53^ Continuous cyclic voltammetry was employed to oxidize sugar alcohols in 0.1 M HClO_4_, and the results were compared to long-term electrolysis at a fixed potential (at *E* = 0.7).^52^ In comparison to chronoamperometry (CA), the use of cyclic voltammetry between 0.02 and 1.1 V was found to improve the reaction rate by 3-fold and selectivity toward ketoses by a 3- to 5-fold, when compared to aldehydes and carboxylates, independent of the sugar alcohol (glycerol, erythritol, sorbitol) that was electrocatalytically oxidized. It was argued that the low current densities achieved during CA measurements, generally found in literature, can be related to a loss in oxidative power of Pt, as the surface reaches an oxidized (PtO_*x*_) steady state.^52^ As a result, Pt loses its oxidative activity for sorbitol. This hypothesis was supported by operando Raman spectroscopy and by injecting sorbitol at different time points during CA measurements. On the basis of Tafel slope analysis and previous research, it was postulated that the first step of sorbitol oxidation becomes rate limiting as the CA measurement proceeds. This first step involves a proton–electron transfer from an alcohol group, resulting in an oxygen-bound intermediate, which can readily be achieved on non-equilibrated Pt surfaces (e.g., surfaces that have not reached an oxidized steady state). Yet, as CA measurements proceed, the Pt surface reaches an oxidized steady state, which limits the first step of sorbitol oxidation. The increase in selectivity toward ketoses was attributed to a change in adsorption configuration of the reactant on the oxidized Pt surface. A metallic Pt surface enables the adsorption of sugar alcohols at the C2-OH/C5-OH (secondary) group, thereby promoting the oxidation of these groups and thus the formation of ketoses. These results on the electrocatalytic oxidation of sugar alcohols over Pt are consistent in the sense that both primary alcohol and secondary alcohol groups are catalytically oxidized for glycerol and erythritol, and the C1-OH/C6-OH (primary) groups and the C2-OH/C5-OH (secondary) groups are oxidized for sorbitol.^52,54−56,58^

The electrocatalytic oxidation of glycerol over Pt in neutral conditions (pH = 7) has been studied by LSV combined with online HPLC^54^ and follows nearly the same trends as under acidic conditions.^54,55^ The catalytic oxidation of glycerol starts at 0.43 V and its activity drops when PtO/PtOH_surf_ starts to form (at *E* = 0.8 V). The loss in catalytic activity continues up to *E* = ∼ 1.1 V, after which the activity increases again with an increase in potential.^54^ The current densities under neutral conditions at *E* = 0.55–1.0 V are twice as high compared to acidic conditions, while the current densities at higher potentials (*E* ≥ 1.0 V) are the same under acidic and neutral conditions.^54^ Over metallic Pt at *E* = 0.43–0.7 V, GALD is the main product, which corresponds to a 0.35 V lower onset potential compared to Au under similar conditions.^54^ However, when the potential surpasses 0.6 V, C–C cleavage reactions (leading to glycolic acid and formate/CO_2_) also start to take place, which become especially dominant at *E* ≥ 1.1 V. The formation of glycolic acid under neutral conditions starts at 0.6 V,^54^ while C–C cleavage products under acidic conditions only become substantial at 1.1 V,^54,55,57,58^ which suggests that this reaction is strongly base-catalyzed.^54^

The electrocatalytic oxidation of glycerol over Pt electrodes was also studied at pH = 13 by means of LSV combined with online HPLC^54,106^ and by means of CA combined with HPLC.^57^ The catalytic oxidation of glycerol over the metallic Pt electrode starts at 0.4 V, which is 0.4 V lower than over Au.^54,106^ The current density increases up to 0.85 V after which it quickly drops and the electrode almost completely deactivates, which coincides with PtO/PtOH_surf_ formation (*E* > 0.75 V).^54,106^ This loss in catalytic activity of Pt is at much lower potential than for Au, likely due to the earlier onset for Pt oxide/hydroxide formation compared to Au hydroxide formation (see also, section 3Figure 5).^54^ HPLC samples were neutralized as soon as they were collected, avoiding non-electrochemical reactions in the vial of the fraction collector (see section 2.1.1 on base-catalyzed non-electrochemical reactions).^54^ These studies show that over Pt at 0.4 V, GALD forms selectively (> 99%),^54^ while at higher potentials (0.7–1.4 V) the formation of GLA prevails (50–100%), although with low activity as the surface is deactivated in this potential window.^54,106^ Additionally, at > 0.6 V the formation of C–C cleavage products becomes quantitative, as was also observed under neutral conditions.^54^ Roquet et al. reported ∼90% selectivity toward GALD and minor contents of GLA, TA, and C–C cleavage products (glycolate, oxalate and formate) over Pt after 3 h of electrolysis at 0.79 V (20% conversion).^57^ CA measurements show that, as the reaction proceeds, the selectivity toward GLA and TA increases, although GALD remains the major product.^57^ Thus, it seems that at 0.79 V the sequential electrocatalytic oxidation of GALD to GLA and TA is rather slow.

Under more alkaline conditions (0.5 M NaOH), glycerol oxidation on Pt leads to 75% C3 oxidation products, where the major product was DHA (40–50%), with high contents of LA (35%) at 0.9 V (between 0.5 and 1.3 V vs RHE).^150^ This proves that at pH 13.7 a Pt catalyst can avoid the formation of high quantities of C1 and C2 oxidation products. It was also shown that no C–C cleavage reactions were observed at *E* = 1.3 V (and thus over oxidized Pt surface). These results are in contrast to earlier studies with Pt electrodes producing residual amounts of DHA and HPA.^54−56,58,118^ This discrepancy may be explained by the fact that Zhou et al. performed electrocatalytic oxidation reaction on systems containing 46 g L^–1^ of glycerol and only produced 0.08 g L^–1^ of products.^150^ At these low glycerol conversions, it is likely that only GALD was formed, as was shown in earlier research,^57^ which can easily undergo successive non-electrochemical reactions to form DHA and LA by dehydration and Cannizzaro rearrangement (see section 2.1.1) if the samples are not timely quenched.^54^ In a recent study devoted to the electrocatalytic oxidation of glycerol on a Pt electrode in 1 M NaOH, it was demonstrated that applying a pulsed potential instead of a constant potential improved the catalyst selectivity toward glyceric acid from 38% to 82%. Yet, increased selectivity toward higher oxidation products such as dicarboxylates (e.g., tartronic acid) was not achieved.^159^ Several other studies performed at 0.5–1.0 M KOH have shown that Pt is highly selective toward the formation of TA.^113,160,161^ In the case of Pt/CNT in 0.5 M KOH at 0.7 V vs RHE, up to 75–95% TA was found over a 2 h time frame,^160^ while over a P-doped Pt/MWCNT catalyst in 0.5 M KOH at 0.73 V, near equimolar amounts of TA and GLA were detected, together accounting for 90% of the final products.^161^ These results show that dicarboxylic acids (TA) are only produced under highly alkaline conditions, as was also shown for Au electrodes at pH ≥ 14.3.^105,107^ At 1.0 M KOH, Ferreira et al. showed for a Pt/C catalyst that higher current densities (90 mA cm^–2^) and hence higher overpotentials promoted C–C cleavage reactions, whereas lower current densities (60 mA cm^–2^) and overpotentials promoted the formation of TA and decreased the C–C cleavage.^113^ Doping the Pt/MCNT with P improved the stability of the catalyst, without affecting its selectivity.^161^ The increased activity was ascribed to the reduction of the accumulation of carbonaceous intermediates and an increase in the local hydroxyl concentration.^161^

In summary, the electrocatalytic oxidation of sugar alcohols under acidic and neutral conditions is substantially faster on Pt than on Au. The use of non-equilibrated Pt surfaces can improve the catalyst activity further. Under acidic and neutral conditions, Pt mainly produces GALD from glycerol through the dehydrogenative oxidation of the primary alcohol group but also catalyzes the dehydrogenative oxidation of the secondary alcohol group resulting in minor contents of DHA. The selectivity toward primary or secondary alcohol group oxidation of glycerol can be further improved with Pt electrodes that expose specific crystal structures. Moreover, surface platinum oxide has been proposed to be responsible for C–C cleavage reactions through adsorption involving the simultaneous interaction of the surface with adjacent carbon atoms. Finally, dicarboxylates (TA) can only be formed with Pt electrocatalysts under highly alkaline conditions (pH ≥ 13.7). Yet, the intrinsic electrocatalytic selectivity in alkaline media is very difficult to determine if online sample collection and immediate neutralization is not performed.

#### 4.1.3 Pd-, Ir-, and Ru-Based Electrocatalysts for the Oxidation of Sugar Alcohols

This section evaluates the performance of platinum group metals for the electrocatalytic oxidation of glycerol, as they are known to have similar physical and chemical properties. Platinum group metals that have been used for the electrocatalytic oxidation of glycerol are Pd, Ru, and Ir. The potential products formed with these catalysts were compared to Pt to seek for trends between platinum group metals.

Two studies have been devoted to the effect of the Pd surface structure on the selective electrocatalytic oxidation of glycerol.^162,163^ Both articles show a good performance of Pd nanocubes, which predominantly expose (100) planes. In both cases, only the electrocatalytic oxidation of the primary alcohol is achieved, but the selectivity differs from one system to the other. In the first case, 99% TA was obtained after 9 h electrolysis at *E* = 0.87 V and pH = 13,^163^ which is an unusual product. TA was previously only formed through electrocatalytic reactions at pH ≥ 13.7 on Au and Pt electrodes.^105,107,113,160,161^ It was argued that the high selectivity toward TA can be attributed to the high selectivity of Pd(100) toward the electrocatalytic oxidation of the primary alcohol group (section 4, Scheme 9).^163^ In the second case, at pH = 13.7 after 2 h electrolysis, GALD is the main product regardless of the potential applied (55–60% at 0.6 ≤ *E* ≤ 1.2 V), followed by 30% of oxalic acid and 5–10% of glyceric acid.^162^ Both activity and selectivity were improved when Pd(100) nanocubes were used instead of Pd nanoparticles, showing a higher oxidative current and an increased selectivity toward the oxidation of the primary alcohol.^162^ A possible explanation for the discrepancy between these two studies could be the long reaction times (9 h) applied to achieve high TA selectivity, as it requires the further electrocatalytic oxidation of GALD.^163^ The results suggest that the reaction pathway is shifted to the primary alcohol oxidation via the surface structure-induced selectivity of the Pd nanocubes exposing (100) planes.^162,163^

Pd nanocrystals on a carbon support were used to study the electrocatalytic oxidation of glycerol at pH = 13 and *E* = 0.8 V.^164^ After 4 h electrolysis, the reaction product selectivity was 45% glycolate, 40% GLA, and some minor products (TA, oxalate and FA, 5% each), evidencing substantial cleavage of C–C bonds.^164^ Under slightly more alkaline conditions (pH = 13.7), Zhou et al. showed that the electrocatalytic oxidation of glycerol at *E* = 0.8 V resulted predominantly in GALD (45%) and oxalate (45%) with minor contents of GLA.^162^ This higher selectivity toward GALD instead of GLA indicates that the conversion was lower resulting in less oxidized species, while the opposite is expected when the selectivities of oxalate and glycolate are considered. Therefore, the difference in selectivity remains to be understood. When the alkalinity of the electrolyte is increased further (pH = 14), at a potential of *E* = 0.7 V, Pd exclusively leads to the formation of GLA, while Pt leads to the formation of DHA (60%) and GLA (40%).^165^ Moreover, these studies show that over Pd the primary alcohol group is exclusively oxidized,^162,164,165^ while over Pt the secondary alcohol group can also be oxidized.^54−56,58,118^ In contradiction to these results, Ahmad et al. showed that the oxidation of glycerol over Pd/CNT at *E* = 1 V in pH = 13.7 results in a product selectivity of DHA (65%), GALD (20%), and MOA (13%), which shows that Pd might also be able to promote the oxidation of both the secondary and primary alcohol group of glycerol.^166^ Phosphorus doping of Pd/CNT further improved the catalyst selectivity by a 1.4 fold to ∼93% DHA. The studies devoted to Pd were all performed at pH ≥ 13, where it is expected that GALD isomerizes to DHA (see section 2.1.1). Yet, the formation of DHA was often not reported (with one exception^166^),^162−165^ indicative that glycerol is electrocatalytically oxidized to GALD and then to GLA without the intermediate desorption of GALD from the electrode surface, thereby preventing the isomerization of GALD to DHA.

RuO_2_/Ti and IrO_2_/Ti electrodes and a mixture of these two dimensionally stable anodes (DSA) were compared with respect to selectivity and conversion at pH = 0.3 (0.5 M H_2_SO_4_).^167^ At 1.8 V, DSA had a 30% and 60% higher conversion than RuO_2_/Ti and IrO_2_/Ti, respectively. All electrocatalysts showed similar product selectivity (∼70% of the products were quantified): 40% toward GALD and GLA, 15–18% DHA and HPA, with the remainder being C–C cleavage products. These results demonstrate that RuO_2_ and IrO_2_ can both catalyze the oxidation of the primary alcohol group as well as the secondary alcohol group of glycerol,^167^ similar to the results obtained with Pt electrocatalysts.^54−56,58,118^

In conclusion, further research needs to be conducted on these catalysts under acidic conditions and outside the OER region to evaluate better to which extent Pd-, Ru-, and Ir-based electrocatalysts can catalyze similar reactions for the electrocatalytic oxidation of glycerol as Pt.

#### 4.1.4 Ni- and Co-Based Electrocatalysts for the Oxidation of Sugar Alcohols

This section evaluates the electrocatalytic oxidation of glycerol on Ni oxide and Co oxide electrodes at different pH and potentials. A frequently studied metal oxide for the electrocatalytic oxidation of glycerol is Ni oxide,^120,168−171^ while Co oxide is less commonly studied.^111,172^ Nickel and cobalt are able to form similar metal oxyhydroxide species (i.e., NiOOH and CoOOH), which presumably catalyze similar reactions.^173^ Therefore, this section discusses the mechanism through which these metal oxides catalyze glycerol oxidation and their property-performance relations.

**Figure 7:**
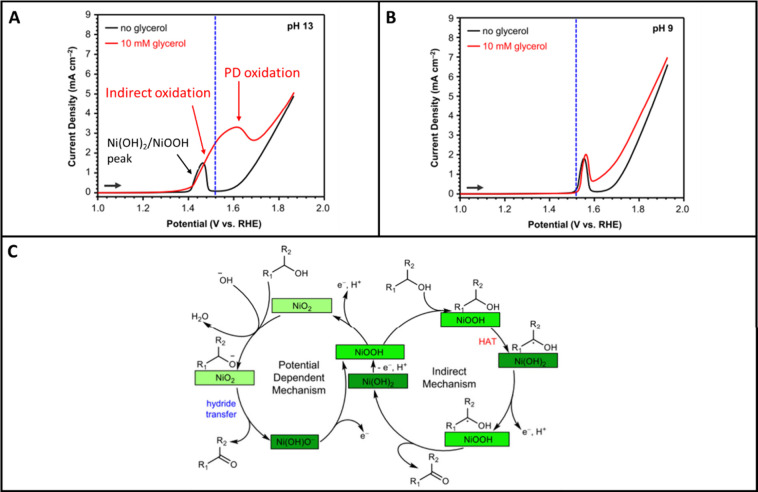
Linear sweep voltammetry of Ni in the absence (black) and presence of 10 mM glycerol (red) obtained in an electrolyte at pH = 13 (A) and 9 (B). The dashed blue lines at 1.52 V indicate the potential at which chronoamperometric experiments were performed, and the red arrows indicate the regions where NiOOH induces different reaction mechanisms. The related reaction mechanisms can be subdivided in indirect oxidation and potential-dependent oxidation (C). Adapted with permission from ref (169). Copyright 2022 Springer Nature.

Various studies have been devoted to nickel catalysts for the selective electrochemical oxidation of glycerol to value-added products.^120,168,169^ Monometallic Ni catalysts have only been found to be active for glycerol oxidation in the potential region where Ni forms β-NiOOH (*E* ≥ ∼ 1.3 V).^120,174^ More specifically, from the potential at which β-NiOOH is formed up to the potential at which OER starts, two different mechanisms were proposed through which glycerol oxidation can proceed, being either the indirect mechanism catalyzed by β-NiOOH or the potential-dependent mechanism catalyzed by NiO_2_ (Figure 7).^169,175^ For the indirect mechanism (Figure 7C), Ni(OH)_2_ is first oxidized to NiOOH and subsequently the sugar alcohol adsorbs on the surface. From this adsorbed sugar alcohol, a hydrogen atom is transferred (HAT) to NiOOH to form an α-C radical and Ni(OH)_2_. The adsorbed α-C radical successively undergoes further oxidation to form an aldehyde, GALD in the case of glycerol. The formed Ni(OH)_2_ undergoes oxidation to form NiOOH again, which can then initiate a new cycle of oxidation of glycerol.^169,175^ Thus, HAT reduces NiOOH to Ni(OH)_2_,^169,175^ which might explain the loss in the intensity of the reduction peak for NiOOH in the presence of glycerol during the negative-going scan in the cyclic voltammogram.^174^ For the potential-dependent mechanism (Figure 7C), it has been proposed that Ni(OH)_2_ is first oxidized to NiOOH and then to NiO_2_. Successively, a proton is withdrawn from the primary alcohol group of a sugar alcohol by a hydroxide ion in solution to form an alkoxide in solution. The alkoxide adsorbs on the surface of NiO_2_ and loses a hydrogen through a hydride shift, resulting in the formation of Ni(OH)O^–^ and an aldehyde.^169,175^ The Ni(OH)O^–^ is converted back to NiOOH/NiO_2_, which can catalyze the oxidation of the next sugar alcohol molecule. The indirect oxidation mechanism is pH-independent, while the potential-dependent mechanism is pH-dependent, as was shown by LSV under various pH conditions.^169,175^ As a result, the catalytic activity observed for the oxidation of glycerol through the potential-dependent mechanism decreases severely by lowering the pH (Figure 7A, B).^169^ This decrease in catalytic activity induced by lowering the pH resembles that of Au, where the rate-limiting step is base-catalyzed (section 4.1.1, Scheme 7).^79^

Goetz et al. also described how the pH influences the selectivity of the oxidation of glycerol.^169^ At 1.52 V, with an increase in pH from 9 to 13 the electrocatalyst selectivity changes from 78% DHA to 99% FA. Likewise, at pH = 11 with an increase in potential from 1.48 to 1.57 V, the electrocatalyst selectivity changes from 40% DHA and 21% FA to 26% DHA and 30% FA. The authors found a correlation between pH, direct versus indirect mechanism and DHA selectivity. On the basis of these considerations, they concluded that at lower pH, the direct, potential-dependent mechanism is largely suppressed and that the indirect oxidation promotes secondary alcohol oxidation leading to the observed higher DHA selectivity and avoiding C–C cleavage reactions.^169^ This shows that DHA is mainly formed at relatively mild alkaline conditions, meaning that NiOOH is not the best catalyst to be combined with non-electrochemical reactions to produce LA (see section 2.1.1). Therefore, the formation of LA over NiOOH has been occasionally reported, but the selectivity is often relatively low^168,170,171^ as the severe alkaline conditions in these studies promote the potential-dependent mechanism.^169^ Alternatively, at *E* = 1.5 V and pH = 14.8, NiO_*x*_ embedded in oxygen-functionalized multiwalled carbon nanotubes can catalyze the oxidation of glycerol to oxalate (∼60%) after 27 h, where the remaining 40% is CO_2_.^171^ After 27 h, the current dropped and the oxalate concentration remained unaffected, indicative that oxalate cannot be catalytically oxidized further on NiO_*x*_.

Vo et al. studied the effect of crystal facet engineering on the electrochemical glycerol oxidation reaction using octahedral and cubic Co_3_O_4_ as models.^176^ The (111)-dominated octahedral Co_3_O_4_, characterized by a higher concentration of Co^2+^ sites, showed superior electrocatalytic activity compared to the (001)-dominated cubic Co_3_O_4_, achieving ∼65% conversion of glycerol into dihydroxyacetone (DHA). The DHA production rate on octahedral Co_3_O_4_ was about 3.5 times higher than that on cubic Co_3_O_4_. These findings highlight the importance of specific reactive facets in enhancing the efficiency and selectivity of electrocatalysts. However, it should be noted that this study was carried out in 1.0 M KOH solution, and this highly basic pH is known to promote isomerization reactions that can affect the product selectivity (see section 2.1.1).

A Co-based electrocatalyst was used to study the electrocatalytic oxidation of glycerol at mild alkaline conditions (pH = 10) in a borax (Na_2_B_4_O_7_)^172^ electrolyte and at harsh alkaline reaction conditions (pH = 13.7–14).^111^ Under mild alkaline conditions, the electrocatalytic oxidation of glycerol is considered to follow two mechanisms, namely a direct and an indirect mechanism,^172^ which resembles the mechanisms described for NiOOH.^169^ In the proposed indirect mechanism, the oxidation of glycerol proceeds with the simultaneous reduction of CoOOH or CoO_2_ to Co(OH)_2_, leading to the depletion of CoOOH.^172^ In the proposed direct mechanism, glycerol is incorporated in the Co hydroxide surface and oxidized by surface adsorbed OH^–^ ions, without CoOOH consumption.^172^ The *operando* Raman spectra obtained at 1.5 V show high contents of Co(OH)_2_ species, which is a relevant species in the indirect oxidation mechanism of glycerol.^172^ By contrast, at 1.7 V higher contents of CoOOH species were observed by operando Raman spectroscopy, indicative that the reaction proceeds according to the direct mechanism. After 3 h chronoamperometry at *E* = 1.5 V, ∼0.4% glycerol electrocatalytically oxidized, with mild selectivity toward the dehydrogenative oxidation of the secondary alcohol groups of glycerol, thereby producing ∼60% DHA, ∼30% GALD, and ∼10% FA. At *E* ≥ 1.7 V, the conversion increased to ∼2.2% and the selectivity changed, decreasing the dehydrogenative oxidation of the secondary alcohol (selectivity = 40% DHA) and increasing the dehydrogenative oxidation of the primary alcohol and its successive oxidation (∼30% GALD and ∼5–7% GLA), as well as promoting C–C cleavage reactions (∼20% FA).^172^ This suggests that the indirect mechanism of CoOOH promotes the dehydrogenative oxidation of the secondary alcohol group of glycerol, leading to the formation of DHA, while the direct mechanism promotes higher oxidation products and C–C cleavage reactions. These results strongly resemble those obtained with NiOOH.^169^ However, it must be noted that this study was performed in an electrolyte with borate, which might have impacted the catalyst selectivity through the formation of borate-glycerol complexes (see section 2.1.2).

Under harsh alkaline and optimized conditions (pH = 13.7, 20 °C between 8.8 and 44.2 mA cm^–2^ and under continuous electrolyte mixing), Co-based electrocatalysts for glycerol oxidation lead to the selective production of 50–58% GLA, while the selectivity changes to 44% LA by altering the reaction conditions (pH = 14, 60 °C at 1.8 mA cm^–2^ and under continuous mixing).^111^ We argue that the higher selectivity for LA can be attributed to two effects. First, the lower current densities could potentially result in a Co species that is more active for catalyzing glycerol oxidation through the direct mechanism, thereby promoting the formation of DHA. Second, the lower current densities reduce the sequential electrocatalytic oxidation reaction of GALD to GLA, thereby promoting homogeneous reactions that convert GALD to DHA and then to LA (see section 2.1.1).

In summary, Ni- and Co-based electrocatalysts appear to promote the oxidation of glycerol through similar mechanisms, thereby affecting the selectivity in a similar manner. To prove this, it would be advised to study the selectivity of Co-based electrocatalysts at various pH and *E* in the absence of borate electrolyte.

#### 4.1.5 Cu- and Mn-Based Electrocatalysts for the Oxidation of Sugar Alcohols

This section evaluates the electrocatalytic oxidation of glycerol at different pH and potentials on Cu- and Mn-based electrocatalysts.^177,178^

Cu-based electrodes consisting of CuO species (as determined by *operando* Raman spectroscopy and XRD) have been studied for the electrocatalytic oxidation of glycerol at pH = 9–13 and *E* = 1.29–2.06 V.^177^ At pH = 13, with an increase in potential from *E* = 1.29 to *E* = 1.49–2.06 V, the catalyst selectivity changed from 70% FA, 20% GLA, and 5% DHA to 98% FA and 2% glycolate. This indicates that an increase in potential improves the selectivity toward C–C cleavage reactions for CuO. Independent of the applied potential, the formation of GALD was not detected, while GLA was detected (an oxidation product of GALD, see Scheme 6). Therefore, it was argued that DHA is isomerized to GALD, which can more easily be oxidized on CuO, as was determined by the lower onset potential measured during CV for GALD and DHA oxidation.^177^ To evaluate this hypothesis, the electrocatalytic oxidation of glycerol was studied at pH = 9 in 0.1 M borax (Na_2_B_4_O_7_) at *E* = 1.76–2.06 V. Under these conditions, the selectivity changed to 50–60% DHA and 40–50% FA.^177^ However, the use of borax has likely resulted in borate-glycerol complexes enabling the selective oxidation of the secondary alcohol of glycerol (see section 2.1.2, Figure 2). Moreover, the statement related to the isomerization of DHA to GALD under alkaline conditions is rather unlikely, since ketone structures are thermodynamically favored over saccharides (see section 2.1.1). Therefore, the reaction pathway for glycerol oxidation catalyzed by CuO remains to be fully understood.

Mn-based electrodes consisting of MnO_2_ species (as determined by *operando* Raman spectroscopy and XRD) were used to study the electrocatalytic oxidation of glycerol, GALD, and DHA at pH = 9 (0.1 M borax, Na_2_B_4_O_7_) and *E* = 1.45–1.85 V.^178^ MnO_2_ exists in the α-MnO_2_ phase at *E* = 1.45 V, while at *E* = 1.85 V the α-MnO_2_ and δ-MnO_2_ phases coexist.^178^ It was shown that α-MnO_2_ (at *E* = 1.45 V, < 0.1 mA cm^–2^) is moderately selective toward C3 oxidation products and equally selective toward the oxidation of the primary and secondary alcohol group of glycerol, resulting in the following product distribution: 38% FA, 33% DHA, and 30% GALD. By contrast, α-MnO_2_/δ-MnO_2_ (at *E* = 1.85 V, 2.5 mA cm^–2^) induces less C–C cleavage reactions, thereby increasing the selectivity of DHA and GALD to 45% and 40%. The electrocatalytic oxidation of DHA or GALD over α-MnO_2_ (at *E* = 1.45 V) resulted in low selectivities toward GLA (< 15%) where the remaining product was FA, indicative that α-MnO_2_ favors C–C cleavage reactions. By contrast, over α-MnO_2_/δ-MnO_2_ (1.85 V) GALD is oxidized to ∼75% GLA and ∼25% FA, and DHA is converted to ∼60% GLA, ∼30% GALD, and ∼10% FA. Notably, neither δ-MnO_2_ nor α-MnO_2_ catalyzes the oxidation of DHA to hydroxy pyruvic acid, instead it promotes the isomerization of DHA to GALD, which is then oxidized.^178^ The non-electrochemical isomerization of DHA at pH = 9 was excluded by performing a stability test of DHA and GALD in 0.1 M borax (pH = 9).^178^ Finally, the high selectivity toward DHA over MnO_2_ can potentially also be ascribed to the type of electrolyte used, namely containing borax (see section 2.1.2, Figure 2).

To conclude, to understand the selectivity of Cu- and Mn-based electrocatalysts toward the oxidation of glycerol, research needs to be conducted in the absence of borax electrolyte as this strongly affects the catalyst selectivity. Moreover, it is strongly recommended to evaluate the effect of pH and potential on the selectivity of Cu- and Mn-based electrocatalysts to assess the possibility of different reaction mechanisms, as was also done on Ni-based electrodes.^169,175^

### 4.2 Noble Bimetallic Electrocatalysts for the Oxidation of Sugar Alcohols

Bimetallic catalysts are generally used to improve the activity and/or selectivity of monometallic catalysts and to decrease the utilization of scarce noble metals (e.g., Au, Pt, Pd, Ir, Ru, Os, and Rh). The discussion of this class of catalysts has been subdivided in bimetallic electrocatalysts that combine two noble metals (section 4.2), a noble metal with a non-noble metal (section 4.3), and a noble metal with a post-transition metal (section 4.4). Bimetallic systems that combine two noble metals are by far most widely studied and have therefore been further subdivided in bimetallic Ag-noble metal catalysts (section 4.2.1), bimetallic Au-noble metal catalysts (section 4.2.2), and bimetallic Pt-noble metal catalysts (section 4.2.3).

#### 4.2.1 Bimetallic Ag-Noble Metal Electrocatalysts for the Oxidation of Sugar Alcohols

AgAu,^179−181^ AgPt,^182^ and AgPd^183,184^ electrocatalysts have been used for the electrochemical oxidation of glycerol^179,181−184^ and sorbitol.^180^ These studies have all been performed under alkaline conditions (pH ≥ 13),^179−184^ at which it has been shown that Ag itself has a very poor catalytic activity for the electrocatalytic oxidation of glycerol.^179,181,183^ By contrast, Ag combined with Au, Pt, or Pd is known to be active for the electrocatalytic oxidation of glycerol, and the presence of Ag was found to affect the electrocatalyst selectivity.

Alloying Ag with Au,^179^ Pt,^182^ and Pd^183,184^ (the formation of the alloys was shown by XRD^179,182−184^) was found to decrease the onset potential for the electrocatalytic oxidation of glycerol by ∼100 mV. This indicates that Ag has a similar effect on the bimetallic catalyst activity regardless of the metal with which it is alloyed. Moreover, the addition of Ag to Au promotes C–C cleavage reactions (as evidenced by FTIR^179,155^ and HPLC^181^). As a result, the selectivity toward glycolate (∼40%) and formate (∼60%) was improved for more alloyed AuAg bimetallic systems compared to non-alloyed AuAg catalysts.^181^ It was argued that this is a result of the stronger interaction of the reactants with the electrocatalyst surface due to the presence of Ag,^181,157^ though differences in surface roughness might have played a role too. In line with the results for AuAg electrocatalysts, the addition of Ag to Pt^182^ and to Pd^183,184^ was also found to improve the electrocatalyst selectivity toward C–C cleavage reactions, as was evidenced by HPLC^183^ and FTIR.^182,184^ For AgPt, the change in catalytic performance was attributed to a change in electronic properties of the Pt active sites induced by Ag.^182^ For the study on AgPd (conducted with HPLC), an increase in Ag content in the AgPd catalyst resulted in a decrease in selectivity toward C3 oxidation products (GLA, TA, MOA) and an increase in selectivity toward C2 oxidation products (oxalate and glycolate).^183^ The carbon balance was not closed, indicative that most likely also CO_2_ was formed. These results are in contrast with a more recent study, where the electrocatalytic oxidation of glycerol was studied in alkaline media using Ag modified Pt electrodes with varying Ag coverages.^148^ A higher electrocatalytic activity was obtained with AgPt than with Pt, with the activity being dependent on Ag coverage. Kinetic analysis revealed that the rate-determining step shifted from the formation of adsorbed intermediate at low overpotentials to the coupling reaction between adsorbed OH on Ag and adsorbed intermediates on Pt at higher overpotentials. It was proposed that the presence of Ag improved the kinetics through a bifunctional effect, facilitating the coupling of OH on Ag with intermediates on Pt. Quantitative product analysis indicated that Ag modification of Pt promoted the oxidation of glycerate to tartronate without promoting C–C bond cleavage.^148^

#### 4.2.2 Bimetallic Au-Noble Metal Electrocatalysts for the Oxidation of Sugar Alcohols

AuPt^81,150,185^ and AuPd^180,186,187^ electrocatalysts have been used for the electrochemical oxidation of glycerol^81,150,186^ and sorbitol.^180,185,187^ In these studies, the catalyst selectivity was almost exclusively studied under alkaline (pH ≥ 13) conditions and the products were analyzed by HPLC^81,150^ and FTIR.^186^

All studies performed on AuPt/C electrocatalysts show that both alloyed and non-alloyed bimetallic catalysts result in a similar onset potential for the electrocatalytic oxidation of sugar alcohols as on Pt, thereby being lower than that of bare Au.^81,185^ By contrast, Zhou et al. showed an ∼100 mV decrease in onset potential for their AuPt/Ag electrocatalysts for the oxidation of glycerol in comparison to Pt/C.^150^ This decrease in onset potential resembles the electrocatalysts in which Ag was alloyed with Au,^179^ Pt,^182^ and Pd.^183,184^ This suggests that the lower onset potential observed by Zhou et al. is not related to the alloyed AuPt catalyst but to an electronic effect induced by the Ag support. Under acidic conditions (pH = 1), the activity of PtAu for the electrochemical oxidation of glycerol was found to decrease with an increase in Au content.^150^ This decrease in activity can be explained by the low activity of Au in comparison to Pt for catalyzing oxidation reactions under acidic conditions (section 4.1.2).^54^ Zhou et al. showed that at *E* ≥ 0.8 V in 0.5 M KOH, core–shell PtAu/Ag NPs with intermediate Pt contents (30–40%) displayed the highest current densities.^150^ Yet, an explanation for the difference in electrocatalytic activity was not given.^150^

For well-alloyed AuPt catalysts, aimed at obtaining LA from glycerol, it was found that the production was predominantly dependent on the applied potential and alkalinity of the electrolyte, achieving the best LA selectivity (73%) at 0.45 V and 1 M KOH.^81^ These low potentials were accompanied by low conversion, while increasing the potential resulted in a higher conversion but also a decrease in the formation of LA, due to the sequential electrocatalytic oxidation of GALD into GLA and TA or the promotion of C–C cleavage reactions.^81^ Low potentials reduce the probability of sequential electrocatalytic oxidation of GALD and, therefore, enable the non-electrochemical conversion of GALD or DHA to LA (see section 2.1.1).^81^ It has also been reported that AuPt with 90% Pt on the surface results in a lower conversion and higher selectivity toward LA than AuPt with 64% Pt on the surface. The authors attributed this difference to a modification in electronic properties of Pt caused by Au, promoting the adsorption of OH, thereby promoting oxidation reactions and thus increasing the electrocatalytic activity. Consequently, the enhanced activity facilitates the successive oxidation of GALD to GLA, decreasing the non-electrochemical conversion of GALD to LA.^81^ The successive decrease in Pt surface coverage (AuPt with 15% Pt on surface) resulted in an increase in glycerol conversion and an increase in selectivity toward LA. This effect was related to insufficient Pt active sites for catalyzing the dehydrogenative oxidation of the alcohol group, thus inhibiting GLA formation.^81^ Interestingly, under relative similar reaction conditions, Zhou et al. showed that Pt_20_Au_80_/Ag NPs did not achieve a high LA selectivity (< 30%) but rather led to the production of DHA (40–80%).^150^ This high selectivity toward DHA may perhaps be related to the high content of Pt(111) facets (section 4.1.2).^22^ Nonetheless, it is expected that DHA would also get converted non-electrochemically to LA (see section 2.1.1). This discrepancy could be attributed to the low conversion over the Pt_20_Au_80_/Ag NPs reported by Zhou et al., as only ∼1% of glycerol was converted.^150^ This low conversion is linked to short experiments, meaning that the formed products (e.g., DHA) do not have sufficient time to undergo non-electrochemical reactions to form LA.^54,81^

An increase in catalytic activity for phase segregated PtAu electrocatalysts was also obtained for the oxidation of sorbitol in 0.3 M KOH.^185^ This higher activity was attributed to the sequential electrocatalytic oxidation of glucose to gluconic acid and an enhancement in C–C cleavage reactions, decreasing the electrocatalyst selectivity, as was shown by chronoamperometry at 0.9 V.^185^ The observed trend of increased C–C cleavage at *E* > 0.9 V was in line with other reports.^81,150^

#### 4.2.3 Bimetallic Pt-Noble Metal Electrocatalysts for the Oxidation of Sugar Alcohols

Studies devoted to bimetallic Pt-noble metal electrocatalysts evaluated the addition of Pd^162,188^ and Ru^112,165,189,190^ on the performance for the electrochemical oxidation of glycerol, where the selectivity was determined by HPLC.^112,165,188−190^ The influence of Pd in PtPd electrocatalysts was exclusively studied under alkaline (pH = 13.7–14) conditions,^162,188^ while the effect of Ru in PtRu electrocatalysts was studied under acidic (pH = 0.3)^190^ and highly alkaline conditions at (0.5–1 M NaOH, pH = 13.7–14^165,189^ and 4 M KOH, pH = 14.6^112^).

Hong et al. studied the electrocatalytic oxidation of glycerol on PtPd alloyed nanowires in 0.5 M KOH. The alloyed structures displayed higher current densities in the CV in comparison to their monometallic counterparts, as well as a slower current decrease during chronoamperometric experiments, indicative of a higher activity and stability.^188^

Zhou et al. synthesized Pd nanocubes (Pd NCs) and Pd nanocubes encapsulated in Pt (Pt@Pd), as was proven by high-angle annular dark-field scanning transmission electron microscopy (HAADF-STEM) and high-resolution TEM coupled to an energy dispersive X-ray spectroscopy analyzer.^162^ These electrocatalysts were compared to commercial Pt/C (10 wt %) and Pd/C (40 wt %) electrocatalysts for their activity toward the oxidation of glycerol in 1 M KOH. The Pt@Pd electrocatalyst displayed the highest current density during CV and was found to be the only electrocatalyst to produce glycolate (> 30% selectivity) at *E* = 0.65–1.25 V vs RHE during CA. Nonetheless, significant amounts of oxalate (> 10%) were produced over all these catalysts. This indicates that Pt@Pd suppresses the successive oxidation of glycolate to oxalate.^162^

At pH = 0.3, various alloyed Pt_*x*_Ru_*y*_/C electrocatalysts (the alloy phase was proven by XRD) were applied for the electrochemical oxidation of glycerol under acidic conditions (0.5 M H_2_SO_4_).^190^ Cyclic voltammetry showed that the alloyed Pt_*x*_Ru_*y*_/C electrocatalysts displayed ∼200 mV lower onset potentials than Pt/C. Moreover, Pt_5_Ru_5_/C and Pt_7_Ru_3_/C also displayed higher current densities for glycerol oxidation at *E* = ∼0.85 V, in comparison to Pt/C. Pt_5_Ru_5_/C presented a higher stability than Pt/C, although the sequential CV experiments lead to a continuous decrease of the current density. At 1.1 V, after 7 h of electrolysis, 80% and 100% of the carbon balance was closed for Pt_5_Ru_5_/C and Pt/C, respectively. This indicates that the addition of Ru to Pt/C promotes C–C cleavage reactions, resulting in the formation of CO_2_. Moreover, Pt_5_Ru_5_/C gave DHA (selectivity of 35%) as the main product, with minor fractions of GALD (17%), glycolic acid (17%), and GLA (11%). By contrast, Pt/C gave 42% GALD and 58% GLA with 1% glycolic acid. This indicates that Ru addition to Pt promotes the oxidation of the secondary alcohol group. DFT calculations show that the electropositive Ru atoms promote the interaction with the electronegative oxygen groups of glycerol (although the binding modes are the same).^190^

**Figure 8:**
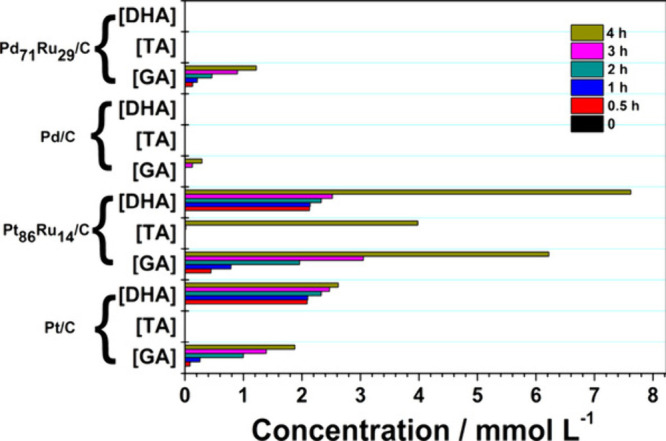
Distribution of the glycerol oxidation products as a function of time at 0.7 V vs RHE on Pt-based and Pd-based electrocatalysts in 1.0 mol L^–1^ NaOH. GA = glycerate, TA = tartronate, DHA = 1,3-dihydroxyacetone. Reprinted with permission from ref (165). Copyright 2017 Wiley-VCH.

At pH = 14, the influence of Ru in bimetallic PtRu/C and PdRu/C electrocatalysts toward the electrochemical oxidation of glycerol was studied by Palma et al.^165^ In accordance with cyclic voltammetry, Pt_86_Ru_14_/C had an onset potential of 0.5 V, ∼0.1 V lower than Pt/C, Pd/C, and Pd_71_Ru_29_/C. The lower onset potential coincides with the study conducted on PtRu/C under acidic conditions.^190^ A 60 h chronopotentiometry experiment confirmed an improved stability of the bimetallic electrocatalysts when working at low currents (3 mA cm^–2^) or low potentials (0.55–0.6 V), in comparison to the monometallic ones. FTIR and HPLC showed that at 0.7 V, Pd/C and PdRu/C promoted exclusively the oxidation of the primary alcohol group, presumably by the adsorption of this single group on the electrocatalyst surface, generating 100% GLA (Figure 8). The selectivity changed when Pt/C or PtRu/C was used, resulting in the electrocatalytic oxidation of both primary and secondary alcohol groups, resulting in a product selectivity of 60% DHA and 40% GLA for Pt/C, and 44% DHA, 33% GLA, and 22% TA for PtRu/C after a 4 h reaction time. Additionally, no carbonate was observed at the applied potential, indicative that no C–C bond breaking reactions occurred.^165^ The high selectivity toward DHA on Pt/C is somewhat surprising as most studies show that Pt is only mildly selective toward the oxidation of the secondary alcohol group.^54−56,58,118^ Therefore, we believe that DHA is likely to be a product formed through non-electrochemical processes (see section 2.1.1). The incorporation of Ru in Pt/C appeared to promote the successive oxidation of GLA to TA,^165^ which can potentially be explained by the stronger interaction of the electrocatalyst with oxygen groups of glycerol, as was suggested by DFT calculations.^190^ At pH = 14.6, the effect of different temperatures and current densities on the electrocatalytic oxidation of glycerol on a PtRu catalyst in a polybenzimidazole-based polymer electrolyte membrane reactor was investigated.^112^ At 60 °C, the main product was TA (> 60% selectivity independent of the applied current density, being 5–40 mA cm^–2^), with GLA as second product (< 30% at 20 mA cm^–2^ and decreasing at higher currents) and some glycolate. These results resemble those over Au and Pt, where similar harsh alkaline pH and temperature conditions in combination with a low applied potential resulted in an increased selectivity toward the formation of TA.^105,107,113,160,161^ By increasing the current to 80 mA cm^–2^ or the temperature to 90 °C, the oxidation of TA to oxalate and FA became more prominent.^112,92^

The observations discussed for bimetallic PtRu^165,190^ were not in line with the results with bimetallic PtRu and PtRh on graphene nanosheets obtained by Zhou et al.^189^ The degree of alloying was not discussed, yet the electrocatalysts had similar particle sizes to Pt/C. The onset potential of Pt was approximately 0.5 V, while the Pt-based electrocatalysts that contain Ru and Rh had a lower onset potential of 0.1 and 0.2 V, respectively.^189^ This lower onset potential for PtRu corresponds well with other studies.^165,190^ The peak current density of the Rh-containing electrocatalysts was 10-times higher (> 5 mA cm^–2^) than the other electrocatalysts, indicative of a higher activity. The specific reason for each catalytic activity is not clear, but the authors propose bifunctional, ligand, and strain effects as main causes. Upon an increase in potential from 0.65 to 1.25 V, the selectivity of PtRu toward glycolate increases from 20% to > 40% and the selectivity toward GALD decreases from > 30% to < 5%. In addition, the electrocatalyst showed the formation of ca. 15% oxalate and 30% GLA in the entire potential range. The formation of DHA was not reported,^189^ in contrast with other studies conducted with PtRu^165,190^ and Pt electrocatalysts.^54−56,58,118^ We argue that this can be explained by the low conversions reported in that study (∼0.1%),^189^ which would result in difficulty in the detection of DHA, which often forms a minor product.^54−56,58,118^ The low conversions reported by Zhou et al.^189^ might explain why the results in studies performed by this group often do not follow the trends observed in studies by other groups.

### 4.3 Noble-Non-Noble Bimetallic Electrocatalysts for the Oxidation of Sugar Alcohols

Research devoted to bimetallic electrocatalysts that combine noble and non-noble metals for the electrochemical oxidation of glycerol includes the investigation of the effect of Ni addition to Pt,^191^ Au,^168^ and Pd;^168^ Cu addition to Pt^192^ and Pd;^193^ and CeO_2_,^194,195^ Mn,^196^ and Fe^196^ addition to Pd.

Luo et al. studied the selectivity of Pt/C and PtNi_*y*_/C (*y* = 1, 2 or 3) for the electrocatalytic oxidation of glycerol at pH 14 and *E* = 0.9 V.^191^ The PtNi/C catalysts were characterized by HAADF-STEM-EDS, XPS, and XANES. It was shown that PtNi_2_/C has a homogeneous distribution of Pt and Ni on the surface and that the Pt in this bimetallic structure is less oxidized due to the electron-donating nature of Ni (as determined by XANES). The addition of Ni to Pt improved the overall conversion and increased the selectivity toward oxalate, glycolate, and < C2 products, indicative for the promoting effect of Ni on C–C cleavage reactions. Operando X-ray spectroscopy and UV–vis spectroscopy were used to evaluate the effect of Ni on the PtNi electrocatalyst. On the basis of these techniques, it was suggested that glycerol adsorbs strongly on the Ni(OH)_*x*_ surface, preventing the oxidation of Ni. As a result, Ni is unable to catalyze glycerol oxidation (Figure 7). Therefore, it was suggested that the addition of Ni to Pt electrocatalysts changes the electronic properties of Pt and does not result in a bifunctional mechanism.^191^ Under similar alkalinity, but higher potentials, Houache et al. studied the electrocatalytic oxidation of glycerol toward FA on Ni/C, Ni_0.9_Au_0.1_/C, and Ni_0.8_Pd_0.2_/C electrodes.^168^ Chronoamperometric measurements showed that the electrocatalytic activity is significantly enhanced due to the introduction of noble metals.^168^ The Faradaic efficiency (FE) with which FA was formed increased in the following order: Ni/C, Ni_0.9_Au_0.1_/C, and Ni_0.8_Pd_0.2_/C.^168^ Here, FE was used rather than the more commonly used product selectivity. These results indicate that under the tested reaction conditions, the presence of other metals on the Ni surface improves the catalytic activity toward C–C cleavage.^168^ Besides FA, also traces of LA were found,^168^ which can be the result of glycerol dehydrogenative oxidation by NiOOH at the secondary alcohol to DHA (section 4.1.4)^169^ and the successive non-electrochemical conversion of DHA to LA (see section 2.1.1).

Mürtz et al. studied glycerol electrocatalytic oxidation at pH = 0.3 and *E* = 1.1 V on Pt, Pt_8_Cu_2_, Pt_7_Cu_3_, and Pt_5_Cu_5_. The metal particles had similar size (∼2 nm, as determined by STEM), were found to be alloyed (shown by XRD), and similar metal loadings were used to study their electrocatalytic performance.^192^ The current density for the electrocatalytic oxidation of glycerol was higher for PtCu electrocatalysts than for bare Pt. Moreover, the addition of Cu to Pt decreased the yield toward C–C cleavage reactions by ∼10% for Pt_8_Cu_2_, reaching 90% C3 products, while higher Cu contents resulted in similar amounts of C–C cleavage reactions with respect to bare Pt. In comparison to bare Pt, the PtCu electrocatalysts were also not able to promote the successive oxidation of GLA to TA, resulting in high selectivity toward GALD (60%) and GLA (30%).^192^ The lower selectivity of PtCu toward C–C cleavage products and TA was attributed to a weaker adsorption strength of the intermediate products on the electrocatalyst surface, thereby decreasing successive oxidation of GALD and GLA.^192^ The lower adsorption strength of intermediate products was inferred from the higher ratio between peak current density in the forward and backward scan measured during cyclic voltammetry in the presence of glycerol.

Mo et al. studied the electrocatalytic oxidation of glycerol at pH = 14 and *E* = 0.8 V on Pd_75_Cu_25_, Pd_50_Cu_50_, and Pd_25_Cu_75_ electrocatalysts.^193^ These catalysts were synthesized by a laser-assisted nanomaterial preparation method that does not require solvents and can be performed under atmospheric pressure and room temperature. Each electrocatalyst was prepared by coating a 10 nm layer of Cu or 10 nm Pd or a mixture of the two on a polyimide film. The resulting electrocatalysts consisted of alloyed structures as proven by XPS and HAADF-STEM-EDS. Under these reaction conditions, the current density was the highest for Pd and decreased with increasing Cu content and was zero for Cu,^193^ which contrasts the results obtained with PtCu under acidic reaction conditions.^192^ This indicates that the Cu oxides/hydroxides at the surface of the Cu electrode are not active for glycerol oxidation. The selectivity of the Pd electrocatalyst was ∼60% GLA, ∼8% GALD, and 9% TA, with the remaining products being C1 and C2 compounds, while an increase in Cu content up to Pd_50_Cu_50_ resulted in a decrease in selectivity toward C–C cleavage products down to 1% and an increase in selectivity to GALD (∼83%), thereby reducing the rate of C–C bond breaking.^193^ These results strongly resemble those of PtCu electrodes.^192^ For Pd_25_Cu_75_, the only products were C1 and C2 compounds, indicative of an increase in C–C cleavage reactions. This shows that there is an optimum in Cu content in PdCu to steer the electrocatalyst selectivity toward C3 products, thereby reducing C–C cleavage reactions. The preference of PdCu for C3 products was attributed to a synergetic effect between the two metals, as supported by DFT calculations and in situ XAS experiments.^193^

Instead of alloying Pt with another metal, the effect of successive functionalization of carbon nanotubes (CNTs) with cerium oxide (CeO_2_) as support for Pt nanoparticles (Pt-CeO_2_/CNT) was studied.^194,195^ Li et al. showed a uniform distribution of Pt and CeO_2_ over the CNTs and claimed a ternary interaction, where the Pt-CNT interaction assures electrical conductivity and the Pt-CeO_2_ interaction leads to a modification of electronic properties (as shown by an upshift in binding energy in XPS).^194^ The CeO_2_ also improves the dispersion of Pt particles on the CNTs, leading to an increased number of active sites.^194^ Moreover, the downshift in the *d*-band center of Pt weakens the interaction with GLA during the electrocatalytic oxidation of glycerol,^197^ which was claimed to lead to the observed improved recycling stability by 2-fold^194^ and catalyst activity by 5-fold.^195^ Under relatively similar reactions conditions, Liu et al. showed that the selectivity of Pt is not significantly affected by CeO_2_,^195^ while Li et al. showed a higher selectivity toward TA and C–C cleavage and lower selectivity toward LA.^194^ The increased selectivity toward TA suggests that CeO_2_ promotes oxidative reactions but also causes successive C–C cleavage reactions.^194^ Moreover, we argue that the lower selectivity toward LA for Pt-CeO_2_/CNT can be related to a faster oxidation of GALD preventing its desorption and the successive non-electrochemical reactions to form LA (see section 2.1.1). A further optimization between the CeO_2_-to-Pt ratio on the CNT support decreased the selectivity toward the C–C cleavage reaction products to 4% and resulted in 64% GLA, 6% TA, and 26% LA.^194^

Naik et al. studied the effect of the support on a Pd electrocatalyst.^198^ In this case, C and TiO_2–*x*_ nanosheet (NS) supports were decorated with PdZn for the electrocatalytic oxidation of glycerol in 0.5 M NaOH (pH = 13.7). Here, it was shown that the TiO_2–*x*_ NS caused a downshift in the *d*-band center. Cyclic voltammetry showed that the TiO_2–*x*_ NS support causes a positive shift in the PdO_*x*_ reduction peak when compared to a carbon support, suggesting that Pd binds oxygenates species less strongly on the TiO_2–*x*_ NS support. It was claimed that this might decrease the poisoning effect of adsorbed oxygenated species formed during the oxidation of glycerol, thereby improving the electrocatalyst stability and activity, similarly to what was shown for Pt/CNT functionalized with CeO_2_. .^194,195^ The support did not show a significant effect on the electrocatalyst selectivity, resulting in near ∼45% GLA and ∼45% LA. In this, case glycerol is electrochemically oxidized to GALD and successively electrochemically oxidized to GLA or alternatively non-electrochemically converted to LA.

Finally, the addition of Mn and Fe to Pd electrocatalysts was studied for glycerol oxidation at pH = 13. MnPd/C and FePd/C had a 2- and 1.5-fold higher current density than Pd/C.^196^ Moreover, the addition of Mn or Fe to Pd changed the electrocatalyst selectivity. After CA at 0.8 V, Fe promoted the selectivity toward FA by 2-fold, inducing C–C cleavage reactions, while Mn promoted the selectivity toward GLA and TA by 2-fold, reducing C–C cleavage reactions.^196^

### 4.4 Noble-Post-Transition Bimetallic Electrocatalysts for the Oxidation of Sugar Alcohols

The modification of noble metal electrocatalysts by the addition of post-transition metals and their effect on the electrochemical oxidation of various sugar alcohols has focused nearly exclusively on Pt electrodes,^53,55,90,118,167,199−203^ with a few examples of Pd electrodes.^153,203^ Moreover, only the studies devoted to Pt electrodes have reported the product concentrations analyzed by HPLC. Most of these studies were conducted with adatom-modified electrocatalysts,^53,55,90,153,199−202^ while a few have also alloyed the noble and post-transition metals.^118,167,203^ These studies were frequently performed under acidic conditions^53,55,90,118,167,199,200^ and less often under alkaline conditions.^153,201−203^ The most commonly studied post-transition metal is Bi,^53,55,90,118,167,200,201,203^ but other post-transition metals have been also investigated, such as Sb,^53,118,200^ Sn,^53,199,200^ Pb,^53,200,202^ and In.^53,200^

**Figure 9:**
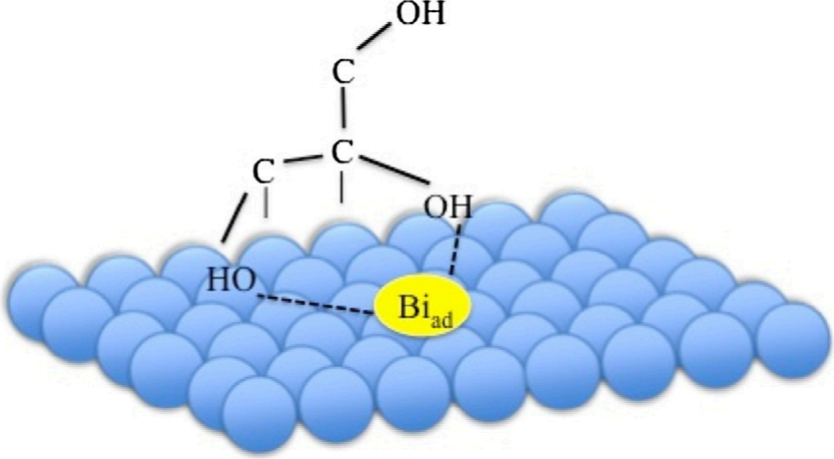
Proposed interaction between the enediol intermediate and Bi on Pt (111) surface. Reprinted with permission from ref (90). Copyright 2017 Elsevier.

The addition of Bi adatoms on Pt(111) and Pt(100) facets was studied under acidic conditions (0.5 M HClO_4_) by LSV combined with online HPLC and *in situ* FTIR.^90^ As illustrated in Figure 9, it was suggested that Bi on Pt(111) interacts specifically with the enediol intermediate, thereby promoting the isomerization toward DHA, and consequently steering the electrocatalyst selectivity toward the oxidation of the secondary alcohol.^90,204^ On the other hand, on Pt(100) Bi decreases the activity and does not impact the formation of GALD and GLA. In the case of Pt(100), the surface-adsorbed enediol intermediate does not exist, as glycerol only binds through a single primary carbon (see Scheme 9), thus leading exclusively to the electrocatalytic oxidation of the primary alcohol.^22^ For Pt(111)-Bi, Bi adatoms suppress the formation of adsorbed CO and thus the catalyst poisoning.^90^ For Pt(100)-Bi, Bi adatoms only partially prevent the formation of adsorbed CO species.^90^ The decrease of CO poisoning induced by Bi adatoms show a resemblance to PtBi bimetallic catalysts.^203^ These results show that the electrocatalyst structure has a strong influence on how the adatoms modify the catalyst performance.^90^ A similar effect was observed in a study on the effect of Sn-adatom-modification of Pt(100) preferentially-oriented nanoparticles in acidic media. The presence of Sn adatoms on Pt surfaces alters the adsorption of glycerol on Pt, consequently hindering the breaking of C–C bonds. As a result, the selectivity of the Sn-modified Pt(100) preferentially-oriented nanoparticles is diverted to C3 oxidation products, while the preference toward the electrocatalytic oxidation of the primary alcohol remains unaltered.^199^

**Figure 10:**
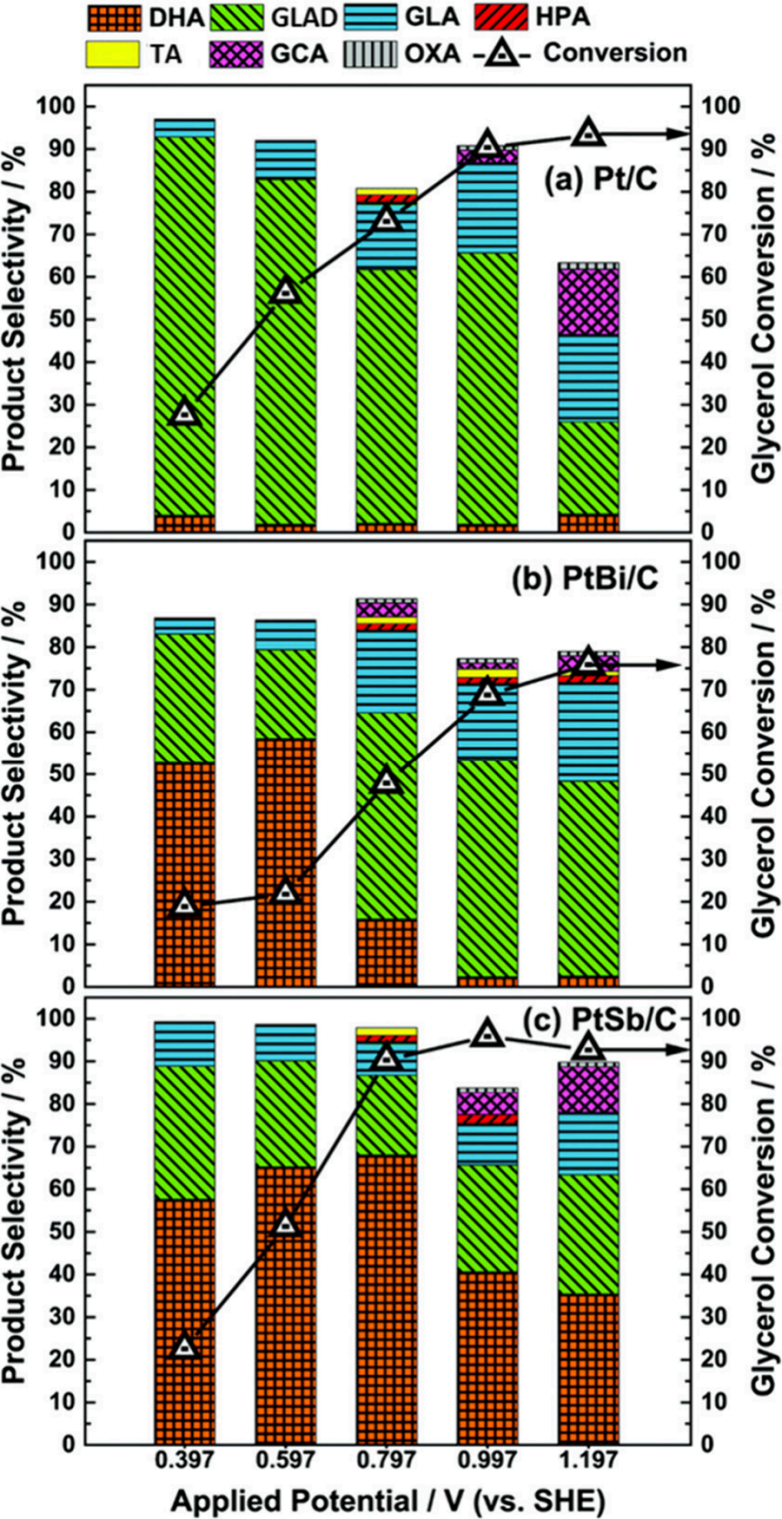
Effect of alloying Sb or Bi with Pt/C in the electrocatalytic oxidation of glycerol in 0.5 M H_2_SO_4_ as a function of applied potential (GCA = glycolic acid and OA = oxalic acid). Reprinted with permission from from ref (118). Copyright 2016 Royal Society of Chemistry.

Most literature studies have been devoted to the effect of adatoms on polycrystalline Pt under acidic conditions.^53,55,200^ Bi and Sb are adsorbed on Pt surfaces under acidic conditions (0.5 M H_2_SO_4_) at low potentials, while at *E* = 0.61–0.66 V both adatoms start to oxidize.^205,206^ At *E* > 0.85 V, Bi and Sb progressively desorb.^205,206^ Bearing this in mind, the highest impact on the Pt electrocatalyst selectivity is obtained when Bi or Sb adatoms are not oxidized (*E* ≤ 0.6 V).^53,55,200^ In this region, Bi alters the reaction pathway: (1) by blocking the active Pt sites, which induce the electrocatalytic oxidation of the primary alcohol; (2) by changing the coordination of the adsorbed sugar alcohol on the Pt surface, which redirects the electrocatalyst selectivity toward secondary alcohol oxidation reactions; and (3) by preventing C–C cleavage reactions, thereby reducing CO formation.^53,55^ As a result, Bi- or Sb-adatom-modified Pt/C electrocatalysts have been found to be highly selective toward the oxidation of the secondary alcohol groups of various sugar alcohols at *E* < 0.6 V,^53,55,200^ achieving a selectivity of nearly 100% DHA.^55,200^ At *E* > 0.6 V, the adatoms progressively lose their effect on the selectivity due to their oxidation and desorption from the surface of Pt.^53,55,200^ Similar results were obtained with alloyed PtBi and PtSb electrocatalysts under acidic conditions (0.5 M H_2_SO_4_) for the oxidation of glycerol.^118,167^ Briefly, at *E* = 0.4 and 0.6 V (Figure 10), PtBi/C and PtSb/C showed similar selectivity toward the oxidation of the secondary alcohol group, producing DHA with 50–65% selectivity.^118^ The selectivity toward DHA for PtBi/C changed at *E* = 0.8 V and *E* ≥ 1.0 V to 15% and 2%, respectively, while the selectivity toward DHA for PtSb/C increased at *E* = 0.8 V first to 68% and then decreased at higher potentials to ∼42%.^118^ We argue that the discrepancy between research on Sb in PtSb alloyed electrodes and Sb-adatom-modified Pt electrodes might be related to the structural properties of the electrocatalyst, where the incorporation of Sb adatoms in the fcc Pt structure limits its desorption at *E* > 0.85 V (typically observed for Sb adatoms on Pt electrodes^205^). Moreover, the use of Pt_*x*_Bi_10–*x*_/C (*x* = 1, 5, or 9) electrocatalysts for the oxidation of glycerol in 0.5 M H_2_SO_4_ at 1.2, 1.6, and 2.0 V vs RHE only resulted in minor contents of products obtained through the oxidation of secondary alcohol groups (∼15% DHA and HPA).^167^ For Bi-adatom-modified Pt/C electrocatalysts, a saturation of the electrolyte with Bi further improved the selectivity toward DHA.^55^ For this system, it is worthwhile to study whether Bi can form a complex in solution with glycerol, as was observed for borate,^102^ which can interact with the primary alcohol groups and let the secondary alcohol group coordinate toward the surface of the electrode. For Pb-adatom-modified Pt electrocatalysts, a slight change in selectivity toward DHA was also induced, although the main reaction remained the oxidation of the primary alcohol group, yielding GALD and GLA.^200^ Sn and In did not appear to change the electrocatalyst selectivity significantly.^200^

**Figure 11:**
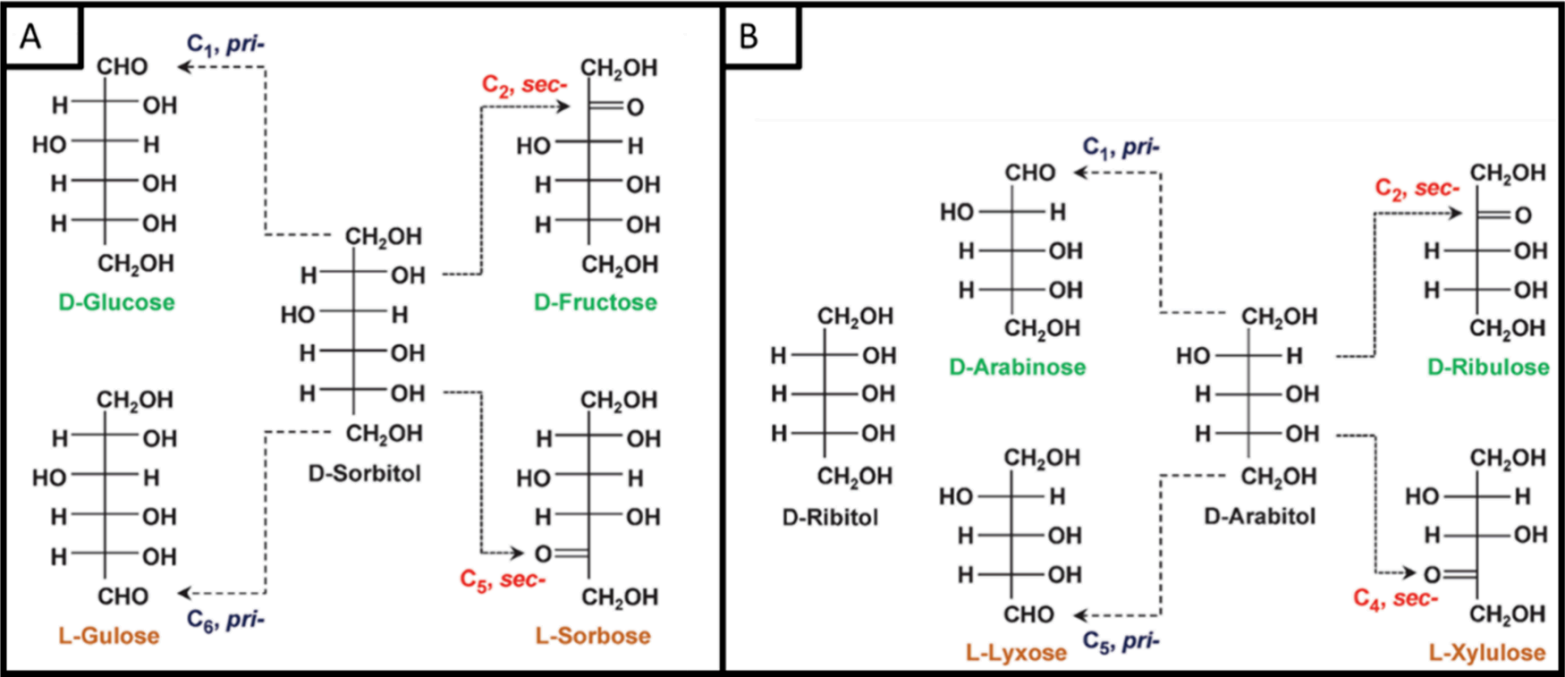
Pathways for the electrocatalytic oxidation of sorbitol and arabitol, which can be achieved through adatom modifications of Pt electrodes. Reprinted with permission from ref (53). Copyright 2015 Wiley-VCH.

The effect of Bi and Sb adatoms on Pt electrodes was also studied for the electrocatalytic oxidation of C6 (sorbitol), C5 (arabitol and ribitol), and C4 (erythritol and threitol) sugar alcohols.^53^ This study was devoted to the stereochemistry of C4, C5, and C6 sugar alcohols at the secondary alcohol position and how this affects the activity and selectivity of Bi- and Sb-adatom-modified Pt/C electrodes.^53^ Bi and Sb adatoms on Pt/C electrodes can direct the electrocatalytic oxidation of sugar alcohols (sorbitol and arabitol) toward the secondary alcohol at *E* < 0.6 V vs RHE under acidic conditions (0.5 M H_2_SO_4_), while monometallic Pt/C would mainly promote the oxidation of the primary alcohol. A general route for the electrocatalytic oxidation of sorbitol and arabitol is given in Figure 11. In the presence of sorbitol (C6), following Figure 11, Pt/C preferably leads to oxidation of the primary alcohol groups, resulting in a mixture of glucose and gulose, while Bi- or Sb-modified Pt/C diverts the selectivity to the C2-OH and C5-OH groups, yielding a mixture of fructose and sorbose. In the case of arabitol (C5), which has a similar stereochemistry to sorbitol but with the C4-OH group (equivalent to the C5-OH group of sorbitol) switched, the selectivity of the electrocatalyst also changes.^53^ For Pt/C, the selectivity was directed to the oxidation of the primary alcohol groups, with a higher preference for the C1-OH group than for the C5-OH group. Bi-Pt/C on the other hand shows an enhanced selectivity toward both the electrocatalytic oxidation of the C2-OH and C4-OH groups, thus diverting the selectivity of Pt/C again away from the oxidation of the primary alcohol groups (C5-OH and C1-OH groups). These results highlight that the selectivity of the electrocatalyst with and without adatom modification is greatly influenced by the stereochemistry of the reactant. Besides a change in selectivity, adatoms also decrease the onset potential of Pt/C. The decrease in onset potential is adatom-dependent and reactant-dependent. The decrease in onset potential for glycerol was 150 mV for Sb and Sn and 50 mV for Bi, In, and Pb,^55,200^ and the onset potential for sorbitol decreased by 50 mV for Sn, remained unaltered for Bi, Sb, and In, and increased by 50 mV for Pb.^53^

Under alkaline conditions (pH = 13), only Bi- and Pb-adatom-modified Pt/C have been reported.^201,202^ Bi has been shown to limit the C–C cleavage reactions at *E* < 0.8 V vs RHE (based on HPLC), while at higher potentials this effect is lost,^201^ possibly due to the desorption of Bi adatoms.^205,206^ Unlike the studies performed with adatom-modified Pt/C electrodes under acidic conditions,^53,55^ the formation of DHA was not clearly detected under alkaline conditions.^201,202^ The main product formed in this case was either GLA or glycolic acid.^201,202^ It was argued that the hydroxide ions in the electrolyte can catalyze the conversion of the formed DHA into GLA.^201,202^ However, this reaction is unlikely to proceed when reaction Scheme 6 is followed.^54^ We propose that GLA and glycolic acid might have formed due to the presence of oxygen in the alkaline electrolyte (see section 2.1.1).

Bi adatom modification of Pd did not affect the electrocatalytic activity, but it did decrease the onset potential by ∼150 mV and change the reaction pathway.^153^ The reaction products were only determined on the surface of the catalyst by FTIR. At pH = 13 and *E* ≥ 0.65 in the presence of Bi, the electrocatalytic oxidation of the secondary alcohol group of glycerol takes place, resulting in the formation of DHA. Bi adsorbs on the Pd surface decreasing the number of Pd-sites surrounded by adjacent Pd atoms, resulting in two distinct effects on the reaction pathway. First, it diminishes the dissociative adsorption of glycerol (C–C cleavage) on Pd.^153^ Second, the adsorption of the primary alcohol is limited as it requires three adjacent Pd adatoms, while the secondary alcohol requires only one or two adjacent Pd atoms. Another explanation for the enhanced formation of DHA is given by the basicity that Bi offers. In the presence of Bi, the local pH increases through the adsorption of hydroxide ions, leading to the formation of a very reactive CH_2_OH-CHO^–^-CH_2_OH alcoholate that is converted into DHA.^153^ This can promote the reaction pathway toward the formation of DHA, hydroxypyruvate, and MOA.^153^ The redirection of the selectivity toward the electrocatalytic oxidation of the secondary alcohol group of glycerol was also found for the addition of Bi or Sb adatoms on Pt electrodes under acidic conditions for the electrocatalytic oxidation of various sugar alcohols.^53,55,200^ However, these studies mainly show that the selectivity is improved toward DHA when Bi is not oxidized (*E* ≤ 0.6). This discrepancy could potentially lie in the analytical technique that has been applied to identify the products or the use of alkaline rather than acidic reaction conditions.

### 4.5 Other Electrocatalysts for the Oxidation of Sugar Alcohols

Other electrocatalysts that have been used to study the oxidation of glycerol are boron-doped Co (CoB)^102^ and BiNi/C,^120,207^ where the carbon support of BiNi/C was also modified with cerium oxide and antimony tin oxide.^120^

It has been shown that the addition of Bi to Ni can improve the electrocatalyst activity as well as selectivity toward C3 oxidation products.^120,207^ At 1.35 V and pH = 14, glycerol is oxidized over Ni/C with a selectivity of ∼35% toward C3 oxidation products, whereas NiBi/C gave a selectivity of ∼50% toward C3 oxidation products.^120^ The effect of storing (e.g., aging) the catalyst ink used for drop casting NiBi/C on a glassy carbon electrode to perform electrochemical experiments was evaluated. Aging the NiBi/C catalyst caused structural deformations and morphological changes (as determined by HAADF-STEM, STEM-EDS, and STEM-EELS), exposing more Bi-based particles on the surface. The higher percentage of exposed Bi and spatial distribution of Ni(OH)_2_ and Bi(OH)_3_ improved the electrocatalyst selectivity to C3 products by reducing C–C cleavage reactions.^207^ Under optimized reaction conditions (pH = 14, *E* = 1.3 V, and *T* = 50 °C), the selectivity of the NiBi/C catalyst could be steered to 60% GLA, 10% TA, 5% LA and 25% C–C cleavage products, indicative of a moderate selectivity for the electrocatalytic oxidation of the primary alcohol group.^207^ The effect of modifying NiBi/C and Ni/C electrocatalysts with CeO_2_ and antimony tin oxide (ATO) on the selectivity was also studied at pH = 14.^120^ At 1.35 V, ATO-modified Ni/C electrocatalysts displayed > 99% selectivity toward LA. On the other hand, ATO-modified NiBi/C electrocatalysts were more selective toward GLA (70%), with minor contents of TA (10%) and LA (14%). The addition of CeO_2_ to Ni/C and NiBi/C electrocatalysts gave similar results with respect to selectivity, but the addition of CeO_2_ to Ni/C only gave 60% LA with 40% GLA selectivity.^120^ Moreover, the electrocatalytic activity of Ni/C and NiBi/C appears to be lower when modified with metal oxides, as was shown after 1 h of CA.^120^ These results potentially indicate that the electrocatalyst primarily produces GALD, which successively undergoes a series of non-electrochemical reactions to form LA (see section 2.1.1).^120^ Nickel boride catalysts have also been synthesized and their composition and the reaction conditions (pH = 14.3 and *E* = 0.5 V) have been optimized for the electrocatalytic oxidation of glycerol to lactic acid, although only 9% lactic acid was achieved where the remainder were C–C cleavage products, being mainly FA.^170^

In another study, Co was combined with borate to yield cobalt borate and used for the electrocatalytic oxidation of glycerol at *E* = 1.56–1.86 V between pH = 7.6–9.6.^102^ The effect of pH has been explained in section 2.1.1, while the effect of potential will be addressed here. With an increase in potential the selectivity toward DHA decreases, while that toward HPA increases. This change in selectivity was attributed to the higher potential applied, promoting successive oxidation reactions.^102^ We argue that higher potentials could drive the formation of more high-valent redox mediator Co^3+^/Co^4+^ species that can promote the successive oxidation of DHA to HPA.^208^ Under harsh alkaline and optimized conditions (pH = 13.7, 20 °C, between 8.8 and 44.2 mA cm^–2^ and under continuous mixing), Co could selectively produce GLA with 50–58% selectivity, while the selectivity could be changed toward 44% LA by altering the reaction conditions (pH = 14, 60 °C, at 1.8 mA cm^–2^ and under continuous mixing). The latter set of reaction conditions could reduce the sequential electrocatalytic oxidation reaction on Co of GALD to GLA, while it promoting non-electrochemical reactions that convert GALD to LA (see section 2.1.1).

## 5 Effect of Electrocatalyst Properties under Various Reaction Conditions on the Oxidation of Saccharides

**Scheme 10:**
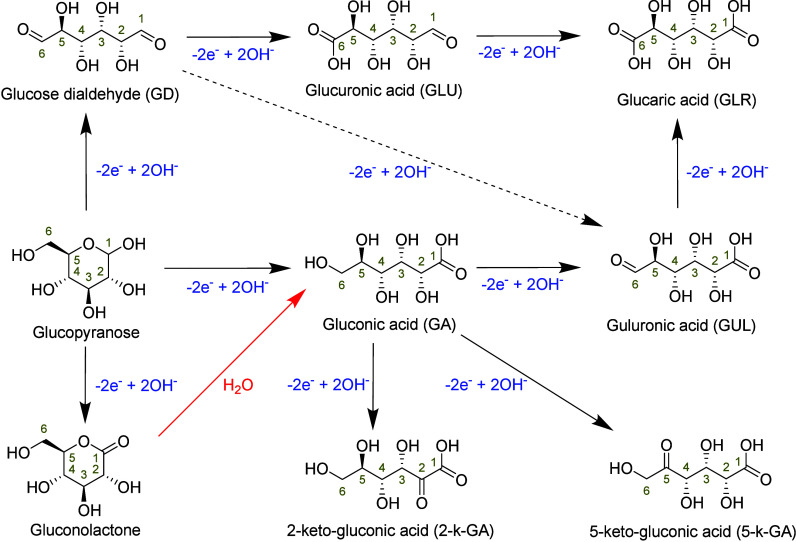
Main Reaction Pathways for the Electrocatalytic Oxidation (Black Arrows with Number of Electrons/Hydroxides in Blue) and Non-Electrochemical Conversion (Red Arrows) of Glucose and Derivatives Observed in the Literature^209^

This section presents the literature and discusses trends of the electrocatalyst activity and selectivity for the electrochemical oxidation of saccharides. To define trends for the selective electrocatalytic oxidation of the anomeric carbon in saccharide molecules, glucose is chosen as a reference compound, considering its abundance in nature and the higher number of publications about its electrochemical oxidation in comparison to other monosaccharides (e.g., fructose, xylose or mannose). Most of the studies dealing with this topic used Au and Pt (sections 5.1.1 and 5.1.2, respectively) as electrocatalysts, for which it was possible to define trends with respect to the electrocatalyst properties, i.e., the type of metal used, the oxidation state of the metal and the type of bimetallic catalyst. Before going into detail in the trends observed in the literature, the main mechanistic pathway for the electrocatalytic oxidation of glucose is summarized. A general scheme presenting the routes through which glucose can be electrocatalytically oxidized is given in Scheme 10.^209^ Under a broad range of pH conditions (pH = 1–13), glucose is predominantly present in its pyranose form (>99.9%).^70^ On the basis of FTIR, the electrocatalytic oxidation of glucose proceeds first at the anomeric carbon by dissociative adsorption resulting in an adsorbed dehydrated intermediate.^60,72^ This adsorbed intermediate can either be oxidized to gluconic acid (GA) or it can be oxidized to gluconolactone, which successively desorbs from the catalyst surface and hydrolyzes non-electrochemically to form GA, as was shown by cyclic voltammetry and FTIR.^72,210^ The hydrolysis of gluconolactone is dependent on the pH of the electrolyte: under acidic conditions, gluconolactone and GA are in equilibrium in the electrolyte, while in alkaline media the lactone ring of gluconolactone hydrolyzes non-electrochemically to yield GA.^211^ To our knowledge, gluconolactone has never been quantitatively analyzed after electro-oxidizing glucose, independently of the pH used. This is believed to be a result of the fast hydrolysis of gluconolactone to gluconic acid in alkaline electrolytes or be due to the incomplete separation of analytes by chromatographic techniques. The simultaneous quantification of gluconic acid and gluconolactone can potentially be achieved by 2D-NMR.^41^

The subsequent electrocatalytic oxidation of GA can either take place through a dehydrogenation reaction at the C2-OH group or the C5-OH group, yielding 2-keto gluconic acid (2-k-GA) and 5-keto gluconic acid (5-k-GA), respectively.^71,209,212^ These products have only been reported in three publications where a Pt electrode was used.^71,209,212^ However, some analytical techniques do not allow discerning the presence of these products due to the strong resemblance of their chemical structure with other glucose oxidation products. Therefore, the use of ternary amine columns in HPLC or a trimethylsilylation treatment followed by gas chromatography or high-pressure anion-exchange chromatography have been suggested for their separation and quantification.^71,209,212^ Alternatively, the C6-OH group of GA can be oxidized to an aldehyde, forming guluronic acid (GUL), which has rarely been quantified.^119,127,209^ In contrast to GUL, glucuronic acid (GLU) has been quantified more frequently,^28,91,209,213^ tentatively explained by the strong resemblance in the structure of GUL and GLU (e.g., being stereoisomers),^209^ hampering their separation by chromatographic techniques. Only one study enabled the discrimination of these species through the use of high-pressure anion-exchange chromatography.^209^ Alternatively, glucose can be dehydrogenated at the C6-OH group to form glucose dialdehyde, which has only been quantified once in literature by using high-pressure anion-exchange chromatography.^209^ Successively, GD can either be oxidized at C1=O or C6=O to a carboxylate yielding GUL and GLU, respectively. Finally, GUL and GLU can be oxidized at the aldehyde group to form a carboxylate, resulting in glucaric acid (GLR).

In general, several glucose oxidation products have been quantified only rarely, such as glucose dialdehyde, 2-keto gluconic acid, 5-keto gluconic acid, and either glucuronic acid or guluronic acid. This indicates that several products are likely to be overlooked. Therefore, caution should be taken in drawing conclusions from studies on the electrocatalytic oxidation of glucose and potentially also of other saccharides, such as xylose, galactose, and mannose.

### 5.1 Monometallic Electrocatalysts for the Oxidation of Saccharides

In this section, the trends for the electrocatalytic oxidation of saccharides are discussed and compared with the trends for the electrocatalytic oxidation of sugar alcohols. The structure of this section follows the same order as in the sugar alcohol section (section 4), where Au (section 5.1.1) and Pt (section 5.1.2) were studied most frequently and therefore discussed first, followed by a comparison between Pd-, Ir-, and Ru-based electrocatalysts (section 5.1.3), Ni- and Co-based electrocatalysts (section 5.1.4), and Cu- and Mn-based electrocatalysts (section 5.1.5).

#### 5.1.1 Au-Based Electrocatalysts for the Oxidation of Saccharides

This section evaluates the oxidation of saccharides at different pH and potentials on Au electrocatalysts. The electrocatalytic oxidation of saccharides, such as glucose, mannose, and galactose over Au electrodes, has mainly been studied under alkaline conditions (pH = 10–13)^28,91,110,214−218^ and less frequently neutral (pH = 7) conditions.^69,76^ To our knowledge, only Kokoh et al. studied the oxidation of glucose on Au electrodes under acidic (pH = 1) conditions.^69^ This is related to both a higher reactivity of saccharide-like compounds under alkaline conditions (see section 2.1.1) and low activity of Au under acidic and neutral media, as it will be further detailed.

The effect of Au (nano)particle surface shapes and sizes (rod, spherical, cuboid, and polyhedral shapes) and facets on the electrocatalyst activity has been studied for the electrochemical oxidation of glucose in 0.1 M NaOH.^117,219−221^ Although these studies did not evaluate the effect of the different parameters on the reaction selectivity (i.e., revealing a gap in fundamental research that needs to be addressed to design improved electrocatalysts), they all converge on the higher activity (current density) of the electrocatalysts containing Au(100) facets, followed by the Au(110) facets and Au(111) being the least active. This is in line with previous research performed on single crystals studied under acidic^103^ and neutral reaction conditions.^222,223^

Under acidic conditions in 0.1 M HClO_4_ (pH = 1), polycrystalline metallic Au (at *E* = 0.95 V) catalyzes the oxidation of glucose in the course of 24 h with the following selectivity: 63% GA, 29% FA, 9% glycolic acid, and traces of tartaric acid at a very low current density of 0.23 mA cm^–2^.^69^ In 0.1 M H_2_SO_4_, metallic Au does not show any clear electrocatalytic activity (∼0 mA cm^–2^) for mannose oxidation, similar to the effect of (bi)sulfate adsorption on glycerol oxidation observed on Au in acidic media (see section 4.1.1).^110^ In this case, the competitive adsorption between HSO_4_^–^ and the saccharide diminishes the electrocatalyst activity,^103^ as was also observed for the electrocatalytic oxidation of glycerol.^68^

In neutral conditions (pH = 7, 0.1 M H_2_PO_4_^–^/HPO_4_^2–^), metallic Au (0.95 V), and Au(OH)_3_ (1.35 V) both catalyze the oxidation of glucose at low currents of 0.38 mA cm^–2^ and 0.13 mA cm^–2^, respectively (cyclic voltammetry: 50 mV s^–1^ and 0.2 M glucose).^69^ Under similar reaction conditions and for the electrocatalytic oxidation of glycerol on Au, comparable currents were measured.^54,79^ This indicates that Au is hardly active for catalyzing saccharide and sugar alcohol oxidation under neutral reaction conditions.^69,76^ The onset potential for the electrocatalytic oxidation of saccharides over metallic Au electrodes is 0.4 V,^69^ while for the electrocatalytic oxidation of sugar alcohols it is 0.8 V.^54^ After 6 h at *E* = 0.87 V over an Au electrode, glucose was converted to GA (selectivity = 93%), GLR (7%) and traces of FA, glycolic acid, and tartaric acid.^69^ Under similar reaction conditions and after 8 h of reaction at *E* = 0.87 V over an Au electrode, the products were GA (selectivity = 97%) and GLU (3%). This indicates that Au catalyzes the oxidation of the anomeric carbon with a high selectivity through an oxygenative or dehydrogenative step (i.e., the oxygenative oxidation of the anomeric carbon of glucose results in an adsorbed GA species, while the dehydrogenative oxidation of the anomeric carbon of glucose results in an adsorbed gluconolactone species, which can successively desorb and hydrolyze non-electrochemically to form GA, see Scheme 10).^69^ However, the activity of Au for the electrocatalytic oxidation of glycerol^54,79^ (which proceeds through the oxidation of the primary alcohol group) and glucose^69,76^ do not differ significantly. Moreover, GLU can only be formed through the electrochemical oxidation of GD (Scheme 10), which is an intermediate in the glucose oxidation pathway that is formed by the electrochemical dehydrogenative oxidation of the primary alcohol group of glucose. Therefore, it is likely that Au electrocatalysts can also promote the oxidation of the primary alcohol group of glucose (Scheme 10), resulting in the formation of GD. This might indicate that the formation of GD was overlooked and that Au is therefore much less selective toward GA than what is currently being stated in the literature. Finally, the low selectivity toward C–C cleavage products under neutral conditions is in line with the electrocatalytic oxidation of sugar alcohols (glycerol) over metallic Au (at *E* = 0.8–1.2 V, pH = 7, 0.1 M Na_2_SO_4_), where GALD was formed with 100% selectivity,^54^ while Au(OH)_3_ at *E* > 1.2 V promotes more C–C cleavage reactions (section 4.1.1).^54^ These results indicate that neutral conditions and metallic Au (*E* < 1.2 V) are more favorable than acidic conditions for the selective conversion of saccharides and sugar alcohols.

It is worth noting that the low activity of Au in acidic and neutral conditions can also be correlated to the adsorption of the anions present in the electrolytes used in such cases (e.g., sulfate, phosphate), which have been reported to lead to blocking of the active sites of the electrocatalyst (see also section 2.1.2).^68^ The adsorption of the anions might also affect the selectivity of the electrocatalyst, though this aspect has not been addressed in detail in the literature.

**Figure 12:**
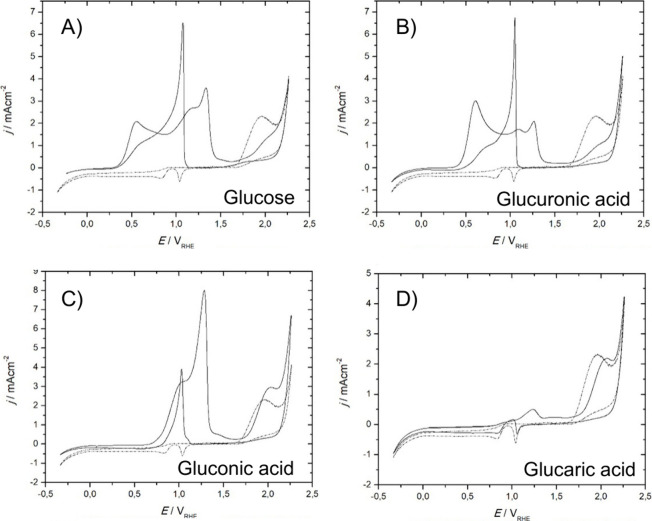
Cyclic voltammetry of a polycrystalline Au electrode in the absence (dashed lines) and presence of (solid lines): (A) 0.04 M glucose, (B) 0.04 M glucuronic acid, (C) 0.04 M gluconic acid, and (D) 0.04 M glucaric acid, measured in 0.1 M NaOH (pH = 13) at 10 mV s^–1^. Adapted with permission from ref (91). Copyright 2020 Wiley-VCH.

At pH = 13 and in the metallic Au region (at *E* < 1.2 V), Figure 12A, B shows that the electrochemical oxidation of glucose, mannose, and glucuronic acid (GLU) over Au electrodes starts at 0.35 V.^28,91,110^ The oxidation reaction at this potential corresponds to the oxygenative/dehydrogenative oxidation of the anomeric carbon (both of glucose and mannose). This low onset potential for the anomeric carbon group was also found for other molecules that bear anomeric carbon groups (xylose, 2-deoxy-d-glucose, d-glucose-6-phosphate, and GLU).^214^ By contrast, Figure 12C shows that the electrochemical oxidation of the C6-OH group of GA and mannonic acid over Au electrodes initiates at 0.75 V,^91,110^ while Figure 12D shows no clear peak for glucaric acid oxidation in the *E* = 0.3–0.85 V range.^91^ The onset potential for the oxidation of the C6-OH group corresponds to the onset potential of other molecules that only contain alcohol groups and no anomeric carbon or aldehyde groups (glycerol, sorbitol, GA, methyl β-d-glucopyranoside, and 1,6-anhydro-β-d-glucose).^54,106,214,224^ Considering the scan rates, the current densities for the electrocatalytic oxidation of glucose measured on Au in alkaline conditions (2 mA cm^–2^, recorded in the presence of 0.04 M glucose at 10 mV s^–1^) are of a 1–2 times higher order of magnitude than under neutral and acidic reaction conditions (0.2 mA cm^–2^, recorded at 50 mV s^–1^ and 0.2 M glucose). A similar trend was observed for sugar alcohol oxidation on Au, which was attributed to the rate-limiting step being affected by the pH (i.e., hydroxide ion concentration) of the electrolyte (Scheme 7). This indicates that the rate-limiting step for the electrocatalytic oxidation of glucose on Au electrodes is determined by either (1) the hydroxide ion concentration at the interface or (2) non-electrochemical reactions that are needed for the formation of the more electroactive enediol (see section 2.1.1).

At pH = 13 and on a polycrystalline Au electrode (Figure 12A, B), an increase in potential results in the electrocatalytic oxygenative/dehydrogenative oxidation of the anomeric carbon of glucose and oxidation of the aldehyde group of GLU at ∼0.3 V with a peak potential at ∼0.5 V.^225^ A further increase in potential results in a second and third peak potential for both molecules at ∼1 and ∼1.25 V vs RHE. By contrast, in Figure 12C only two peak currents were observed for the oxidation of the C6-OH group (GA) on the Au electrode at ∼1 and ∼1.25 V vs RHE, while in Figure 12D only one peak current was observed for the oxidation of GLR at ∼1.25 V vs RHE, which is likely related to C–C cleavage reactions. At higher potentials than 1.25 V vs RHE, Au passivates^91,214^ through the formation of oxidized Au species, possibly in the form of Au(OH)_3_, as was also observed for the electrocatalytic oxidation of sugar alcohols.^54,106^ In the negative-going scan and in the absence of a reactant, a cathodic peak appears at *E* = 1 V (Figure 12A–C, dashed line), corresponding to the reduction of Au oxides/hydroxides to metallic Au. By contrast, in the negative-going scan and in the presence of a reactant, a sharp anodic peak appears at *E* = 1 V (Figure 12A–C, solid line), corresponding to the electrocatalytic oxidation of the reactants. This indicates that once the Au surface oxide is reduced, the oxidation of the organic molecule starts again. Finally, the measured current density and thus the electrocatalytic activity for the oxidation of saccharides over Au was higher for larger molecules going from d-allose to d-erythrose to GALD.^214^ These results suggest that a higher p*K*_a_ of the molecule results in higher catalytic activity,^214^ which matches the results for the electrocatalytic oxidation of sugar alcohols.^79^

**Figure 13:**
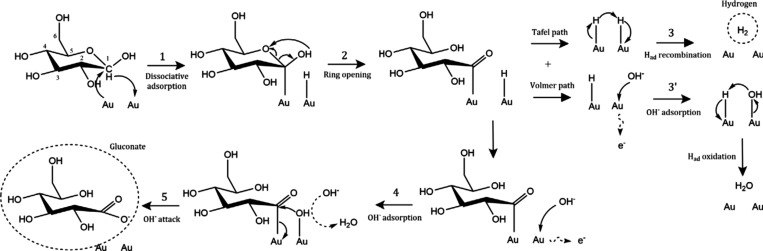
Mechanism proposed by Medrano-Banda et al. for glucose oxidation on Au surface at *E* ≤ 0.7 V and in 0.1 M NaOH. Adapted with permission from ref (226). Copyright 2024 Elsevier.

For long-term electrolysis, at pH = 13, after 65 h and at *T* = 5 °C (to prevent glucose isomerization), metallic Au (at *E* = 0.55 V) promotes the conversion of glucose to GA (selectivity = 87%), FA (6%), and traces of GLR.^91^ The detection of FA confirms that C–C cleavage does happen on Au electrodes already at *E* = 0.55 V.^91^ In comparable studies performed at pH = 10–13 over metallic Au (at *E* < 0.85 V), similar results were obtained, where the oxygenative/dehydrogenative oxidation of the anomeric carbon of glucose is preferred over the electrocatalytic oxidation of the alcohol group.^28,215,216^ This is in line with studies devoted to the electrocatalytic oxidation of sugar alcohols, where the onset potential over metallic Au lies close to 0.8 V.^54,106^ For the electrocatalytic oxidation of glucose, the selectivity toward GA ranged between 86 and 100%, confirming the preference of Au electrodes for the oxygenative/dehydrogenative oxidation of the anomeric carbon.^28,91,215,216^ A reaction mechanism for the oxidation of glucose on the surface of a polycrystalline Au electrocatalyst between *E* = 0.3–0.7 V has been recently proposed by using cyclic voltammetry, Koutecky–Levich analysis, DEMS, FTIRS, and HPLC techniques (Figure 13) at pH = 13.^226^ Under these conditions, a selectivity of 70–100% toward GA was achieved. It was proposed that at low potentials the first step is a dissociative adsorption of glucose which leads to the formation of adsorbed hydrogen (Au-H_ad_). This leads to the formation of H_2_ via the Tafel reaction, which was detected by DEMS.

At pH = 11.3 (0.1 M Na_2_CO_3_), an increase in applied potential from 0.5 to 0.8 V resulted in an increase in glucose conversion from 6 to 30%, while the selectivity for GA (85%) remained unaffected.^28^ At higher potentials (at *E* > 0.85 V), the oxidation of both the anomeric carbon and primary alcohol groups of saccharides (mannose, galactose, and glucose) are promoted over the Au electrode, resulting in the formation of C6-dicarboxylates (C1 + C6 oxidation: mannaric acid, galactaric acid, and GLR) and the formation of C–C cleavage products.^28,91,110,215−217^ As the potential is increased further from 0.85 to 1.35 V, the C–C cleavage reactions are further promoted at the expense of C6-dicarboxylates.^28,110,217^ These results coincide with those of sugar alcohol electro-oxidation, where *E* < 0.8 V over Au electrodes results in minimal C–C cleavage reactions,^57^ while *E* > 0.8 V significantly promoted C–C cleavage reactions.^54,106,107^ There is one outlier in this trend for saccharide oxidation, in which it was shown that the electrocatalytic oxidation of xylose over Au (at *E* = 1.1 V) did not result in the formation of xylaric acid (C5-dicarboxylate) but only 63% xylonic acid.^224^ The lack of detection of C5-dicarboxylates and the effective detection of C–C cleavage products (e.g., 7.4% oxalic acid or 6.2% glycolic acid) at the very same conditions, either suggest a higher preference of C5-monocarboxylate to get degraded (C–C cleavage) rather than oxidized to C5-dicarboxylates, or that C5-dicarboxylates quickly degrade under alkaline conditions.

The selective production of glucaric acid (GLR) was evaluated via a sequential two-step electro-oxidation process.^28^ At pH = 11 (0.1 M Na_2_CO_3_) and *E* = 0.6 V over an Au electrode, GA was selectively produced (84%),^28^ avoiding the non-electrochemical reactions that would be expected at more alkaline conditions (section 2.1.1). The resulting mixture was oxidized for 18 h at *E* = 1.1 V over Au to GLR with 89% selectivity. However, the overall conversion of GA remained very low (2.4%),^28^ as GLR appears to poison the electrocatalyst surface area, independent of the pH of the electrolyte within the assessed alkaline conditions (pH = 11.5–13.5).^28,69^ The Au electrode poisoning due to the adsorption of oxidized organic products can be overcome by applying an alternating potential to refresh the electrode surface.^28,69,110,209,217^ Despite the alternating potential, higher conversions for the successive electrocatalytic oxidation of GA to GLR were not achieved,^28^ in line with what was observed with the electrocatalytic oxidation of sugar alcohols (see section 4.1.2).^159^ Alternatively, a cyclic voltammetry program can be employed to remove oxidized products and reactivate the electrocatalyst.^214^ For instance, galactose was oxidized over an Au electrode at pH = 13 by applying CV for 4 h in the 0.2–0.7 V range, followed by CV for 7 h between 0.8 and 2.0 V. In the lower potential range, the anomeric carbon is mainly converted, while in the higher potential range, both the anomeric carbon as well as the C6-OH group are oxidized, resulting in a selectivity of 35% toward galactonic acid and 23% toward galactaric acid.^214^ Nevertheless, C–C cleavage products such as glycolic, tartaric, and oxalic acids were also observed at the end of the reaction, accounting for 40% of the initial reactant. The use of CV has been shown to overcome Au surface poisoning, as relatively high conversions (80%) were achieved. Despite this, it must be borne in mind that the employed low reactant concentrations (11 mM) may have affected the outcome of the results.^214^

Schlegel et al. investigated the effect of mass transport and electrode potential on the electrocatalytic oxidation of glucose, gluconic acid, and glucuronic acid over Au, using controlled rotation speeds of 0, 900, and 2500 rpm.^227^ They found that a moderate rotation speed of 900 rpm improved both the rate and selectivity for converting glucose to glucuronic acid and glucuronic acid to glucaric acid, while it also improved the selectivity of gluconic acid to glucuronic acid, but did not significantly enhance the reaction rate. This study highlighted how mass transport can play a crucial role in electrochemical processes and is thus an important aspect to be considered in large scale systems.

In conclusion, independent of the pH, Au electrodes have only been reported to catalyze the oxidation of the primary alcohol group and anomeric carbon and not the secondary alcohol groups, in agreement with the results found for the electrocatalytic oxidation of sugar alcohols (section 4.1.1). The activity of Au is highly affected by the pH of the electrolyte, resulting in activities of 1–2 orders of magnitude higher under alkaline conditions than under acidic or neutral conditions. Moreover, the saccharides with a lower p*K*_a_ are more reactive on Au electrodes. This indicates that the rate-limiting step for electrocatalytic oxidation of saccharides on Au is base-promoted, resembling that of sugar alcohol oxidation (section 4.1.1). To evaluate whether the rate-limiting step for the electrocatalytic oxidation of saccharides on Au is base-promoted, a study is required that follows the approach of Kwon et al.^79^ Au was found to be highly selective for the electrocatalytic oxygenative/dehydrogenative oxidation of the anomeric carbon of glucose to GA. Yet, at *E* ≥ 0.75 V the formation of GD should also be considered, since this is the onset potential for the electrocatalytic oxidation of primary alcohol groups. To prove that GD is only formed at *E* ≥ 0.75 V on Au, chronoamperometric measurements on the electrocatalytic oxidation of glucose over Au electrodes need to be conducted between 0.4 to 1 V at pH ≤ 10, and the formed products should be quantified with an analytical technique that enables the separation and quantification of GD.^209^ Finally, under the studied reaction conditions at pH ≤ 13.5, Au was not found to be an effective electrocatalyst for the production of GLR, as the formation of low concentrations of GLR already effectively poisons the catalyst. Alternatively, an increase in pH to 14.3–15 could aid in the production of GLR, similarly to what was shown for the electrochemical oxidation of glycerol to TA and MOA over an Au elctrode.^105,107^ However, this approach is likely to cause significant amounts of retro-aldol reaction products (see section 2.1.1).

#### 5.1.2 Pt-Based Electrocatalysts for the Oxidation of Saccharides

This section discusses the electrochemical oxidation of saccharides at different pH and potentials on Pt electrocatalysts. Among the publications devoted to the electrochemical oxidation of saccharides, Pt has gathered the most attention,^70,109,212^ as it is known as a promising electrocatalyst to perform the oxygenative/dehydrogenative oxidation of the anomeric carbon of glucose.^71^ In contrast to Au, Pt has also been shown to be active in catalyzing the oxidation of the secondary alcohol groups of glucose.^71,209,212^ This section discusses the electrocatalytic oxidation of saccharides, such as glucose, mannose, and xylose, over Pt electrodes.^69,71,91,110,212,224^ Most studies on the selective electrocatalytic oxidation of saccharides have been performed at pH = 13,^71,91,110,212,224^ while only a few studies have discussed the oxidation at neutral conditions^69,119,209^ or acidic conditions.^69^

At pH = 1 (0.1 M HClO_4_), the electrocatalytic oxidation of glucose, fructose, and GA was studied over Pt(111) and Pt(100) electrocatalysts by LSV.^228^ On the one hand, two peaks at *E* = ∼ 0.35 V and ∼0.7 V were observed over Pt(100) for the electrocatalytic oxidation of glucose, while only one peak at *E* = ∼0.5 V was observed over Pt(111). On the other hand, only one peak at *E* = ∼0.7 V was observed for the electrocatalytic oxidation of fructose or GA over Pt(100). This coincides with the behavior observed in the electrocatalytic oxidation of glycerol, as Pt(111) is nearly inactive for catalyzing the oxidation of primary alcohol groups, while Pt(100) can effectively catalyze the conversion of this functional group (section 4.1.2).^22^ Yet, these studies do not investigate in depth the correlation between single-crystal facet of Pt electrodes and the corresponding product selectivity in the conversion of saccharides. Kokoh et al. studied the electrocatalytic oxygenative/dehydrogenative oxidation of glucose in 0.1 M HClO_4_ (pH = 1) on Pt electrodes at *E* = 1.1 V.^69^ After 30 h, glucose was converted to GA (selectivity = >90%), GLU (6%), and some C–C cleavage products (tartaric acid and oxalic acid).^69^ The high selectivity toward GA (2e^–^ oxidation product) is in line with sugar alcohol oxidation in which Pt electrodes at *E* ≤ 1.1 V mainly catalyze the formation of 2e^–^ oxidation products from glycerol, resulting in glyceraldehyde.^56−58^ The formation of saccharides from sugar alcohols (Scheme 6) and GLU from glucose (Scheme 10) requires first the dehydrogenative oxidation of a primary alcohol group. Therefore, it is expected that some GD should also have been formed through the dehydrogenative oxidation of the primary alcohol group of glucose. Potentially, the formation of GD has been overlooked or this compound only constitutes a very minor fraction of the product mixture. Presumably, the high selectivity toward GA can be explained by the higher reactivity of the anomeric carbon of glucose than that of its primary alcohol group. To our knowledge, the selective electrocatalytic oxidation of saccharides over Pt electrodes at *E* < 0.85 V has yet to be studied under acidic conditions.

Under neutral conditions (pH = 7), Pt electrodes at 0.62 V can promote the oxygenative/dehydrogenative oxidation of glucose to produce GA with > 85% selectivity and minor fractions of GLU (8%), oxalic acid, and tartaric acid.^69^ By contrast, under similar reaction conditions a more recent study showed that the selectivity for the oxidation over a Pt electrode is 70% GA, 23% GD, and minor contents of GLU, GUL, and GLR.^209^ Following Scheme 10, these results show that metallic Pt not only catalyzes the oxygenative/dehydrogenative oxidation of the anomeric carbon of glucose but also has high activity toward the dehydrogenative oxidation of the primary alcohol group of glucose, being in line with sugar alcohol oxidation (section 4.1.2).^54^ Under similar reaction conditions, Pt electrodes at *E* = 0.64 V can catalyze the dehydrogenative oxidation of gluconic acid at the primary alcohol group with relatively high selectivity, resulting in selectivities of 86% GUL and 10% GAR. At higher potentials (*E* = 1.2 V) (oxidized) Pt electrodes can catalyze glucose oxidation with high selectivity to GA (91%) and minor contents of GD, 2-k-GA, 5-k-GA, GLU, GUL, and GLR.^209^ The successive oxidation of GA results in selectivities of 70% GUL, 12% 2-k-GA, 6% 5-k-GA, and 9% GLR, which shows that at higher potentials oxidized Pt-based electrodes also catalyze the dehydrogenative oxidation of secondary alcohol groups. In contrast, at lower potentials metallic Pt electrodes were found to be hardly active for catalyzing the oxidation of the aldehyde group of glucuronic acid, thereby showing poor activity for oxygen transfer reactions.^209^ This is also seen for the electrocatalytic oxidation of glycerol on Pt at neutral pH, where at *E* < 1.2 V glyceraldehyde is formed predominantly, while at *E* = 1.2 V glyceric acid is formed more selectively.^54^

At near neutral conditions (pH = 8) and *T* = 50 °C in a paired electrochemical cell for CO_2_ reduction and glucose oxidation with Pt/C as the anode, GA is produced more selectively by increasing the current density, going from 49% FE at 80 mA cm^–2^ to 58% FE at 160 mA cm^–2^.^119^ The increase in FE toward GA was mainly at the expense of GLU, which decreased from 20% FE to a few percent FE. At 80 and 160 mA cm^–2^, the cell potential was ∼1.7 and ∼2.3 V, respectively, resulting in the competition between oxygen evolution reaction (OER) and the electrocatalytic oxidation of glucose. However, the sluggish nature of OER only resulted in minor rates of oxygen production, limiting the FE toward O_2_ below 3%, independent of the current density.^119^ The increase in selectivity toward GA at higher potentials might be related to the oxidation state of the Pt electrode.^209^ This indicates that it might be relevant to study the electrocatalytic oxidation of saccharides and sugar alcohols at potentials above 1.5 V in the region where competition with OER may be expected to give industrially-relevant current densities (> 100 mA cm^–2^).^119^

**Figure 14:**
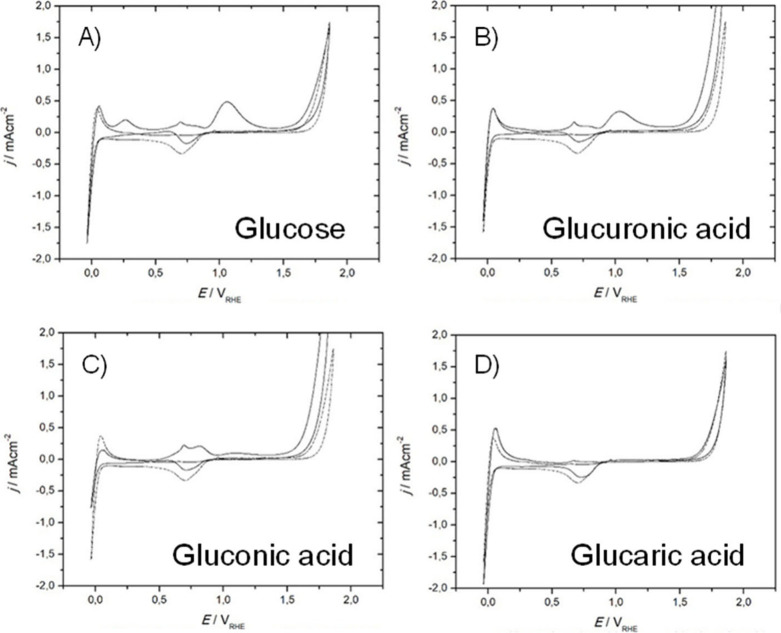
Cyclic voltammetry of a polycrystalline Pt electrode in the absence (dashed lines) and presence of (solid lines): (A) 0.04 M glucose, (B) 0.04 M glucuronic acid, (C) 0.04 M gluconic acid, and (D) 0.04 M glucaric acid, measured in 0.1 M NaOH (pH = 13) at 10 mV s^–1^. Adapted with permission from ref (91). Copyright 2020 Wiley-VCH.

Under alkaline conditions in 0.1 M NaOH (pH = 13), the electrocatalytic oxidation of glucose, mannose, and xylose and their oxidation products were studied by CV (see Figure 14) and CA.^71,91,110,212,224^Figure 14A andC show a typical CV of Pt in the presence of glucose or GA in 0.1 M NaOH, which are very similar to the CVs in the presence of mannose and gluconic acid.^91,110,212^ This indicates a similar reactivity of functional groups for different reactants. The first peak at *E* = ∼ 0.3 V in the presence of glucose and mannose is attributed to the dehydrogenation of the anomeric carbon, which does not require hydroxide ions and was not observed for the sequential oxidation products.^91,110^ In this reaction, following Scheme 10, an adsorbed lactone is formed, which can desorb from the surface and react non-electrochemically with water to form a monocarboxylate such as GA or mannonic acid. At *E* = 0.5 V, the current gradually increases again in the presence of glucose, gluconic acid, and glucuronic acid, while no increase in current is observed for GLR.^91^ This indicates that higher potentials are required for the catalytic dehydrogenation of gluconic acid at the primary alcohol group (Scheme 10) and the electrocatalytic oxidation of the aldehyde group of GLU compared to the dehydrogenation of the anomeric carbon of glucose. As soon as the Pt surface becomes oxidized (at *E* = ∼ 0.8 V), the current starts to decrease with increasing potential up to *E* = ∼ 0.9 V, after which it increases again to a maximum (*E* = 1.15–1.2 V). When PtO_2_ (at *E* > 1.2 V) is expected to become the dominant surface species, the current quickly drops again, indicative of the low activity of PtO_2_. Interestingly, at *E* = 0.75 V (where the Pt surface should still be mainly metallic), higher current densities and thus reaction rates for the electrocatalytic dehydrogenative oxidation of the C6-OH group of GA were observed than on a more oxidized surface (*E* = 1.15–1.2 V).^91,212,229^ This follows the trend for the electrocatalytic oxidation of mannonic acid,^110^ fructose,^109^ and glycerol at pH = 13^54,106^ and 1-O methyl glucoside (e.g., glucose with a protecting group at the anomeric carbon) at pH 10.^230^ Moreover, Moggia et al. showed that the aldehyde group of glucuronic acid is more easily oxidized at *E* = 0.75 V than at *E* = 1.15–1.2 V,^91^ confirming the results obtained by van der Ham et al. for the electrocatalytic oxidation of GLU at pH = 7.^209^ All this strongly indicates that metallic Pt favors dehydrogenation reactions, while PtO_*x*_ promotes oxygen transfer reactions.

The electrocatalytic oxidation of galactose was studied at pH = 13 (0.1 M NaOH) on a Pt electrode at *E* = 0.25 V.^217^ This approach resulted in 76% conversion and yielded 34% galactonic acid, 1% galactaric acid, and 24% of C–C cleavage products, composed mainly of glycolic acid and FA. CV scans were performed in the presence of galactose to evaluate the catalyst activity over time. It was found that the activity decreased drastically over time, indicative of strong poisoning of the Pt surface.^217^ This shows that metallic Pt can already catalyze the oxygenative/dehydrogenative oxidation of the anomeric carbon at very low potentials but also induces significant amounts of C–C cleavage reactions.

Under alkaline conditions in 0.1 M NaOH (pH = 13), the electrochemical oxidation of glucose was studied on Pt electrodes at *E* = 0.7 V^71,91^ and at *E* = 1.1 V.^91^ Kokoh et al. showed that at *E* = 0.7 V, a conversion of 63% for glucose can be achieved in 10 h, with a selectivity of 64% toward GA, 2% toward 5-k-GA, 1% toward 2-k-GA, and 1% toward GLR.^71^ By contrast, Moggia et al. showed that at *E* = 0.7 V glucose can be converted (the amount was not given) over a Pt electrode giving the following product selectivity after 64 h: 68% toward GA and 13% toward GLR.^91^ The higher selectivity toward GLR can potentially be explained by the longer reaction time, resulting in products with a higher degree of oxidation. In a sequential study performed by Kokoh et al., the electrocatalytic oxidation of GA was studied at pH = 13 over a Pt electrode at *E* = 0.7 V.^212^ After 10 h of electrolysis, 12% GA was converted with a FE of 95% to a large variety of products, with the following selectivity: 14% 5-k-GA, 11% GLU (following Scheme 10, this is likely to be GUL), 6% GLR, and 32% C–C cleavage products.^212^ These results show that the oxidation of the C5-OH is most likely achieved through the oxidation of GA. Yet, the formation of 5-keto gluconic acid could also originate from the isomerization of glucuronic acid under alkaline conditions (see section 2.1.1). Moggia et al. also showed that at *E* = 1.1 V Pt electrodes can catalyze the oxidation of glucose after 64 h to the following product selectivity: 78% GA and 6% GLR.^91^ GLR is either formed through the oxidation of GLU or GUL at the aldehyde group via an oxygen transfer reaction (Scheme 10).^209^ The lower selectivity of Pt electrodes toward GLR at higher potential can be explained by the high selectivity toward GA of the PtO/PtOH species that are expected to be present at the surface of the Pt electrode at such potential and their ability to successively dehydrogenate GA at the secondary alcohol groups, resulting in higher selectivity toward 2-k-GA and 5-k-GA, and lower selectivity toward GLR.^71,209^

Under alkaline conditions in 0.1 M NaOH (pH = 13) at *E* = 1.0 V over a Pt electrode, 26% xylose was oxidized to yield the following product distribution: 19% xylonic acid and 48% C–C cleavage products (33% of the products were not analyzed).^224^ These results show once more that xylose is more easily degraded to smaller molecules than galactose or glucose.^91,217,224^

Under harsh alkaline conditions in 1 M KOH (pH = 14) at *E* = 1.3 V over Pt electrodes (with Pt species at the surface expected to be in oxidized state), the oxidation of glucose resulted in 67% glucose conversion after 18 h and yielded 42% GA and 20% GLR.^127^ Interestingly, under these harsh alkaline conditions no degradation products were observed, contrasting other studies and our discussion in section 2.1.1.^86,87,95^ Nonetheless, the relatively high selectivity toward GLR (i.e., a dicarboxylate) indicates that dicarboxylates can only be formed at highly alkaline conditions, as was also shown for glycerol oxidation on Pt and Au (section 4.1.1 and 4.1.2).^105,107,113,160,161^

In conclusion, Pt(111) does not show appreciable activity for catalyzing the oxidation of primary alcohol groups, while Pt(100) can effectively catalyze the oxidation of this functional group. GLU is frequently reported as product but not its precursor GD (Scheme 10), indicating that the formation of GD might have been overlooked. GD is likely one of the main products on metallic Pt, since metallic Pt promotes dehydrogenation reactions. By contrast, PtO_*x*_ is active in catalyzing oxygen transfer reactions (indirect mechanism), thereby promoting the formation of GA. Additionally, in contrast to metallic Pt, PtO_*x*_ promotes the successive oxidation of the secondary alcohol groups of GA, resulting in the formation of 2-k-GA and 5-k-GA and thus more complex reaction mixtures. The formation of GLR only seems to be feasible under harsh alkaline conditions (pH ≥ 14).

#### 5.1.3 Ru-Based Electrocatalysts for the Oxidation of Saccharides

The electrocatalytic oxidation of glucose on platinum group metals other than Pt has only been studied on Ru-based electrocatalysts in harsh alkaline conditions (pH = 14, 1 M KOH) at *E* = 1.3 V.^127^ After 18 h, 90% glucose was converted to 52% GA and 28% GLR.^127^ These harsh alkaline conditions did not cause the degradation of the reactant or products,^127^ opposing other studies and our discussion in section 2.1.1.^86,87,95^ The relative high selectivity toward GLR can potentially be attributed to the high alkalinity of the electrolyte, which was also found to be crucial for the electrocatalytic oxidation of glucose on Pt and the electrocatalytic oxidation of glycerol on Pt and Au.^105,107,113,160,161^

#### 5.1.4 Ni- and Co-Based Electrocatalysts for the Oxidation of Saccharides

This section evaluates the electrochemical oxidation of saccharides on Ni-and Co-based electrocatalysts at different pH and potentials. Ni was used to study the electrocatalytic oxidation of glucose and xylose at pH = 11^231^ and mannose and galactose at pH = 13,^110,217^ while Co was only used to study the electrocatalytic oxidation of glucose at harsh alkaline conditions (pH = 13.7).^99,232,233^

At pH = 11, the electrocatalytic oxidation of glucose and xylose was studied on a Ni-based electrode consisting of NiO (as determined by *ex situ* XRD and XPS).^231^ The electrocatalytic oxidation of glucose and xylose was performed at *E* = 1.44 V.^231^ At this potential, NiOOH is expected to be present at the surface of Ni-based electrodes,^169^ coinciding with the potential at which the indirect oxidation mechanism is dominant.^169^ The electrocatalytic oxidation of glucose resulted in a selectivity of ∼60% toward GA, ∼10% toward GLR and ∼27% toward C–C cleavage products (∼equimolar amounts of oxalic acid and tartaric acid).^231^ The successive electrocatalytic dehydrogenative oxidation of the primary alcohol group of GA to form GUL (mistaken by GLU in the article, see Scheme 10) could only be achieved effectively with the aid of TEMPO.^231^ The successive oxidation of the aldehyde group of GLU could be achieved selectively with Ni-based electrodes, reaching ∼85% GLR yield.^231^ This approach that combines Ni-based electrodes and TEMPO also resulted in a high selectivity for the electrocatalytic oxidation of xylose to xylaric acid. Following Scheme 10, this indicates that the indirect oxidation mechanism of β-NiOOH can effectively lead to the oxidation of aldehyde groups and oxygenative/dehydrogenative oxidation of the anomeric carbon of glucose and less effectively the dehydrogenative oxidation of the primary alcohol group of glucose.^231^ This contrasts with the results for the electrocatalytic oxidation of glycerol at pH = 11 and *E* = 1.48 V, where glycerol was selectively dehydrogenated at the secondary alcohol group resulting in DHA as the main product.^169^ This discrepancy can tentatively be explained by (1) the higher reactivity of glucose at the anomeric carbon group than its secondary carbon groups, thereby promoting the formation of GA over β-NiOOH and (2) the higher activity of TEMPO for catalyzing the dehydrogenative oxidation of the primary alcohol group of GA than that of β-NiOOH for catalyzing the oxidation of the secondary alcohol group of GA, thereby promoting the formation of GUL by TEMPO.

**Figure 15:**
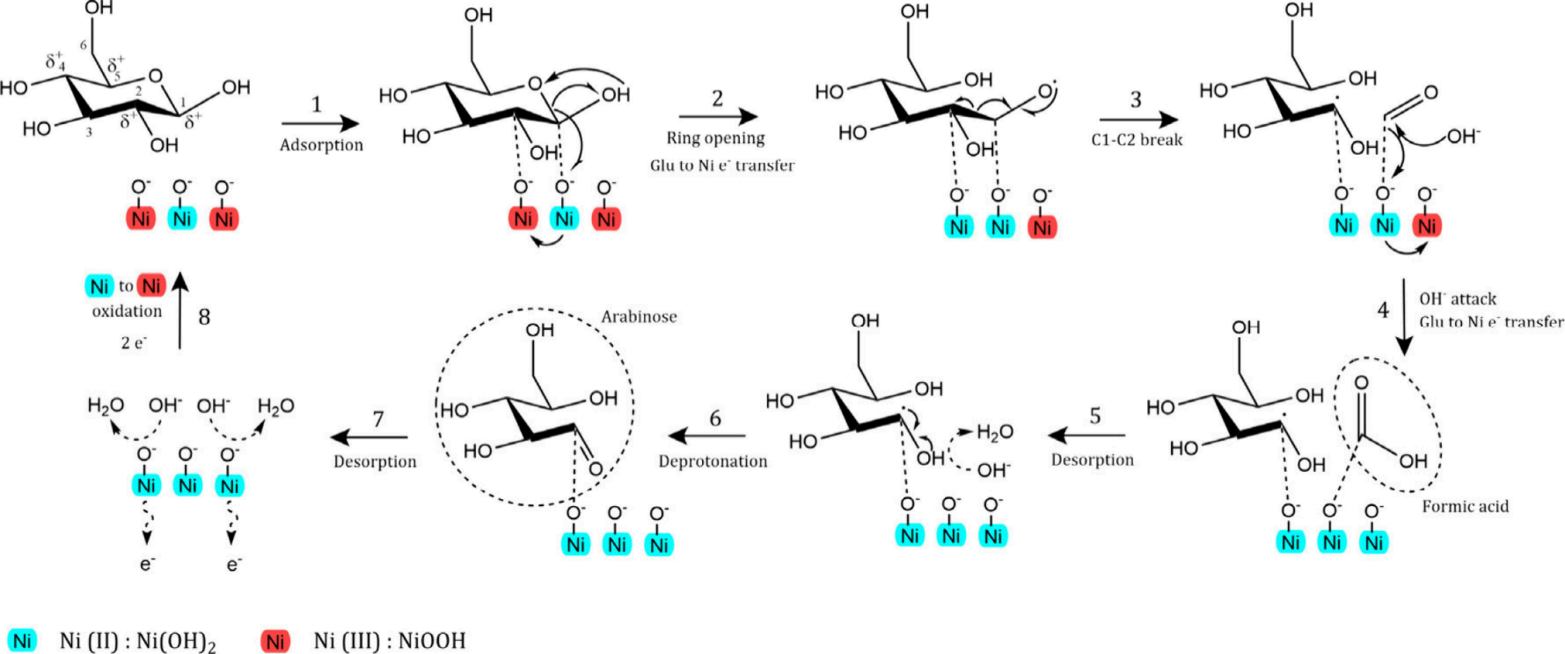
Mechanism proposed by Medrano-Banda et al. for glucose oxidation on Ni(OH)_2_/NiOOH surface at *E* ≥ 1.2 V in 0.1 M NaOH. Adapted with permission from ref (226). Copyright 2024 Elsevier.

At pH = 13, the electrocatalytic oxidation of glucose and other saccharides (mannose and galactose) was only feasible on Ni electrodes in the β-NiOOH region (at *E* > 1.2 V).^110,217^ CV tests with glucose and GA showed that the latter can be oxidized at potentials lower than the oxidation of glucose itself (*E* ≈ 1.1 V), meaning that on Ni electrodes it is not possible to selectively generate gluconic acid since it readily oxidizes to other products.^226^ The main products observed via HPLC were arabinose and formic acid (*E* = 1.47–1.6 V), which proves that Ni induces breaking of C–C bonds. Additionally, the arabinose/FA molar ratio does not match, what hints toward the cleavage of glucose/arabinose molecules to other smaller molecules than FA. A similar trend toward C–C bond cleavage was observed for both the electrocatalytic oxidation of galactose (at *E* = 1.6 and 2.3 V) and mannose (at *E* = 2.3 V), which were studied in competition with the OER. For galactose, it was shown that a higher potential resulted in a lower conversion, which can be attributed to the competition with the OER.^217^ An increase in potential from 1.6 to 2.3 V resulted in an increase in selectivity toward the oxygenative/dehydrogenative oxidation of the anomeric carbon of galactose^234^ yielding 7% and 8.5% galactonic acid, respectively.^217^ For mannose, the electrocatalytic oxidation at *E* = 2.3 V only resulted in 8% selectivity toward mannonic acid.^110^ The high rate of C–C cleavage reactions was attributed to the presence of β-NiOOH at the electrode surface, consisting of a mixture of Ni hydroxide and Ni oxide in equal proportion, with the H atom delocalized between the two species.^217^ The O atoms of these two adjacent surface species have been proposed to interact with two adjacent carbon atoms, thus weakening the C–C bond and promoting the C–C bond cleavage,^217^ in a similar way to the mechanism for C–C cleavage reactions on Pt oxide (Scheme 8). This mechanism has yet to be proven as the applied reaction conditions also promote non-electrochemical reactions, such as oxidative C–C cleavage reactions and the oxidation of aldehydes (section 2.1.1), thereby promoting the formation of FA. However, a proposal for the mechanism through which the electrocatalytic oxidation of glucose on Ni(OH)_2_/NiOOH surface at *E* ≥ 1.2 V in 0.1 M NaOH proceeds has been made following the aforementioned steps (Figure 15).^226^ At higher potentials, the surface is expected to be fully covered with NiOOH and this likely changes the mechanism, resulting in an Eley–Rideal mechanism in which NiOOH reacts directly with glucose from the solution.^234^

The harsh alkaline conditions (pH = 13.7) used to study the electrocatalytic oxidation of glucose on Co-based electrodes^99,232,233^ make it complicated to distinguish between electrochemical and non-electrochemical reactions. At 10 mA cm^–2^ (cell voltage 1.46 V^99^ and 1.34 V^232^), glucose was oxidized with relatively high selectivity toward LA (45–55%), with some formation of FA (5–15%) and minor contents of GA (1.5%) and GLR (1.5%). The formation of LA is likely to have occurred on-electrochemically (see section 2.1.1),^87,99^ while the Co-based electrode only catalyzes the minor fraction of glucose conversion yielding GA and GLR. By contrast, at *E* = 1.3–1.7 V over a Co-based electrode (with μ_1_-OH-Co^3+^ and μ_2_-O-Co^3+^ as proposed active species based on characterization by EXAFS), FA (FE ≥ 60%) and GA (∼25%) were predominantly produced.^233^ The formation of FA indicates that Co-based electrodes effectively catalyze C–C cleavage reactions since these products cannot solely be formed by non-electrochemical reactions (see section 2.1.1).

#### 5.1.5 Cu- and Mn-Based Electrocatalysts for the Oxidation of Saccharides

This section evaluates the electrochemical oxidation of saccharides on Cu- and Mn-based electrodes at different pH and potentials. The electrocatalytic oxidation of glucose was studied at pH = 13 on Cu-based electrodes (*E* = 0.84–1.80 V, under which conditions Cu is expected to be in the oxidized state at the surface of the electrode).^91,215^ The electrocatalytic oxidation of glucose and glucuronic acid starts at 0.84 V, while the electrocatalytic oxidation of gluconic acid and glucaric acid is strongly promoted at *E* = 1.3 V.^91^ This indicates that the electrocatalytic oxygenative/dehydrogenative oxidation of the anomeric carbon of glucose and the electrocatalytic oxidation of the aldehyde of GLU can selectively be achieved at *E* < 1.3 V.^91^ This hypothesis was tested by chronoamperometric measurements on a Cu-based electrode at *E* = 0.84, 1.11, and 1.80 V. At *E* = 0.84 V, where CuO is expected to be present (though not proven by characterization),^91^ and 1.11 V, where solubilized Cu^II^ species originating from CuO were proposed to be present, glucose was mainly oxidized to GLR (selectivity = 38–27%) and GA (30–45%). By contrast, at *E* = 1.80 V, where solubilized CuO_2_^2–^ species are expected to be present, the main product was FA (54%). The high selectivity toward GLR shows that the Cu-based electrode can effectively catalyze the oxidation of the primary alcohol group of GA, which was not deducible from the CV experiments.^91^ Moreover, the high selectivity toward FA at *E* = 1.80 V indicates that the Cu-based electrode is a good electrocatalyst for C–C cleavage reactions at sufficiently high potentials.^91,215^

A Cu-based electrode at *E* = 1.46–1.56 V was also used to study the electrocatalytic oxidation of glucose at harsh alkaline conditions (pH = 13.7).^235^ This study aimed at maximizing LA production. Undoubtably, the harsh alkaline conditions induce non-electrochemical production of LA (see section 2.1.1). The control experiment without a Cu-based electrode showed a relatively similar product distribution as the experiments in which such electrode was applied. Nonetheless, it was claimed that solubilized divalent Cu species can promote C–C cleavage at the C3–C4 bond promoting the formation of GALD and DHA, which are important intermediates for the production of LA.^235^ Wang et al. investigated how the electrocatalytic properties of CuO-derived materials affect the oxidation of glucose, glycerol, and HMF in 1.0 M KOH.^236^ Using a combination of electrochemical and spectroscopic methods, they observed a potential-dependent structural evolution in CuO, transitioning through Cu(OH)_2_ and CuOOH phases as the applied potential increased. Independent of the reactant, it was shown that Cu(OH)_2_ is more effective at catalyzing aldehyde oxidation, while CuOOH shows faster kinetics in alcohol/aldehyde oxidation and carbon–carbon bond cleavage.^236^ Yet, the formation of C6 oxidation products was not reported, meaning that this CuO-based electrocatalyst mainly promotes C–C cleavage reactions.

A MnO_2_/Ti 3D anode was used for the electrocatalytic oxidation of glucose in a flow-cell reactor operated at 3 mA cm^–2^ and mild pH conditions (pH = 2–10),^27^ thus minimizing the isomerization of glucose (see section 2.1.1).^50^ Increasing the pH from 2 to 10 only increased the glucose conversion from 90% to 93%. At pH = 7, the selectivity toward GA (49%) and GLR (45%) was the highest. The high activity of the MnO_2_/Ti electrode was attributed to its high stability under all pH conditions and the fast removal of oxidized products due to the supply of fresh electrolyte and reactant. Moreover, it was argued that good control of the pH is required, since alkaline conditions promote unwanted non-electrochemical reactions (see section 2.1.1), while acidic conditions hamper the formation of GA and GLR. Under optimized reaction conditions (retention time, initial reactant concentration, temperature, pH, and MnO_2_ loading), ∼100% glucose was converted to 85% GLA and 15% GA, yet the Faradaic efficiency remained low (FE = 37%) as most of the current was used for water oxidation (OER).^27^ An ytterbium-doped MnO_2_ electrodeposited on carbon paper (Yb-MnO_2_/CP) was used to study the electrocatalytic oxidation of glucose at pH = 0.7.^237^ On the basis of DFT calculations, it was suggested that the Yb atoms promote the adsorption and desorption process of alcohols and aldehydes on MnO_2_, thereby improving the intrinsic activity of the catalyst while reducing the competing OER. After 3 h of glucose conversion at *E* = 1.47 V, Yb-MnO_2_/CP was able to catalyze the oxidation of 98% 0.1 M glucose to 85% GLR.^237^ These promising results could not be compared with the electrocatalytic oxidation of glycerol over MnO_2_, since the study on glycerol used a borax electrolyte (see section 2.1.1).^178^ Nonetheless, both studies on glycerol oxidation over MnO_2_ show that high selectivity toward GLR without inducing severe amounts of C–C cleavage reactions can be achieved under acidic conditions,^178,237^ thereby opening new routes for the production of glucaric acid.

### 5.2 Noble Bimetallic Electrocatalysts for the Oxidation of Saccharides

Bimetallic formulations in electrocatalysts are generally used to improve the performance compared to monometallic electrocatalysts and to decrease the utilization of scarce precious metals. The structure of this section follows the same order as the section on glycerol oxidation on noble bimetallic catalysts (section 4.2), where Ag-X^216^ (section 5.2.1) is discussed first followed by Au-X (section 5.2.2).^29,114,164,213,216,238−241^ Pt has only been alloyed with Au and was therefore not discussed in a separate section.

#### 5.2.1 Bimetallic Ag-Noble Metal Electrocatalysts for the Oxidation of Saccharides

Only Tominaga et al. studied the electrocatalytic oxidation of glucose on Au_*m*_Ag_100–*m*_-NPs (gold silver nanoparticles).^216^ The synthesized electrocatalysts are composed of either alloys or phase-segregated structures. In this regard, it was observed that AuAg phase-segregated structures had the same onset potential as Au nanoparticles for catalyzing glucose oxidation, while AuAg alloyed structures had a 0.1 V lower onset potential.^216^ This 0.1 V lower onset potential was also observed for the electrocatalytic oxidation of glycerol on alloyed Ag-noble metal (Au,^179^ Pt,^182^ and Pd^183^) electrocatalysts. GA was formed at 0.65–0.7 V with a ∼100% Faradaic efficiency (quantified by HPLC) by using Au-NPs and AuAg-NPs.^216^ At higher potentials (*E* = 1.25 V), the selectivity of AuAg-NPs increased toward C–C cleavage products with increasing silver content.^216^ These results partially contrast the studies performed on the electrocatalytic oxidation of glycerol, where the addition of Ag to Au,^179^ Pt,^182^ and Pd^183,184^ promoted C–C cleavage reactions independent of the applied potential. This difference can tentatively be explained by the lower potential required to oxidize the anomeric carbon group of glucose than the primary alcohol group of glycerol (see section 5.1.1). This would also explain the high selectivity obtained toward GA on AuAg at lower potentials.

#### 5.2.2 Bimetallic Au-Noble Metal Electrocatalysts for the Oxidation of Saccharides

Au has been combined most frequently with other noble metals: Ag,^216^ Pt,^213,240,241^ and Pd.^29,164,239^ Most of the studies on bimetallic Au-noble metal catalysts were performed to evaluate the electrocatalyst activity, while few also report the selectivity.^29,213,216,239^ It is worth mentioning that all the papers in this section use 0.1–1.0 M NaOH/KOH electrolytes, and no studies were performed at pH < 13.

Combining Au with Pt was found to have a positive influence on the electrocatalytic oxidation of glucose at pH ≥ 13, resulting in higher electrocatalytic activities (i.e., higher currents measured by CV) and higher resistance to poisoning in the long term electrolysis.^240,241^ AuPt nanoparticles on reduced graphene oxide (AuPt/rGO) were tested in 0.1 M KOH (pH = 13) in a batch cell and a cell equipped with an anion-exchange membrane (AEM) for the electrocatalytic oxidation of glucose.^213^ At *E* = 0.65 V, Au_50_Pt_50_/rGO catalyzed the production of gluconic (GA) and glucuronic acid (GLU), while no GLR was found (quantified by high-performance liquid ionic chromatography). This reveals that the electrocatalyst can promote (1) the oxygenative/dehydrogenative oxidation of the anomeric carbon of glucose and (2) the dehydrogenative oxidation of the primary alcohol of glucose (see Scheme 10). In the AEM cell, 90% GA was formed with a 65% FE,^213^ indicating that the oxygenative/dehydrogenative oxidation of the anomeric carbon of glucose was the favored step under these conditions. This resembles the selectivity of Au electrodes at *E* < 0.75 V (see section 5.1.1), which are not able to catalyze the dehydrogenative oxidation of the primary alcohol group of glucose at this potential.^28,69,91,110^ Only 10% GLU was formed, which is likely attributed to Pt as it can catalyze the dehydrogenation of C6-OH of glucose at *E* = 0.65 V (see section 5.1.2).^91^ After ∼10 min, the produced GA and GLU amounts are almost equimolar; after ∼10 min the GLU concentration remains constant, while that of GA increases linearly over time. The formation of GLU shows that glucose dialdehyde should have been formed and might therefore have been overlooked (see introductory discussion of section 5). Moreover, the formation of glucose dialdehyde from glucose is expected since PtAu (at *E* = 0.45 V) can catalyze the dehydrogenative oxidation of the primary alcohol group of glycerol to form glyceraldehyde.^81^

At pH = 13, a PdAu electrode was found to be highly active and selective for catalyzing the oxidation of xylose and glucose.^29,238,239^ These studies were performed on glucose and xylose individually^29,238^ and mixtures of these two saccharides.^239^ From chronoamperometric measurements (at *E* = 0.4 V), the effect of Pd_30_Au_70_/C electrocatalyst poisoning was hardly observed after 6 h of electrolysis, and the electrocatalytic activity was restored by the introduction of fresh solution with new reactants.^29^ Under these conditions, Pd_30_Au_70_/C catalyzes the conversion of 67% glucose or 12% xylose with a high selectivity toward GA (95%) and xylonate (>99%), respectively.^29^ The effect of the cell potential was also studied for the electrocatalytic oxidation of glucose and xylose mixtures over Pd_30_Au_70_/C.^239^ After 6 h of electrolysis, it was shown that an increase in cell potential from 0.4 to 0.6 V improves the conversion from 55 to 85% (90–10% glucose-xylose mixture) and from 35 to 65% (50–50% glucose-xylose mixture).^239^ In contrast to the results reported by the same group in 2022,^239^ in 2020 Rafaïdeen et al. showed that an increase in cell potential from 0.4 to 0.8 V results in a decrease in Faradaic efficiency.^238^ The loss in FE was attributed to the formation of C–C cleavage products,^238^ which might be generated either by C–C bond cleavage reaction induced by the electrocatalyst or through retro-aldol reactions (section 2.1), which successively compete in the reaction on the electrocatalyst surface. The formation of GLU was not reported, indicative that the electrocatalytic oxidation of the primary alcohol group of glucose does not take place.

**Figure 16:**
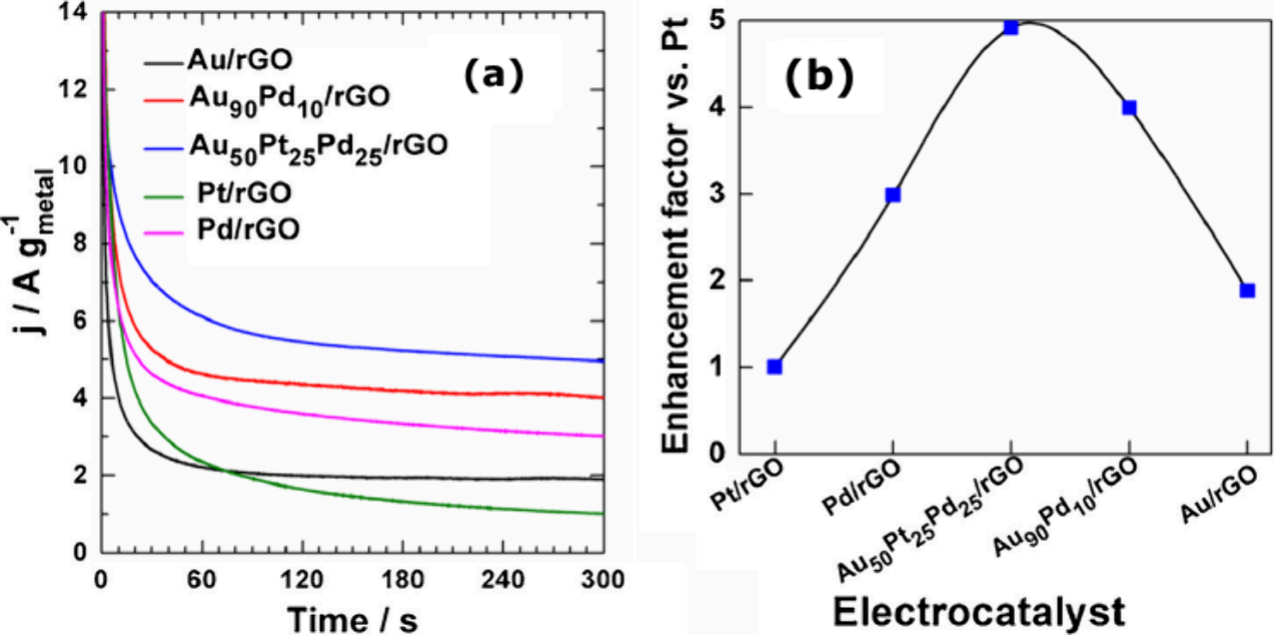
(a) Chronoamperometry experiments in 0.1 mol L^–1^ NaOH + 10 mmol L^–1^ of glucose at 0.6 V vs RHE and (b) corresponding Volcano plot using the electrocatalyst composition as the descriptor of the catalytic performance, from the steady-state current density after 300 s. Reprinted with permission from ref (114). Copyright 2016 Elsevier.

Finally, Au, Pt, Pd, AuPd, and AuPdPt nanomaterials supported on reduced graphene oxide (rGO) were tested as glucose oxidation electrocatalysts in 0.1 M NaOH to evaluate the effect of alloying.^114^ The prepared electrocatalysts were characterized by XRD showing that the bi- and trimetallic systems formed alloyed particles. Chronoamperometric measurements at 0.6 V vs RHE (Figure 16A) show that the Pt/rGO loses its activity over time, while the other electrocatalysts reached a steady state, with Au_50_Pt_25_Pd_25_ and Au_90_Pd_10_ displaying the highest activity (Figure 16B). It was suggested that Pt/rGO deactivates due to the strong adsorption of poisoning intermediates on its surface. For Au_50_Pt_25_Pd_25_, it was found from FTIR data that CO_2_ was formed, caused by C–C cleavage, but CO could not be detected. Even during chronoamperometric measurements at 0.6 V vs RHE, no CO was detected, probably due to the effectiveness of the electrocatalyst to oxidize the adsorbed CO, thus circumventing the poisoning of the electrocatalyst surface.

Whereas most electrocatalysts investigated for the oxidation of glucose are mono- or bimetallic, there are also a few reports on trimetallic systems based on PdPtAu, as the one just discussed.^114^ In another study on trimetallic systems, PdPtAu (1:1:1) supported on carbon was studied as electrocatalyst for glucose oxidation in 0.5 and 1.0 M KOH medium and compared to bimetallic PdPt (4:1) supported on the same type of carbon.^114^ Both electrocatalysts were found to be active in the oxidation of glucose, with the trimetallic system reaching slightly higher anodic current density. When tested in a direct glucose fuel cell, both electrocatalysts produced a peak power density of 0.52 mW cm^–2^.

To conclude, AuPt electrocatalysts are likely to catalyze both the oxygenative/dehydrogenative oxidation of the anomeric carbon and the dehydrogenative oxidation of the primary alcohol of glucose. However, the oxygenative/dehydrogenative oxidation of the anomeric carbon of glucose is favored, resulting in relative high selectivity toward GA. By contrast, AuPd electrocatalysts were only able to catalyze the oxygenative/dehydrogenative oxidation of the anomeric carbon of glucose and xylose, thereby promoting the formation of GA and xylonate. Moreover, an increase in cell potential improves the conversion but also promotes C–C cleavage reactions, thereby decreasing the Faradaic efficiency of the electrocatalysts toward the desired larger molecules.

### 5.3 Noble-Non-Noble Bimetallic Electrocatalysts for the Oxidation of Saccharides

The effect of alloying Au with Cu for the electrocatalytic oxidation of glucose was assessed at 0.1 M NaOH.^215^ The linear sweep voltammetry recorded in the presence of glucose for the alloyed AuCu electrocatalyst resembled that of monometallic Au.^215^ On AuCu electrodes at *E* = 0.65–0.7 V, GA was formed with ∼100% Faradaic efficiency, while at *E* = 1.25 V, AuCu electrodes catalyzed the production of GA (FE = 35%), 2-k-GA (3%), GLR (10%), and large amounts of C–C cleavage products.^215^ The high selectivity of AuCu electrodes at *E* = 0.65–0.7 V toward GA can be attributed to the high selectivity of Au toward the oxygenative/dehydrogenative oxidation of the anomeric carbon of glucose and its inability to catalyze the primary alcohol group of glucose at this potential (section 5.1.1)^91,215^ and the inactivity of Cu for catalyzing glucose oxidation at this potential (section 5.1.5).^91^ This shows that AuCu electrocatalysts might be a valuable option for the oxidation of saccharides to value-added products under alkaline conditions, especially considering that the use of Cu can allow decreasing the amount of scarce and expensive Au.

### 5.4 Noble-Post-Transition Bimetallic Electrocatalysts for the Oxidation of Saccharides

#### 5.4.1 Pt-Post-Transition Bimetallic Electrocatalysts for the Oxidation of Saccharides

Pt has frequently been combined with post-transition metals to study the electrocatalytic oxidation of saccharides. The most common approach is by using adatoms^69,71,110,212,217,230^ and less commonly by alloying.^242^ The Pt-post-transition bimetallic electrocatalysts were used to study the selectivity toward the oxidation of different saccharides, namely glucose,^69,71,212,230^ mannose,^110^ and galactose.^217^ These studies were almost all performed under alkaline conditions(pH = 13)^71,110,212,217,242^ and seldomly under mild alkaline (pH = 10)^230^ and acidic (pH = 1) conditions.^69^ The post-transition metals that have been researched are Bi,^69,230,242^ Pb,^69,71,110,212,217^ and Tl.^69,230^

Pt was modified with Bi, Pb, and Tl adatoms to study the electrocatalytic oxidation of glucose under acidic conditions (pH = 1, 0.1 M HClO_4_).^69^ The effect of adatoms was investigated at various potentials, being 0.6 V for Tl, 0.7 V for Pb, and 1.0 V for Bi. At *E* = 1 V, Bi did not affect the selectivity of Pt.^69^ This can be attributed to the fact that Bi is oxidized and desorbs at these potentials (see section 4.4), thereby losing its effect on the electrocatalyst selectivity, as was also shown for glycerol oxidation.^53,55^ At pH = 1, the addition of Pb adatoms to Pt did not appear to modify the selectivity of Pt significantly, resulting in high contents of GA and minor fractions of GLU, GLR, and C–C cleavage products.^110^ This minor effect of Pb adatoms on Pt electrocatalysts was also shown for the oxidation of glycerol.^200^ Nonetheless, Pb adatoms did show to promote the dehydrogenative oxidation of the secondary alcohol group of glycerol, which was not shown for glucose oxidation, suggesting that some products might have been overlooked (see section 5). The addition of Tl adatoms to Pt resulted in a high selectivity toward GA (96%) by largely suppressing the formation of C–C cleavage products (4% FA) and GLR.^69^ This indicates that the addition of Tl to Pt strongly improves the electrocatalyst selectivity toward the oxygenative/dehydrogenative oxidation of the anomeric carbon group. However, the formation of glucose dialdehyde through the dehydrogenative oxidation of the primary alcohol group of glucose or 2-k-GA and 5-k-GA through the dehydrogenative oxidation of the C2-OH and C5-OH group of glucose might have been overlooked (see section 5). At pH = 10, the addition of Tl adatoms on Pt was studied in the electrocatalytic oxidation of glucose.^230^ With an increase in potential from 0.37 to 0.57 V, the formation of 2-keto gluconic acid became more prominent, going from 1 to 14 mM. This indicates that Tl adatoms on Pt promote the electrochemical dehydrogenative oxidation of the C2-OH group of GA (Scheme 10), thereby enhancing the formation of 2-k-GA.^230^ These results are in strong contrast with those obtained with Pt electrodes modified with Tl adatoms under acidic conditions, for which the formation of 2-k-GA was not reported.^69^ Moreover, it is interesting to note that the formation of 5-k-GA was not considered, indicative that the C5-OH group of GA is not dehydrogenated or that this product was overlooked. CV indicated that Tl adatoms on Pt markedly improved the electrocatalyst activity.^230^ This makes Tl adatom-modified Pt electrodes interesting candidates for the production of 2-k-GA, where the Tl surface coverage could potentially be optimized to further improve the electrocatalyst selectivity, as it has been shown for Bi adatoms in the selective oxidation of glycerol.^55^

Under alkaline conditions (pH = 13), modification of Pt electrodes with Pb adatoms did not change the electrocatalyst selectivity in the potential window 0.5 to 0.8 V for the oxidation of mannose and glucose concerning C–C cleavage products,^71,110,212^ while it did for galactose.^217^ For bare metallic Pt and Pb-adatom-modified Pt, similar concentrations were found for mannose oxidation products and glucose oxidation products, whereas at 0.7 V Pb-adatom-modified Pt had a nearly 100% selectivity toward galactonic acid.^217^ It was argued that Pb adatoms preferably occupy poisoning sites on the Pt surface and modify the adsorption coordination, enhancing the stability and altering the selectivity of the electrocatalyst.^217^ Pb was also found to promote the formation of 2-k-GA and 5-k-GA.^71,212^ In the presence of Pb adatoms, the coordination of GA on the Pt surface changes, thereby promoting the dehydrogenative oxidation of C2-OH and C5-OH group of GA and thus the formation of 2-k-GA (33%) and 5-k-GA (8%).^212^ In line with these results, it was shown for sugar alcohol oxidation that Pb adatoms also slightly change the electrocatalytic selectivity of Pt toward the secondary alcohol of glycerol.^200^

A non-alloyed PtBi/C electrocatalyst was used to study the effect of Bi on the selectivity at pH = 13 (0.1 M NaOH).^242^ The dilution of Pt atoms by Bi atoms decreases the likeliness of the multibonded adsorption mode of glucose, thus hindering C–C cleavage reactions and limiting the formation of degradation products.^242^ As a result, the electrocatalyst selectivity is improved and the stability is enhanced, as CO formation is prevented (as confirmed by FTIR) and thus the poisoning diminished. The addition of Bi to Pt decreased the onset potential for the oxidation of 1-O methyl glucoside (i.e., glucose with a protecting group at the anomeric carbon) from 0.42 to 0.22 V, showing that it promotes the dehydrogenative oxidation of the primary alcohol group at a lower potential than monometallic Pt.^91,110,230,242^ The lower onset potential for the C6-OH group can be explained by the fact that Bi atoms adsorb OH^–^ ions at lower potentials, while Pt adsorbs the organic molecule, thus ensuring the vicinity of the two species required to initiate the oxidation of the C6-OH group of glucose.^242^ This effect was also reported for PdBi/C electrocatalysts.^243^ In 6 h of electrolysis at 0.4 V, PtBi/C catalyzes the conversion of glucose and 1-O methyl glucoside with nearly 100% selectivity toward GA and 1-O methyl glucuronate, respectively (as determined by MS, NMR, and HPLC).^242^ This makes PtBi/C an interesting electrocatalyst for electrochemically oxidizing glucose, methyl glucoside, and potentially other saccharides. It is worth noting that these results do not match with those obtained for glycerol oxidation over PtBi < 0.6 V, where it was reported that Bi strongly promotes the oxidation of the secondary alcohol group.^118^

At pH = 10 and 0.5 V, Tl-adatom-modified Pt electrodes can catalyze the selective oxidation of the C6-OH group to convert 31% of 1-O methyl glucoside with 97% selectivity toward methyl glucuronic acid.^230^ Similar results were obtained at pH = 13 and 0.5 V, where metallic Pt_9_Bi_1_ can catalyze the selective oxidation of 40% 1-O methyl glucoside and 37% glucose with nearly 100% Faradaic efficiency and 100% selectivity to methyl glucuronic acid and GA.^242^ We argue that the high selectivity toward methyl glucuronic acid can be attributed to the protection of the anomeric carbon,^230,242^ which can (1) steer the electrocatalyst selectivity toward other reactive groups and (2) prevent the mutarotation of the reactant and thus reduce the accessibility of the reactant for C–C cleavage reactions.

In summary, most studies on the effect of post-transition metals on Pt electrocatalysts for the oxidation of saccharides have been performed under alkaline conditions. However, to discriminate between base-catalyzed and electrode-catalyzed reactions more research should be conducted under acidic conditions (see section 2.1). In addition, the potential at which the effect of post-transition metals on Pt is studied should be chosen more carefully to avoid the oxidation/desorption of the transition metal, thereby losing its contribution to the electrocatalyst performance. Finally, and importantly, only a few studies report the formation of keto-oxidation products over Pt electrodes in the presence of Pb^71,212^ or Tl adatoms,^230^ while other studies neither report nor discuss the formation of these keto-oxidation products over Pt electrodes in the presence of Pb^69,110,217^ or Tl^69^ adatoms. Despite this, it is expected that the formation of keto-oxidation products is strongly promoted by the introduction of post-transition metals on Pt, as was shown for the electrocatalytic oxidation of sugar alcohols. This indicates that many products may have been overlooked, among others 2-k-GA, 5-k-GA, and GD.

#### 5.4.2 Au-Post-Transition Bimetallic Electrocatalysts for the Oxidation of Saccharides

Very few studies describe the influence of post-transition metals on Au electrodes on the oxidation of glucose. These studies were conducted at pH = 13 with Pb,^71,244^ Bi,^71^ and Tl^71^ adatoms. It was shown that metallic Au (at *E* < 0.75 V) can catalyze the selective oxygenative/dehydrogenative oxidation of the anomeric carbon of saccharides (see section 5.1.1), yielding predominantly monocarboxylates (e.g., GA, mannonic acid, and galactonic acid).^28,91,214−217,244^ At *E* = 0.6 V, the addition of Tl adatom on Au electrodes does not seem to affect the selectivity in the conversion of glucose, whereas the addition of Bi adatoms promotes the formation of GLR (12%) but also promotes C–C cleavage reactions.^71^ By contrast, at *E* = 0.6 V the addition of Pb adatoms on Au improved the selectivity toward GLR to 25% without inducing more C–C cleavage reactions.^71,244^ An increase in potential to 0.9 V improved the selectivity further to 35% GLR, while inducing more C–C cleavage reactions.^71,244^ These results indicate Pb and Bi adatoms decrease the onset potential for the electrocatalytic oxidation of the primary alcohol group of glucose.

### 5.5 Other Electrocatalysts for the Oxidation of Saccharides

Li et al. showed that at pH = 13.7 it is possible to oxidize various saccharides (∼100% conversion) to 50–40% lactic acid (arabinose, glucose, and xylose) and 33% lactic acid (fructose) over hierarchical Fe-doped Ni_2_P nanosheets hybridized with C on Ni foam (Fe–Ni_2_P@C/NF).^245^ The reaction pathway to obtain lactic acid is likely induced by non-electrochemical reactions (see section 2.1). Hence, it is difficult to attribute the formation of lactic acid to electrocatalytic reactions.

Under harsh alkaline conditions (pH = 14), the selective oxidation of glucose toward GLR can be achieved with the aid of a nanostructured NiFe-based electrocatalyst on Ni foam at *E* = 1.3 V.^127^ This NiFeO_*x*_ material was synthesized through a hydrothermal treatment of Ni foam with an iron precursor, resulting in Ni(OH)_2_ and FeOOH crystalline phases (determined by XRD). This NiFeO_*x*_ electrocatalyst was used to promote the conversion of 0.01 M glucose (98% conversion) to GLR with a selectivity of 83%. As a reference, a pure Ni foam catalyst was used at 1.3 V, which yielded 37% GA and 17% GLR.^127^ The high selectivity toward GLR obtained with the NiFe-based electrocatalyst is likely related to the harsh alkaline conditions, which were also needed for the production of dicarboxylic acids from glycerol.^105,107,113,160,161^ Moreover, combining Ni with other metals/metal oxides, such as Bi, CeO_2_, SbO_2_, Au, Pd and Fe, dilutes the Ni surface and thereby hinders C–C cleavage reactions, as was shown for the electrocatalytic oxidation of glycerol^120,207,246^ and glucose.^127^ An increase in reaction time to convert higher initial glucose concentrations resulted in lower GA and GLR selectivities, namely 92% (10 mM glucose and 2 h reaction), 87% (50 mM glucose and 10 h reaction), and 71% (100 mM glucose and 18 h reaction).^127^ The longer reaction times and higher initial glucose concentrations are likely to induce more retro-aldol reactions under the studied harsh alkaline conditions (see section 2.1.1), thereby decreasing the selectivity toward C6-oxidation products. Despite this, it was claimed that no significant amount of glucose was degraded at pH = 14 after 24 h.^127^

## 6 Conclusions and Considerations for Future Research

In this contribution, we have critically reviewed the literature on the electrocatalytic oxidation of sugar alcohols and saccharides by discussing the electrochemical pathways while taking into account the (sometimes crucial) contribution of non-electrochemical pathways. Trends were defined on the effect of reaction conditions and electrocatalyst properties on the selectivity and activity in the electrocatalytic oxidation of specific functional groups of sugar alcohols and saccharides for the synthesis of value-added compounds. These trends were compared to identify the most promising routes for the selective production of value-added chemicals through the electrocatalytic oxidation of sugar alcohols and saccharides. In the next paragraphs, we highlight topics that require attention for future research and propose several avenues that are worthy of being further explored.

The main points of attention for future research on the electrocatalytic oxidation of sugar alcohols and saccharides which we identified are: (1) the type of electrolyte and the pH used in electrocatalytic experiments, specifically their influence of non-electrochemical reactions taking place in solution; (2) the analytical technique used to quantify glucose oxidation products; and (3) the use of control tests with (intermediate) products in electrocatalytic experiments.

(1) Most studies on the electrocatalytic oxidation of sugar alcohols and saccharides have been performed in alkaline electrolytes. However, under these reaction conditions base-catalyzed non-electrochemical reactions compete with electrochemical reactions. As a result, it becomes nearly impossible to discriminate between base-catalyzed and electrode-catalyzed reactions, thereby making it difficult to evaluate the property-performance relationship of electrocatalysts. To properly understand the role of base-catalyzed reactions, blank experiments need to be performed with all reactants and (intermediate) products that can be formed in the electrochemical experiments. The effect of time is also very important in this respect, as long reaction times are more likely to enhance the contribution of non-electrochemical reactions. In this context, it is strongly advised to quench (i.e., neutralize) the reaction mixtures before offline analysis (e.g., by HPLC) to minimize non-electrochemical reactions that could take place between the end of the electrochemical experiment and the analysis.^54,247^

The nature of the electrolyte is not always considered to have a strong impact on the electrocatalytic oxidation of sugar alcohols and saccharides. Yet, it was shown that borax and certain cations (Li^+^) can complex with reactants or (intermediate) products, thereby stabilizing specific functional groups and thus affecting the electrocatalyst selectivity. Moreover, certain anions, such as (bi)sulfate, were found to compete with reactants for adsorption on the catalyst surface and, therefore, affect the electrocatalyst activity. In principle, by blocking specific sites at the surface of the electrocatalyst, the anions might also affect the selectivity, though this aspect has not been systematically investigated so far. Therefore, future research should make sure that the effect of certain electrolytes, like borax, is properly understood or consider the use of different anions in a systematic way, to elucidate their property-performance relationship.

(2) Studies conducted on the electrocatalytic oxidation of glucose rarely report the formation of glucose dialdehyde, 2-keto-gluconic acid and 5-keto-gluconic acid, and neither mention the formation of glucuronic acid nor guluronic acid. Thus, it is possible that many of the (intermediate) products on the glucose oxidation pathway are being overlooked and therefore not quantified. However, to study the property-performance relationship of electrocatalysts for the oxidation of glucose, it is needed to quantify (intermediate) products. Therefore, future research needs to use suitable analytical techniques, such as a combination of high pressure anion exchange chromatography with high pressure liquid chromatography,^209^ to quantify all (intermediate) products of the glucose oxidation pathway.

(3) The formation of dicarboxylic acids, such as tartronic acid and mesoxalic acid from glycerol or glucaric acid from glucose, could only be achieved under highly alkaline conditions (pH ≥ 13.7), except for MnO_2_-based electrocatalysts, with which glucaric acid was produced at pH = 0.7–10. This indicates that the selective conversion of specific intermediates is highly limited on certain electrocatalysts. Therefore, future research on the electrocatalytic oxidation of sugar alcohols and saccharides should include chronoamperometric experiments on the (intermediate) products formed. This will enable evaluating the electrocatalyst activity toward (intermediate) products (i.e., different functional groups) and thereby give better insight into the electrocatalyst selectivity and the corresponding limiting reactions.

The most relevant and promising avenues that we identified for future research on the electrocatalytic oxidation of monosaccharides and sugar alcohols into valuable products are: (1) the investigation of the rate-limiting step(s) for the electrocatalytic oxidation of saccharides on Au; (2) the use of post-transition metals to change the catalytic selectivity of Pt for the oxidation of saccharides; (3) the design of electrocatalysts that enable the selective production of dicarboxylic acids under acidic reaction conditions; (4) the investigation of the stability of electrocatalysts when an alternating potential is used to electrochemically oxidize sugar alcohols and saccharides; (5) the influence of metal surface morphology on the reactivity and selectivity toward the oxidation of saccharides and sugar alcohols; and (6) the shift from the current focus on lab-scale investigation to flow cell studies, prolonged tests, and upscaling.

(1) It was shown that the rate-limiting step for the electrocatalytic oxidation of glycerol (i.e., primary alcohol groups oxidation) on Au is base-catalyzed. This insight can be used to tune the pH of the electrolyte in electrochemical cells to improve or limit the electrochemical oxidation of primary alcohol groups from sugar alcohols or saccharides on Au electrodes. In order to steer the reaction toward the electrocatalytic oxygenative/dehydrogenative oxidation of the anomeric carbon of saccharides on Au electrodes, it is recommended to investigate the rate-limiting step(s) of this reaction.

(2) For the electrochemical oxidation of sugar alcohols, post-transition metals effectively change the selectivity of Pt electrocatalysts toward the secondary alcohol groups of glycerol, promoting the formation of dihydroxyacetone. This approach was also found to promote the selective electrocatalytic oxidation of glucose or gluconic acid to 2-keto-gluconic acid and 5-keto-gluconic acid, being precursors for the synthesis of platform molecules like ascorbic acid and tartronate. Yet, only a few studies on post-transition metal-modified Pt electrodes were devoted to the electrocatalytic oxidation of glucose, of which only one was performed under acidic conditions. Therefore, research under acidic conditions is needed to evaluate whether these post-transition metals can be used to change the selectivity of Pt toward oxidation of specific secondary alcohol groups of glucose or gluconic acid, thereby enabling the selective production of 2-keto-gluconic acid or 5-keto-gluconic acid.

(3) The formation of dicarboxylates on electrodes has been achieved nearly exclusively in highly alkaline conditions (pH ≥ 13.7) over a wide range of electrocatalysts (e.g., based on Pt, Au, and NiFeO_*x*_), while MnO_2_ was also reported to promote the formation of dicarboxylic acids under acidic conditions. In this case, MnO_2_-based electrocatalysts were used to promote the electrochemical oxidation of glucose to glucaric acid. The formation of acids is preferred over that of the corresponding dicarboxylate salts as this decreases downstream processing costs and the formation of salt waste streams. Therefore, more research should be devoted to design and develop different electrocatalysts that are stable under acidic conditions for the selective production of dicarboxylic acids.

(4) Multiple studies use cyclic voltammetry or an alternating potential to retain non-equilibrated metal surfaces (i.e., surfaces that do not reach an oxidized steady state), thereby improving the activity of the electrocatalyst. Yet, these alterations in potential can be detrimental for the selectivity of the electrocatalyst and can be complicated to operate in large-scale electrochemical systems. Therefore, it is recommended to evaluate the effect of an alternating potential on the electrocatalyst stability. Moreover, the feasibility of an alternating potential in large-scale electrochemical systems needs to be investigated further.

(5) Although some publications focus on studying the conversion reactions on a specific surface morphology of the Au or Pt electrodes, there is still no systematic fundamental study on the influence of metal surface morphology on the reaction mechanism, activity, and selectivity toward the oxidation of saccharides and sugar alcohols. Future research toward deeper understanding in this direction can enable a rational design of electrode surface morphology based on the aimed added-value product, thus making the process more effective.

(6) Most of the literature on the electrocatalytic oxidation of monosaccharides and sugar alcohols is centered on the activity and selectivity of novel electrocatalysts monitored by CV and LSV, and this was also the focus of this review. In the perspective of a practical and larger-scale application of these electrochemical routes, it is important to study the electrocatalytic performance at longer reaction times (by chronoamperometry or chronopotentiometry), preferably in a flow cell configuration, and to assess the stability of the electrocatalysts under these conditions.

## Abbreviations

FA: formic acid
GALD: glyceraldehyde
DHA: dihydroxyacetone
GLA: glyceric acid
TA: tartronic acid
MOA: mesoxalic acid
HPA: hydropyruvic acid
LA: lactic acid
GD: glucose dialdehyde
GA: gluconic acid
2-k-GA: 2-keto gluconic acid
5-k-GA: 5-keto gluconic acid
GUL: guluronic acid
GLU: glucuronic acid
GLR: glucaric acid
CV: cyclic voltammetry
LSV: linear sweep voltammetry
CA: chronoamperometry
FE: Faradaic efficiency
HPLC: high-pressure liquid chromatography
XANES: X-ray absorption near edge structure
XAS: X-ray absorption spectroscopy
XPS: X-ray photoelectron spectroscopy
XRD: X-ray diffraction
HAADF: high-angle annular dark-field
STEM: scanning transmission electron microscopy
EDS: energy dispersive X-ray spectroscopy
EELS: electron energy loss spectroscopy
